# Identifying Key Predictors of Mid‐Childhood Obesity in a Population‐Based Cohort Study: An Evidence Synthesis and Predictive Modeling Study

**DOI:** 10.1111/obr.13958

**Published:** 2025-06-12

**Authors:** Yuchan Mou, Susana Santos, Macarena Lara, Ivonne P. M. Derks, Vincent W. V. Jaddoe, Romy Gaillard, Janine F. Felix, Eric Steegers, Trudy Voortman, Marinus H. van IJzendoorn, Pauline W. Jansen

**Affiliations:** ^1^ Department of Epidemiology Erasmus MC, University Medical Center Rotterdam Rotterdam The Netherlands; ^2^ The Generation R Study Group Erasmus MC, University Medical Center Rotterdam Rotterdam The Netherlands; ^3^ Department of Pediatrics Erasmus MC, University Medical Center Rotterdam Rotterdam The Netherlands; ^4^ EPIUnit ITR Instituto de Saúde Pública da Universidade do Porto, Universidade do Porto Porto Portugal; ^5^ Department of Psychology, Education and Child Studies Erasmus University Rotterdam Rotterdam The Netherlands; ^6^ Department of Child and Adolescent Psychiatry/Psychology Erasmus MC, University Medical Center Rotterdam Rotterdam The Netherlands; ^7^ Research Department of Behavioural Science and Health University College London London UK; ^8^ Department of Obstetrics and Gynaecology Erasmus MC, University Medical Center Rotterdam Rotterdam The Netherlands; ^9^ Meta‐Research Innovation Center at Stanford (METRICS) Stanford University Stanford California USA; ^10^ Department of Psychiatry Monash University Melbourne Australia; ^11^ Research Department of Clinical, Education and Health Psychology UCL, University of London London UK; ^12^ Faculty of Psychology and Humanities Universidad San Sebastián Valdivia Chile

**Keywords:** BMI, childhood obesity, explained variance, longitudinal population‐based study, prediction model, systematic review

## Abstract

A wide spectrum of predictors of childhood overweight and obesity has been identified over past decades, yet a quantitative overview of multidisciplinary predictors is missing, and the relative importance and their collective contribution to childhood obesity remains unclear. We synthesized evidence from 93 published studies from the Generation R Study, a population‐based prospective cohort from early‐pregnancy onwards in the Netherlands, to provide a quantitative overview of 210 predictors across preconception and childhood associated with body mass index (BMI) in mid‐childhood and selected 59 candidate predictors. Then, we further identified 32 key predictors for either zBMI or weight status using model search algorithms and built prediction models with data from the same cohort. Associations of note were identified among predictors in preconception and prenatal parental health, early‐life weight, and child behavior domains. An interquartile range increase in parental prepregnancy BMI (maternal *β* = 0.17, 95% CI: 0.14, 0.20; paternal *β* = 0.17, 95% CI: 0.14, 0.21), early‐life weight (e.g., zBMI at age 2y *β* = 0.28, 95% CI: 0.23, 0.33), and food responsiveness (*β* = 0.25, 95% CI: 0.21, 0.28) were positively associated with mid‐childhood BMI, and satiety responsiveness was negatively associated (*β* = −0.18, 95% CI: −0.21, −0.15). Together, identified key predictors accounted for 49.2% of the variance of BMI and 33.7% of the variance of the odds of overweight and obesity at the age of 10 years. Multimodal interventions targeting parental BMI before pregnancy, early adiposity rebound, and management of appetite in early childhood may be most effective in maintaining a healthy weight during childhood.

**Abbreviations**
25(OH)D: 25‐hydroxyvitamin D
AA: arachidonic acid
BMI: body mass index
BSI: Brief Symptom Inventory
CEBQ: Children's Eating Behavior Questionnaire
CFQ: Child Feeding Questionnaire
DSA: deletion–substitution–addition
DTA: docosatetraenoic acid
FFQ: Food Frequency Questionnaire
GSI: Global Severity Index
*h* ^2^
: narrow‐sense heritability
LC‐MS: liquid chromatography–tandem mass spectrometry
PRS: polygenic risk score
PUFA: polysaturated fatty acids
*R* ^2^
: coefficient of determination
SDS: standardized deviation score
SNP: single nucleotide polymorphism

## 1 Introduction

Childhood obesity is a major, global public health problem. Although the rising trends have plateaued in many developed countries, the prevalence of childhood obesity remains alarmingly high [1]. This concern is amplified by evidence that child overweight and obesity often persists into adulthood [2, 3]. Greater body mass index (BMI) and adiposity are associated with an increased risk of psychological problems [4, 5] and with disturbances in cardiometabolic health indicators throughout the life course [6], including cardiovascular diseases and Type 2 diabetes, which can lead to premature death [7, 8].

Identifying key predictors is an important step towards early detection of childhood obesity and monitoring of those at risk of obesity. In the past two decades, epidemiological studies have investigated a wide spectrum of predictors of overweight and obesity at different ages in childhood, spanning from genetic, demographic, socioeconomic, environmental to behavioral factors. A comprehensive overview of these candidate predictors and their effect sizes from preconception onwards will provide evidence for predicting child BMI from a life course perspective. Indeed, many systematic reviews and meta‐analyses collected evidence for candidate predictors of child BMI, yet these overviews did not uncover predictors exhaustively. On the one hand, some systematic reviews evaluated evidence for various predictors in a narrative form [9, 10] and thereby lacked quantitative assessment of effect. On the other hand, meta‐analyses estimate overall measures of effect and typically included only a limited number of predictors, often focusing on a cluster of predictors, such as dietary factors or physical activity [11]. As such, a quantitative overview of predictors from a multidisciplinary perspective is missing. Therefore, we propose to review predictors in a single longitudinal cohort to go beyond a traditional review, including multidisciplinary predictors measured with the advantage of homogeneity of study design and sampling, despite potential limitations in generalizability.

Another important gap in the identification of key predictors of childhood obesity is that predictors are often examined independently in epidemiological studies, making it hard to draw conclusion on the importance of predictors and their collective contribution to childhood obesity. Joint testing of multiple predictors may reveal the ones that are most strongly, and independently of other factors, associated with child BMI, as well as estimate the extent to which key predictors contribute to variation in child BMI. Research into multi‐exposures and their associations with childhood obesity has been explored within the exposome field to identify relevant environmental features [12]. Environmental features constitute one aspect of the multifactorial influences contributing to childhood obesity. However, a more comprehensive perspective including individual behavioral characteristics remains largely unexplored. Jointly testing all potential predictors of a health outcome presents a significant conceptual and data feasibility challenge. Through evidence synthesis and predictive modeling within a single cohort, we can employ both hypothesis‐driven and exploratory approaches to identify key predictors and their cumulative explanation of the variance in a specific health outcome measured in the same cohort across time. This approach maximizes within‐study variation by minimizing between‐study variability that might often be difficult to explain in regular meta‐analytic syntheses of several independent samples. The single‐cohort approach may increase internal validity at the expense of external validity, and the generalizability of the resulting prediction model should be tested in (the combination of) other cohort studies.

In this study, we aimed to integrate domain‐specific knowledge by using published studies from the Generation R Study, a large prospective cohort with measurements encompassing genetic, sociodemographic, and behavioral factors from fetal life onwards, to facilitate selecting predictors from a multidisciplinary view. We aimed (1) to provide a quantitative overview of predictors across pregnancy and childhood associated with BMI in mid‐childhood, (2) to identify which of these predictors are most relevant, and (3) to explore how much variance in child BMI these key predictors together explain. First, we performed a systematic review of Generation R papers describing associations of any predictor with child BMI and quantitatively synthesized the evidence to present the magnitudes of associations. Second, based on the results from the quantitative synthesis, we selected candidate predictors using a predefined magnitude of effect estimates, and we used an iterative model search algorithm to further select a subset of the most relevant predictors using data from the Generation R Study. We assessed the joint associations between these key predictors with child BMI and weight status in mid‐childhood. We focused on BMI and weight status in middle childhood because this stage represents a critical developmental period when obesity and its related behaviors persist into adulthood [3, 13, 14]. Moreover, obesity in mid‐childhood is associated with a range of short and long‐term health outcomes, including cardiometabolic health, as well as psychosocial outcomes later in life [15, 16]. Identifying predictors of mid‐childhood BMI is therefore crucial for uncovering intervention opportunities that can have sustained, long‐term health benefits.

## 2 Methods

### 2.1 Study Population

The Generation R Study is an ongoing population‐based prospective cohort from early fetal life onwards in Rotterdam, the Netherlands [17], which is designed to identify early environmental and genetic determinants of growth, development, and health during the life course. Of the included pregnant women with a delivery date between April 2002 to January 2006, 7393 mother–child pairs consented for follow‐up in the mid‐childhood. BMI at age 10 years was measured in 5686 children. The study was approved by the Medical Ethics Committee of Erasmus MC, University Medical Center Rotterdam. Written informed consent was obtained from parents of all participating children.

### 2.2 Evidence Synthesis

#### 2.2.1 Study Selection

All published studies using data from the Generation R Study were eligible. We searched for literature in the electronic databases: PubMed, Embase, Ovid MEDLINE, and Web of Science. The search strategy consisted of combinations of the names of main researchers of the Generation R Study to ensure inclusion of all the Generation R Study publications for screening, including those that examined BMI in additional or secondary analyses instead of in main analyses (see detailed information of search strategies in Supplementary Methods S1). Searches were restricted to studies published until October 31, 2023.

**FIGURE 1:**
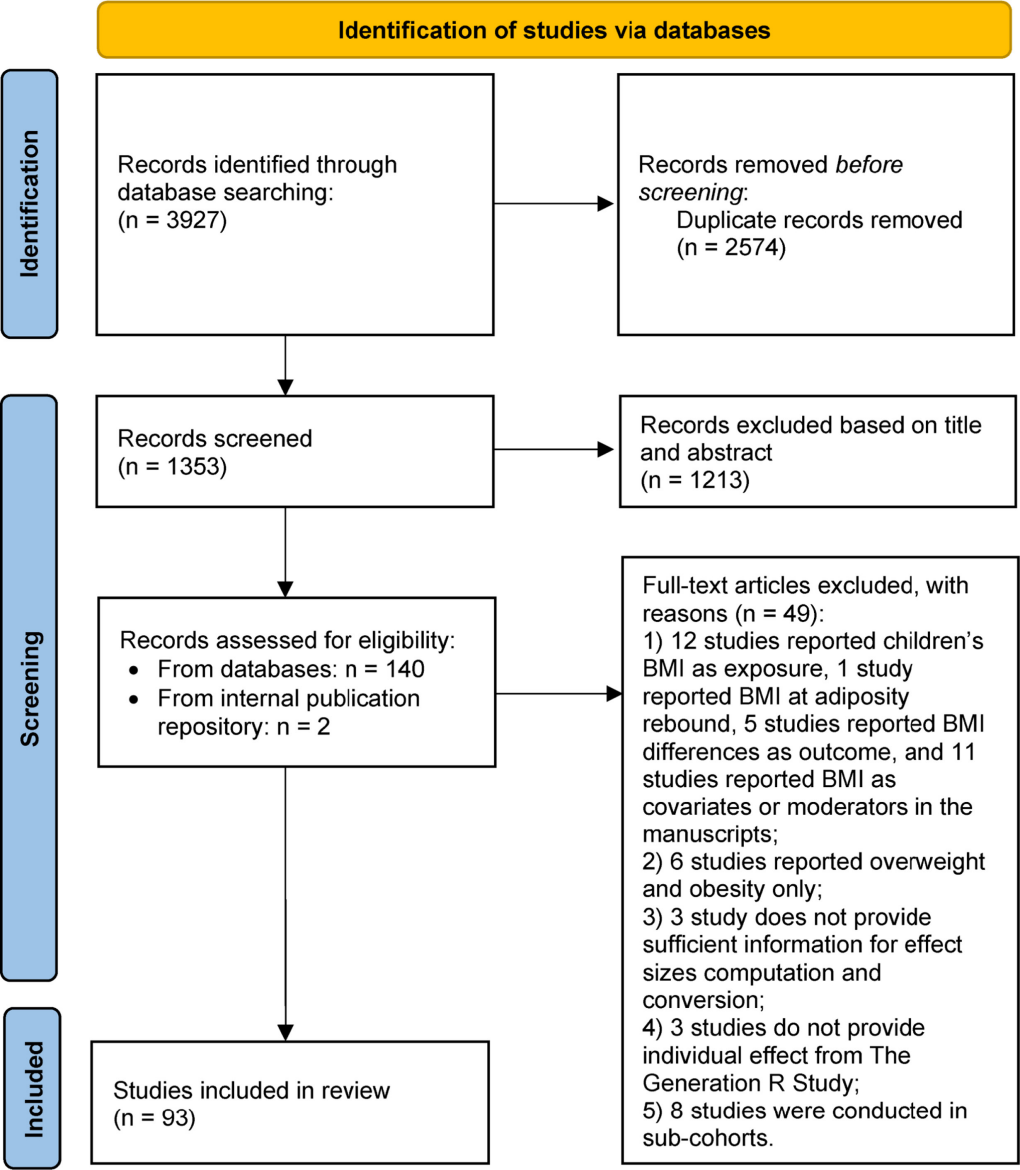
PRISMA flow diagram.

We defined the eligibility criteria prior to the study. Studies were included if they (1) used primary data from the Generation R Study; (2) were conducted in the full Generation R Study sample, that is, studies on sub‐cohorts with specific selection criteria applied were excluded (e.g., the Focus cohort of mother and child pairs (*n* = 1232), a sample of families of Dutch origin selected for in‐depth assessments) [17]; (3) assessed child BMI or *z*BMI as an outcome between the ages of 2 and 10 years; and (4) reported adequate details to perform the calculation of effect estimates. Duplicates were removed before screening. Three reviewers (Y.M., M.L., and T.V.) screened independently. First, titles and abstracts were screened. Second, full texts of selected papers were reviewed based on inclusion criteria; each step was done by two reviewers. Disagreements on study eligibility were resolved via discussion between reviewers, or with senior researchers (M.v.I. and P.J.) when uncertainty persisted. In addition, we conducted a cross‐checking with the Generation R Study internal publication repository during the full‐text screening stage and added papers that were missed during the database search. The screening process is presented in a PRISMA 2020 flow diagram [18] (Figure 1).

#### 2.2.2 Data Extraction

The extracted information included the name of first author, year of publication, measurement of predictors, ages at assessment of predictors and outcomes, standard deviations of predictors and outcomes necessary for data conversion, sample size (total sample size, and sample sizes in reference group and comparison groups when available), covariates, statistical analysis performed, effect measures, and, if available, effect estimates. Six reviewers (Y.M., M.L., I.D., T.V., M.v.I., and P.J.) independently extracted data using a standardized data extraction form. All extracted data were checked by the first author (Y.M.) for accuracy. Additional decisions were made when extracting data: (1) If multiple measurements of BMI were reported separately in one study, only the effect estimates for BMI at the latest time point (i.e., the oldest age) were extracted to use data spanning the largest time window; (2) moderation effect estimates (i.e., effect modification) were not included as our focus was on main effects; (3) effect estimates of continuous (or ordinal) predictors were prioritized over categorical predictors; (4) when the standard deviation of BMI was not presented in a study, interquartile range, 90% or 95% range (depending on whichever presented) was extracted; (5) effect estimates were extracted from the mean differences, bivariate correlation matrix, basic models, and confounder‐adjusted models, with the unadjusted effect sizes (mean differences, bivariate correlations, or basic/unadjusted models) being prioritized to enhance comparability of effect sizes across papers. When no unadjusted effect estimates were presented, the models most minimally adjusted for confounders were considered as proxies to unadjusted effect sizes. For studies with multiple comparison groups, the number of participants in the reference group was divided evenly among the comparisons to address the unit‐of‐analysis error [19].

#### 2.2.3 Quantitative Syntheses

All reported effect estimates—*β* (standardized regression coefficient), *B* (unstandardized regression coefficient), mean, median, and *r* (correlation)—were converted to Fisher's *z* to facilitate confidence interval computation in the quantitative synthesis. All conversions were done in R 3.6.1 using the “esc” package [20] and customized R functions [21, 22, 23] and are summarized in Table S1 and Table S2.

We used the random‐effect model to pool the effect estimates of predictors representing a similar construct that were assessed at different ages (e.g., effect estimates of food responsiveness at ages 4 and 10 years on child BMI at age 10 years were pooled) [24], because we assume effect estimates of a predictor may vary between studies exploring different developmental stages. We used Fishers' *z* to pool effect estimates of predictors, and then converted them to Cohen's *d* (95% CI) to present the summary effect sizes.

Considering the large variety of predictors included and that effect estimates yielded in a large population‐based cohort study are generally small, we defined |*d*| ≥ 0.1 as the threshold value of effect sizes for selecting candidate predictors with substantial effect. We clustered candidate predictors into nine domains. Forest plots were constructed for candidate predictors. Quantitative syntheses were run in R 3.6.1 using the “meta” package, and forest plots were constructed using the “ggplot2” package.

### 2.3 Predictive Modeling

Based on the results of quantitative synthesis, prediction models of BMI and weight status were built using data from the Generation R Study.

#### 2.3.1 Childhood Obesity Outcomes

At the age of 10 years, children's weight was measured without shoes and heavy clothing, using a mechanical personal scale (SECA, Almere, the Netherlands), and height was measured with a Harpenden stadiometer (Holtain Limited, DYFED, UK) by trained staff during visits to the Generation R research center at the Erasmus MC using standard procedures. BMI was calculated as weight/height [2] (kg/m^2^), and we further calculated age‐ and sex‐specific standard BMI *z*‐scores (*z*BMI) according to the Dutch reference growth curves [25] constructed using the LMS method with data from a nationwide growth study sampling and measuring children up to 20 years of age in 1996 and 1997, and we categorized BMI into weight status according to the International Obesity Task Force (IOTF) cutoffs [26]. We chose to use zBMI as an outcome because it allows us to highlight predictors that contribute to deviations from typical growth patterns within the Dutch population, which is more relevant for identifying at‐risk children and public health interventions. Furthermore, it allows comparison between studies.

#### 2.3.2 Measurement of Selected Candidate Predictors

The detailed description on how information of the selected candidate predictors was obtained can be found in the Supplementary Methods S2.

#### 2.3.3 Statistical Analysis

We applied multiple imputation to impute the missing values of candidate predictors, except child BMI polygenic risk score (PRS). The detailed method for multiple imputation is described in the Supplementary Methods S3. Table S3 presents the percentage of missing values for each predictor, as well as the variables used to impute these missing data.

We used the deletion–substitution–addition (DSA) algorithm [27] to identify key predictors from candidate predictors selected in evidence synthesis and performed multiple linear regression model. The DSA algorithm is an aggressive iterative model search and a loss‐based cross‐validated algorithm that works by deleting a predictor, substituting one predictor by another, or adding a predictor to the model at each iteration [28]. At the end of each iteration, a final model is selected by minimizing the value of the root mean squared error of prediction. It has been used in exposome studies [29] and has been shown to have a lower false positive rate in comparison with other linear regression‐based variable selection algorithms in a simulation study [30]. Repeating the predictor selection procedure with cross‐validation can result in different sets of predictors in the models. To get a stable predictor selection, we fitted fivefold cross‐validated DSA 50 times. We performed DSA on *z*BMI and weight status as outcomes separately. Predictors that were retained in at least 10% of the DSA models were selected to fit final multiple linear regression models for childhood BMI or logistic regression models for child weight status. The DSA was applied in stacked data with 20 imputed datasets allowed up to 25 variables in the final model [31].

After finalizing the list of key predictors, we applied multiple linear regression to fit prediction models for child BMI and logistic regression to model child weight status. All identified key predictors entered the models simultaneously. Overall explained variance of the fitted linear regression models for childhood BMI was quantified by the coefficient of determination (*R* ^2^). Explained variance of the fitted logistic regression models for child weight status (overweight and obesity vs. normal weight and underweight) was quantified by McFadden's pseudo *R* ^2^ [32]. We further quantified relative importance of the predictors by estimating the decompositions of *R* ^2^ in linear regression with the “Lindeman, Merenda, and Gold” method [33] that are implemented in the “relaimpo” R package [34]. This method proposed to average order‐dependent *R* ^2^ allocation across all possible ordering for a fair unique assessment of *R* ^2^ for each predictor. We estimated the relative importance of the predictors in the logistic regression with the relative explained variation metric [35] that are implemented in the “rms” R package [36], which is an identical measure to relative *R* ^2^ if the original model was ordinary least square.

Before entering the algorithm, all continuous predictors were normalized by interquartile range (IQR). As such, one unit increase in each predictor corresponds to an IQR change. We did not allow interaction or non‐linear terms in the DSA model search due to the concerns regarding statistical power overload.

We performed several sensitivity analyses for models of childhood BMI and weight status. First, we added child BMI PRS in the models to account for genetic influences. Second, the models were stratified by sex to obtain sex‐specific estimates. Third, the models were stratified by child ethnic background to obtain ethnicity‐specific estimates. Fourth, to explore parsimonious models, we conducted an additional model selection using DSA based on cross‐validation risk estimates [37], which provide insights into model sizes and complexity. Based on a visual inspection of cross‐validation risk plots for child BMI and weight status, we determined an optimal model size. Subsequently, we fitted parsimonious multiple regression models. Last, to evaluate whether including all candidate predictors would increase the explained variance, we conducted analyses using all candidate predictors in the models. All statistical analyses were performed with R Version 4.1.2 (R Foundation for Statistical Computing, Vienna, Austria).

## 3 Results

### 3.1 Results of the Evidence Synthesis

Figure 1 presents the study selection in the PRISMA flowchart. In total, 142 full‐text articles were screened based on the eligibility assessment. Among them, 49 studies were excluded because (1) 29 studies did not report BMI as an outcome; (2) 6 studies reported effect estimates for overweight and obesity only; (3) 3 studies did not provide sufficient information for effect estimates computation and conversion; (4) 3 studies did not provide an individual effect for Generation R data; and (5) 8 studies were conducted in sub‐cohorts of the study. In total, the selection yielded 93 studies that were conducted within the Generation R Study and used children's BMI or *z*BMI as an outcome and met the inclusion criteria.

An overview of study characteristics is summarized in Table S4, along with its references (reference list of publications included in the evidence synthesis). In total, 210 predictors were reported in different studies. Ages at assessment of the candidate predictors ranged from preconception to 10 years, and children's age at BMI assessment ranged from 2 to 10 years. We illustrated the summary forest plots for predictors with pooled effect sizes above the threshold |*d*| ≥ 0.1 in Figure S1. Below, we summarize the results of the meta‐analyses per domain.

#### 3.1.1 Sociodemographic Predictors

Sociodemographic predictors were reported in 11 studies, which together explored 5 predictors (Table S5). Maternal education, assessed both during pregnancy and when children were 6 years old, had the largest effect (*d* = 0.57) on child BMI at age 10 years, and paternal education, measured at the same time points, had an effect size of *d* = 0.39. Child ethnicity was associated with child BMI at age 6 years with *d* = 0.37.

#### 3.1.2 Preconception and Prenatal Parental Health

Prenatal predictors were examined in 21 studies, which explored 22 predictors (Table S6). Maternal prepregnancy weight and maternal and paternal prepregnancy BMI each had pooled effect sizes larger than *d* = 0.10 on child BMI at age 2–10 years, ranging from 0.10 to 0.31. Maternal smoking assessed in three trimesters of pregnancy (*d* = 0.10) and paternal smoking assessed in early pregnancy (*d* = 0.13) predicted higher child BMI at 6 years. Maternal cannabis use (*d* = 0.17) and paternal cannabis (*d* = 0.13) and tobacco use (*d* = 0.13), both assessed in early pregnancy, predicted higher child BMI at 10 years.

#### 3.1.3 Maternal Nutritional Predictors

Maternal nutritional predictors were examined in 13 studies, which explored 31 predictors (Table S7). Maternal vomiting during the first trimester had the largest effect (*d* = 0.26) on child BMI at age 6 years. As for dietary intake during pregnancy, milk intake during early pregnancy (*d* = 0.15), plasma total n‐6 polysaturated fatty acids (PUFA) concentration (*d* = 0.11), and higher adherence to a vegetable, fish, and oil dietary pattern during pregnancy (*d* = 0.10) predicted higher child BMI at age 6. Plasma arachidonic acid (AA) (*d* = −0.11) and docosatetraenoic acid (DTA) (*d* = −0.10) concentration predicted a lower child BMI at age 2, and 25‐hydroxyvitamin D (25(OH) D) (*d* = −0.11) concentration during pregnancy predicted a lower child BMI at age 6 years. Children whose mothers started to use folic acid supplement preconceptionally or in the first 10 weeks had lower BMI than those of mothers never used folic acid supplement (*d* = −0.11).

#### 3.1.4 Genetic Predictors

Genetic predictors were examined in 7 studies, which explored 58 predictors (Table S8). The effect sizes of 2 predictors were above the cut‐off: rs7185735, a common single nucleotide polymorphisms (SNP) of the fat mass and obesity‐associated (FTO) gene (*d* = 0.12), and the hypothalamic expression and regulation PRS (*d* = 0.10), which is a weighted PRS of a subset of the SNPs identified to be associated with BMI in adults that are annotated to genes with a potential role in these processes.

#### 3.1.5 Maternal Mental Health and Parenting Predictors

Maternal mental health and parenting predictors were examined in 8 studies, which explored 14 predictors (Table S9). Maternal psychopathology symptoms during pregnancy had the largest effect size (*d* = 0.42) in its association with child BMI at age 10 years. Maternal postpartum depression also predicted a higher child BMI at age 3 (*d* = 0.13). Breastfeeding duration, timing of first introduction of solid foods, and restrictive feeding practices at ages 4 and 10 years were linked to a higher child BMI at age 10 (*d* range from 0.12 to 0.22), whereas the pressure to eat feeding practice at ages 2 and 4 years was associated with a lower subsequent child BMI (*d* = −0.12 and −0.13, for ages 4 and 6, respectively).

#### 3.1.6 Early‐Life Weight and Weight Gain Predictors

Early‐life weight and weight gain predictors were examined in 7 studies, which explored 17 predictors (Table S10). For child anthropometrics, BMI at 1.5 months and 2 years and BMI change across infancy (6–24 months) were associated with a higher child BMI at 6 years (*d* = 0.22, 0.58, and 0.16, respectively). Higher central‐to‐total fat mass ratio and total subcutaneous fat mass in infancy were also related to higher child BMI at 6 years. For child growth patterns, higher weight gain during infancy (0–24 months), small‐for‐gestational‐age combined with catch‐up growth or large‐for‐gestational‐age combined without catch down growth (0–24 months), higher peak weight velocity (1–36 months), and higher BMI at estimated adiposity peak were associated with a higher subsequent child BMI at 4 or 6 years of age (*d* range from 0.12 to 0.58). Fetal growth restriction was associated with a lower child BMI at age 6 (*d* = −0.79).

#### 3.1.7 Infant and Childhood Nutritional Predictors

Infant and childhood nutritional predictors were examined in 9 studies, which explored 24 predictors (Table S11). Higher adherence to fat mass index and fat‐free mass index derived dietary pattern (*d* = 0.14) during infancy predicted a higher child BMI at 6 years of age. Higher relative intake of animal proteins (*d* = 0.13) or plant proteins (*d* = 0.14) during infancy predicted a higher child BMI at age 10.

#### 3.1.8 Child Behavioral Predictors

Child behavioral predictors were examined in 14 studies, which examined 26 predictors (Table S12). Among eating behaviors, breakfast skipping (*d* = 0.32) and lunch skipping (*d* = 0.19) at ages 4 and 6 years had the largest effect sizes on BMI at age 6. These were followed by eating behavior subscales assessed at 4 and 10 years, which were associated with concurrent or subsequent child BMI. The subscales included emotional overeating, emotional undereating, enjoyment of food, food responsiveness, and satiety responsiveness, with *d* ranging from −0.10 for emotional undereating to 0.26 for food responsiveness. Among sedentary behavior and physical activity, TV viewing (*d* = 0.19), computer gaming (*d* = 0.12), and outdoor playing (*d* = 0.12) assessed at age 6 years were associated with BMI at the same age. Better set‐shifting ability at age 4 years was associated with lower child BMI at 10 years (*d* = −0.35).

#### 3.1.9 Biological Predictors

In 2 studies, 3 biological predictors were reported (Table S13). Among them, child cortisone concentration (*d* = 0.23) at age 6 years was associated with higher child BMI at 6 years, and child cortisol concentration at age 6 years was associated with higher child BMI at both 6 years (*d* = 0.6) and 10 years (*d* = 0.7).

### 3.2 Results of the Prediction Models

Among predictors for which the association with child BMI reflected an effect size of |*d*| ≥ 0.1, we selected 59 candidate predictors for prediction model building. The decision was made depending on the feasibility of data collection in the real‐world setting (e.g., peak weight velocity was estimated, thus was not selected). When faced with several composite measurements of molecular predictors, we chose aggregated measurements. For instance, considering AA and DTA as components of total n‐6 PUFA, only the total n‐6 PUFA was considered for inclusion. As for predictors in the genetic domain, we replaced them with child BMI PRS which was constructed using identified genetic loci associated with childhood BMI in a large consortium study. The 59 candidate predictors are categorized into 9 domains (Table 1).

**TABLE 1:** Overview of the candidate predictors in each domain.

Domains	Assessment methods	Candidate predictors	Number of variables
Sociodemographic factors	Questionnaires	Maternal and paternal education, child ethnicity	5
Preconception and prenatal parental health	Questionnaires	Maternal prepregnancy BMI, paternal BMI, maternal prepregnancy weight, maternal and paternal smoking during pregnancy, maternal and paternal cannabis use	7
Maternal nutrition during pregnancy	Blood sample and questionnaires (FFQs)	Total n‐6 (omega‐6) polyunsaturated fatty acids, vitamin D status during pregnancy, folic acid supplement use, nausea and vomiting during pregnancy, milk intake during pregnancy	5
Maternal mental health and parenting	Questionnaires (including BSI and CFQ)	Psychopathological symptoms (GSI), depression, feeding practices (pressure to eat and restriction), the age at the first solid foods, breastfeeding duration	10
Early‐life weight and weight gain	Objective measurements	Weight gain during infancy, BMI at 1.5 months and 2 years, BMI gain in mid‐infancy and late infancy, BMI at estimated adiposity peak	8
Infant and childhood nutrition	Questionnaires (FFQs)	High fat mass dietary pattern, animal and plant protein intake	2
Child behaviors	Questionnaires (including CEBQ)	Emotional undereating, emotional overeating, enjoyment of food, food responsiveness, satiety responsiveness, meal skipping (breakfast, lunch and dinner), computer gaming, television viewing, outdoor play, set‐shifting	19
Biological factors	Hair sample	Cortisol concentration, cortisone concentration	2
Genetic variants	Cord blood sample	Child BMI polygenic risk score	1
Total			59

Abbreviations: BSI, Brief Symptoms Inventory; CEBQ, Child Eating Behavior Questionnaire; CFQ, Child Feeding Questionnaire; FFQ, Food Frequency Questionnaire; GSI, Global Severity Index.

**FIGURE 2:**
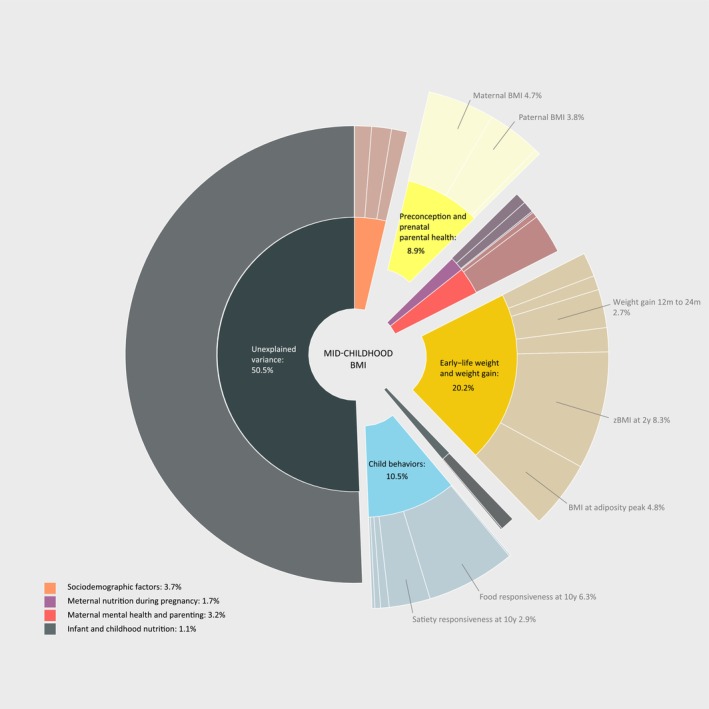
Explained variance in child BMI at age 10 year. This figure illustrates the relative explained variance by 25 key predictors selected for BMI at age 10 years. Each segment of the outer level of the pie chart corresponds to predictors of zBMI listed in sequential order as presented in Table 3. The key predictors explained 49.2% of the variance in zBMI at age 10. The decomposition of *R* ^2^ shows that key predictors in preconception and prenatal parental health (8.9%), early‐life weight and weight gain (20.2%), and child behavior (10.5%) domains accounted for the largest proportions of explained variance, with maternal prepregnancy BMI, paternal BMI, child BMI at age 2, BMI at adiposity peak, food responsiveness, and satiety responsiveness at age 10 each constituting a substantial portion within their respective domains.

Of 5686 children included, 50.4% was girls, 60.1% had a Dutch ethnic background, and 18.0% had overweight or obesity (Table 2). More than half of the parents were highly educated (university degree) in this study population (Table S14). The IQR of all candidate predictors, used as the reference unit of change in the multiple regression analysis, is reported in Table S14. Out of the 59 candidate predictors, the final multi‐predictor DSA models of *z*BMI selected 25 key predictors (Table 3, *z*BMI), including child ethnicity, both parents' education and BMI, maternal smoking during pregnancy, vitamin D concentration during pregnancy, folic acid supplement use, milk intake during pregnancy, mother's feeding practices (pressure to eat and restriction), early‐life weight characteristics (child weight gain and BMI during infancy), high fat mass dietary pattern and protein intake at age 1, and child behaviors (enjoyment of food, food responsiveness, satiety responsiveness, breakfast skipping, and set‐shifting). Altogether, these key predictors explained 49.2% (95% CI: 47.0%, 51.4%) of the variance in *z*BMI at age 10. The decomposition of *R* ^2^ shows that key predictors in preconception and prenatal parental health (8.9%), early‐life weight and weight gain (20.2%), and child behavior (10.5%) domains accounted for the largest proportions of explained variance in mid‐childhood *z*BMI (Figure 2), with maternal prepregnancy BMI, paternal BMI, child *z*BMI at age 2, BMI at adiposity peak, food responsiveness, and satiety responsiveness at age 10 each constituting a substantial portion within their respective domains.

**TABLE 2:** Characteristics of study population (*N* = 5686).

	Mean (SD) or *N* (%)
Age of the child at BMI assessment	9.8 (0.3)
Child sex (girls)	2866 (50.4%)
Child ethnic background	
Dutch	3417 (60.1%)
Non‐Dutch Western	517 (9.1%)
Non‐Dutch non‐Western	1752 (30.8%)
Child BMI at the age of 10 years	17.6 (2.8)
Child *z*BMI at the age of 10 years	0.2 (1.0)
Child weight status	
Normal or underweight^a^	4663 (82.0%)
Overweight or obesity	1023 (18.0%)

*Note:* Values are mean (SD) for continuous variables with a normal distribution, or valid numbers *N* (%) for categorical variables. Missing values of covariates were imputed with multiple imputation (20 imputations).

Abbreviation: SD, standard deviation.

^a^ The proportion of thinness grade 2 and grade 3 in this population is 0.8%.

**TABLE 3:** Associations between key predictors and *z*BMI or overweight and obesity at age 10 years in multiple regression models (*N* = 5686).

Key predictors (IQR or reference category)	Time of assessment	*z*BMI	Overweight or obesity
		β (95% CI)	OR (95% CI)
Sociodemographic factors			
Child ethnic background (ref: Dutch)	Baseline		
Non‐Dutch Western		0.13 (0.05, 0.22)	1.76 (1.27, 2.44)
Non‐Dutch Non‐Western		0.12 (0.05, 0.19)	1.65 (1.26, 2.17)
Maternal education (ref: high)	Baseline		
Middle		—	1.49 (1.17, 1.91)
Low		—	1.68 (1.22, 2.32)
Paternal education (ref: high)	Baseline		1.52 (1.17, 1.98)
Middle		—	1.51 (1.11, 2.05)
Low		—	1.49 (1.17, 1.91)
Maternal education (ref: high)	5 years		
Middle		0.12 (0.06, 0.17)	—
Low		0.20 (0.10, 0.29)	—
Paternal education (ref: high)	5 years		
Middle		0.09 (0.03, 0.15)	—
Low		0.09 (0.01, 0.17)	—
Preconception and prenatal parental health			
Maternal BMI (4.4 kg/m^2^)	Before pregnancy	0.17 (0.14, 0.20)	1.46 (1.31, 1.62)
Paternal BMI (4.2 kg/m^2^)	Baseline	0.17 (0.14, 0.21)	1.49 (1.30, 1.70)
Maternal smoking (ref: no)	During pregnancy		
Until pregnancy		0.03 (−0.05, 0.11)	1.12 (0.82, 1.55)
Continued		0.11 (0.05, 0.17)	1.26 (0.97, 1.63)
Maternal nutrition during pregnancy			
Total n‐6 polyunsaturated fatty acids percentage by weight of total sum of fatty acids (3.3%)	During pregnancy	—	1.18 (1.01, 1.37)
Vitamin D concentration, 25(OH)D (48.4 nmol/L)	During pregnancy	−0.08 (−0.12, −0.04)	0.76 (0.63, 0.91)
Folic acid supplement use (ref: start periconceptional)	During pregnancy		
Start first 10 weeks		0.08 (0.02, 0.13)	1.26 (0.98, 1.61)
Never		0.05 (−0.04, 0.14)	1.25 (0.91, 1.71)
Milk intake (ref: < 1 glasses)	During pregnancy		
1–< 2 glasses		0.01 (−0.05, 0.07)	0.90 (0.69, 1.17)
2–< 3 glasses		0.01 (−0.05, 0.07)	0.82 (0.61, 1.10)
≥ 3 glasses		0.05 (−0.03, 0.13)	1.11 (0.8, 1.53)
Maternal mental health and parenting			
The timing of introduction of solid foods (ref: ≥ 5 months)	During infancy		
4–< 5 months		—	1.17 (0.86, 1.60)
< 4 months		—	1.21 (0.83, 1.76)
Breastfeeding duration (ref: ≥ 6 months)	During infancy		
4–< 6 months		—	1.11 (0.78, 1.59)
2–< 4 months		—	0.99 (0.72, 1.35)
< 2 months		—	0.92 (0.67, 1.26)
Pressure to eat (6 units of sum score)	4 years	−0.05 (−0.08, −0.01)	—
Restriction (9 units of sum score)	10 years	0.18 (0.13, 0.22)	1.56 (1.31, 1.85)
Early‐life weight and weight gain			
Weight gain during infancy (1110 g)	0–6 months	0.07 (0.03, 0.11)	—
Weight gain during infancy (760 g)	6–12 months	0.07 (0.03, 0.10)	1.19 (1.05, 1.35)
Weight gain during infancy (1100 g)	12–24 months	0.10 (0.07, 0.14)	1.28 (1.12, 1.47)
zBMI (1.3 SD)	1.5 months	0.08 (0.04, 0.12)	1.29 (1.11, 1.50)
zBMI (1.4 SD)	2 years	0.28 (0.23, 0.33)	1.86 (1.58, 2.19)
BMI at estimated adiposity peak (1.1 kg/m^2^)	—	0.17 (0.12, 0.23)	—
Infant and childhood nutrition			
High fat mass dietary pattern (1.2 unit of component scores)	1 year	0.06 (0.01, 0.10)	1.14 (0.96, 1.34)
Animal and plant protein intake (15.8 g)	1 year	0.03 (−0.01, 0.06)	1.08 (0.95, 1.23)
Child behaviors			
Enjoyment of food (4 units of sum score)	4 years	−0.05 (−0.09, −0.01)	—
Emotional overeating (4 units of sum score)	10 years	—	1.13 (0.98, 1.31)
Food responsiveness (5 units of sum score)	10 years	0.25 (0.21, 0.28)	2.14 (1.89, 2.42)
Satiety responsiveness (8 units of sum score)	10 years	−0.18 (−0.21, −0.15)	0.64 (0.56, 0.74)
Breakfast skipping (ref: no)	6 years	0.22 (0.12, 0.32)	1.79 (1.26, 2.54)
Television viewing (ref: < 2 h/day)	6 years	0.08 (0.03, 0.13)	—
Outdoor play (ref: > 1 h/day)	6 years	—	0.89 (0.66, 1.20)
Set‐shifting (10 units of sum score)	4 years	−0.06 (−0.10, −0.03)	0.84 (0.72, 0.98)
Explained variance (*R* ^2^)		49.2% (47.0%, 51.4%)	33.7%^a^

*Note:* Predictors selected in at least 10% DSA models were identified as key predictors. As the DSA algorithm was performed separately for zBMI and weight status, a set of key predictors was identified for each outcome. These key predictors were then included simultaneously in the multiple linear regression model for zBMI and the logistic regression model for weight status, respectively. Predictors not modeled in a given outcome were marked with a “—” in the table. For continuous variables, β represent differences in *z*BMI per interquartile range increase in predictors; for categorical variables, β represent differences of *z*BMI in comparison category versus reference categories. For continuous variables, OR represent differences in odds of overweight or obesity per interquartile range increase in predictors; for categorical variables, OR represent differences of odds of overweight or obesity in comparison category versus reference categories. IQR and reference categories are indicated in the bracket for predictors.

Abbreviations: BMI, body mass index; DSA, deletion–substitution–addition; IQR, interquartile range; OR, odds ratio; SD, standard deviation.

^a^ McFadden's pseudo *R* ^2^ was calculated for explained variance in the odds of being overweight and obesity at the age of 10 years.

**FIGURE 3:**
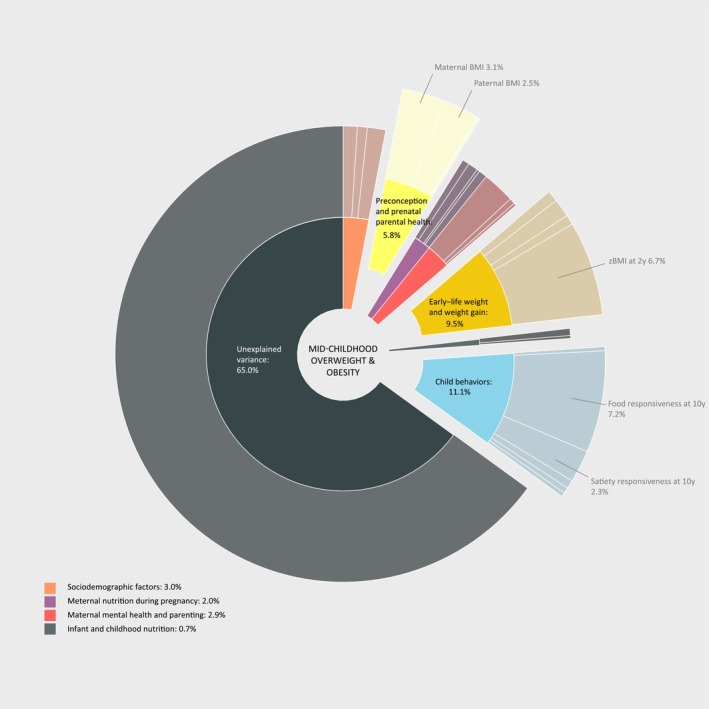
Explained variance in childhood overweight and obesity at age 10 years. This figure illustrates the relative explained variance by 25 key predictors selected for overweight and obesity at age 10 years. Each segment of the outer level of the pie chart corresponds to predictors of overweight and obseity listed in sequential order as presented in Table 3. The key predictors combined explained 33.7% variance in the odds of having overweight or obesity at age 10 years. The decomposition of *R* ^2^ shows that key predictors in the preconception and prenatal parental health (5.8%), early‐life weight and weight gain (9.5%), and child behavior (11.1%) domains constituted the largest proportions. Within these domains, maternal prepregnancy BMI, paternal BMI, child BMI at age 2, food responsiveness, and satiety responsiveness at age 10 accounted for substantial proportions.

The final multi‐predictor DSA models of overweight and obesity selected 25 key predictors (Table 3; overweight or obesity). Among them, 20 predictors (~80%) overlap with those selected from DSA models of *z*BMI. In addition to these overlapping predictors, the models selected maternal total n‐6 PUFA status during pregnancy, the timing of the introduction of solid foods, breastfeeding duration, emotional overeating at age 10 years, and outdoor play. The key predictors combined explained 33.7% variance in the odds of having overweight or obesity at age 10 years. Figure 3 illustrates the relative explained variance by each predictor of childhood overweight and obesity, with predictors in the preconception and prenatal parental health (5.8%), early‐life weight and weight gain (9.5%), and child behavior (11.1%) domains constituting the largest proportions. Within these domains, maternal prepregnancy BMI, paternal BMI, child BMI at age 2, food responsiveness, and satiety responsiveness at age 10 accounted for substantial proportions.

Adding child BMI PRS to the main models does not substantially change effect estimates or *R* ^2^ (Table S15): The explained variance in *z*BMI is 48.7% (95% CI: 46.1%, 51.2%) when adding the child BMI PRS and additionally adjusted for four genetic ancestry principal components in the model. In the model of overweight and obesity, the explained variance slightly increased to 35.2% when additionally adding child BMI PRS. Effect estimates were similar when stratified by child sex (Table S16) and ethnic backgrounds (Table S17). After examining the cross‐validation risk plots for child *z*BMI (Figure S2A) and weight status (Figure S2B), we determined an optimal maximum model size of 10 predictors for each model and conducted multiple regression analyses in a parsimonious way. The 10 selected key predictors (Table S18) align with those that accounted for the largest proportions in the main models. Although comparisons between these parsimonious models and the main models indicate that the main models are preferred (*p* < 0.001), the explained variance by 10 predictors in the parsimonious models (46.4% for *z*BMI and 31.3% for overweight and obesity) suggests a comparable fit to the main models. Finally, in the models with 59 candidate predictors, the overall explained variance is 42.9% (95% CI: 40.3%, 45.6%) for child BMI and 35.5% for the odds of overweight and obesity (data not shown).

## 4 Discussion

This study used systematic analytical strategies to synthesize and analyze predictors of BMI in mid‐childhood, spanning preconception parental health through subsequent fetal and child development, with the goal of identifying salient predictors. Out of 210 predictors examined by studies conducted using the Generation R Study data, we identified 59 candidate predictors, from which 32 were identified as independent key predictors of either child *z*BMI or weight status at age 10 years by the DSA model search algorithm. These key predictors included child's ethnic background and parental educational levels in the *sociodemographic* domain; parental BMI before pregnancy and mother's smoking status during pregnancy in the *preconception and prenatal parental health* domain; mother's total n‐6 PUFA status, blood concentration of vitamin D, folic acid supplement use, and milk consumption in the *maternal nutrition during pregnancy* domain; the timing of the introduction of solid foods, breastfeeding duration, and mother's regulation of child food intake in the *maternal mental health and parenting* domain; weight gain and BMI during infancy in the *early‐life weight* domain; adherence to a high fat mass dietary pattern and protein intake during infancy in the *infant and childhood nutrition* domain; and enjoyment of food, emotional overeating, food responsiveness, satiety responsiveness, breakfast skipping, television viewing, outdoor play, and higher set‐shifting abilities in the *child behavior* domain. In total, these identified key predictors accounted for approximately 49.2% of the variance in *z*BMI and 33.7% of the variance in the odds of having overweight or obesity at the age of 10 years.

### 4.1 Strongest Predictors in Prenatal, Early‐Life Weight, and Child Behavior Domains

To our knowledge, this is the first study to elucidate the contributions of a wide range of predictors simultaneously for childhood BMI. Our study contributes to prioritizing key predictors that explain the variability of child BMI by decomposing the explained variance. We pinpointed predictors across three domains—early‐life weight, child behaviors, and preconception and prenatal parental health—that accounted for the largest proportions of explained variance in BMI and weight status.

Within the early‐life weight domain, infant weight gain and BMI across infancy were the strongest predictors, accounting for the largest fraction of the variability in child *z*BMI at age 10 years, namely, 20.2%. Rapid weight gain in infancy has consistently been linked to risk of later childhood obesity and has been used in prediction models. One meta‐analysis reported that rapid weight gain during the first 2 years of life (defined as > 0.67 weight gain z‐score) was associated with 4.16 times greater odds of having overweight or obesity in childhood [38]. Our results on absolute weight gain at three time points during infancy are in line with this finding, although the effect estimates are not directly comparable. BMI at single time points during infancy, at age 2 years, and at estimated adiposity peak were also identified as key predictors, in accordance with previous studies reporting strong tracking of early‐childhood BMI into adolescence [3]. A study comparing BMI and infant weight gain suggested a simple measure of weight status at a single time point between 6 and 24 months of age to be a stronger risk factor for later obesity and may have more clinical utility and accuracy than weight gain [39]. In a study tracking the course of BMI over time in over 50,000 children in Germany, adolescents with overweight and obesity had higher mean BMI z‐scores starting at age 1 year and had the greatest annual increase in BMI z‐score between ages 2 and 6 years [3], which was suggested to be a feature of early adiposity rebound [40]. Considering that early adiposity rebound can only be observed retrospectively, a higher BMI at age 2 years may be a more feasible indicator for future upward deviation in BMI percentile and a useful measure for population‐based prevention of obesity.

Within the child behavior domain, food responsiveness and satiety responsiveness emerged as the strongest predictors, collectively constituting the largest fraction of the variance of the odds of having overweight and obesity at the age of 10 years (9.5%) and the second largest fraction of the variability in child *z*BMI at age 10 years (10.2%). Notably, to our knowledge, these predictors have not been previously integrated into prediction models, despite numerous observational studies reporting consistent associations with BMI in childhood. In a recent meta‐analysis, pooled effect estimates of food responsiveness on *z*BMI are comparable to our finding, whereas our effect estimate of satiety responsiveness is somewhat smaller [41]. One explanation could be that we included early‐life weight gain in the model, which has been found to predict decreased satiety responsiveness at age 5 years [42] but is not commonly controlled in previous studies. Longitudinal assessments of these eating behaviors revealed a moderate stability of both food responsiveness and satiety responsiveness in children aged 2–11 years [43, 44, 45]. Moreover, bidirectional associations between these eating behaviors and BMI have also been observed in children older than 4 years of age in a few longitudinal studies [41]. Considering that food responsiveness and satiety responsiveness measured at age 10 years were selected in the model, we reckon that they may be (partly) the consequence of higher BMI prior to mid‐childhood.

Within the preconception and prenatal parental health domain, parental BMI before pregnancy was a critical predictor that stands as the third largest part of the variability in mid‐childhood *z*BMI. Parental BMI is the most used predictor in prediction modeling and appears to be an important predictor in studies examining obesity in mid‐childhood [46]. Familial aggregation of obesity is well documented; parents provide both genes and obesogenic family environment that can promote childhood obesity in susceptible individuals. Recent meta‐analyses quantified the effect sizes of these relationships. For example, an individual participant data meta‐analysis using data from 162,129 mother–child pairs across 37 cohort studies, including the Generation R Study, underscored the impact of maternal prepregnancy BMI on child BMI, particularly in late childhood (10–18 years) [47]. Another meta‐analysis highlighted a positive association in children aged between 1 and 14 years, reporting a 264% increase in the odds of child obesity when mothers have obesity before conception [48]. Paternal prepregnancy BMI, although less studied, also showed considerable associations with offspring BMI, possibly with a greater impact from mid‐childhood onwards [49]. Two reviews compared the magnitude of mother–child and father–child BMI associations [50, 51]. Together, these studies suggest that there is no consistent pattern until mid‐adolescence, but thereafter, the magnitude of associations of parental BMI with offspring BMI were similar for both parents (around 0.23 per SD increase in maternal or paternal BMI). Our results are in line with these estimated magnitudes. Collectively, these findings emphasize an equal importance between maternal and paternal (prepregnancy) BMI in predicting offspring adiposity measures from mid‐childhood onwards.

### 4.2 Explained Proportions of the Variability in Mid‐Childhood BMI and Weight Status

Our study reveals a remarkable proportion of explained variance in mid‐childhood *z*BMI and weight status: With the large number of predictors studied, approximately half of the variability in BMI was explained. This finding underscores the multifaceted nature of obesity in childhood, which has been widely acknowledged as having both gene–environment causes, which may also interact.

Notably, the strongest predictors found in this study appeared to be precursing or mediating phenotypes that have some genetic underpinnings and may reflect such complex interplay. In light of this, our identified proportion of the explained variance warrants interpretation within the context of the heritability of child BMI. Narrow‐sense heritability (*h* ^2^) has been used to quantify the proportion of variance attributable to the additive genetic variation, which is equivalent to the *R* ^2^ in a linear regression model in which additive genetic variation is regressed on BMI. A study including 45 twin studies found that the *h* ^2^ of BMI increased with age, ranging from 42% to 84%, and the *h* ^2^ reached its highest estimate (around 80%) for both boys and girls around mid‐childhood [52]. Most, if not all, of the individual‐level key predictors selected in this study are heritable. For example, estimated *h* ^2^ for food responsiveness and satiety responsiveness were around 70% [53, 54]. Moreover, family‐level key predictors, including parental BMI, can have a strong influence through inheritance. In this study, we included PRS of child BMI in the models, which captures part of the additive genetic variation. However, the inclusion of BMI PRS did not substantially change the explained variance in child BMI or weight status. This is not surprising, considering that as observed in a previous study [55], the 25‐loci combined PRS accounted for merely 3.6% of the variability in childhood BMI at 10 years. These findings align with the observation that the combination of common variants typically only explain small fractions of trait variance [56]. For example, a recent PRS study based on 2.1 million common SNPs explained only 8.4% of the variation in BMI [57]. Moreover, because we included predictors that are presumably on the pathways of genes to BMI, the effect of PRS was further diluted. On the other hand, this suggests that we may already capture a considerable part of the genetic variation with phenotypical predictors.

The significant role of genetic factors in BMI does not downplay the importance of environmental factors associated with childhood obesity, because genetic factors can affect BMI by modifying food intake and behavioral factors. Importantly, shared and unique environmental factors explain at least 20% of the variability of BMI throughout childhood [52]. Our estimated explained variance of BMI likely encompasses a substantial part of both genetic variance, environmental variance, and their covariance. However, a notable missing proportion of explained variance of mid‐childhood BMI remains. Factors from psychosocial, cultural, broader environmental, and macro‐system domains not addressed in this study, such as childhood adverse experiences [58], cultural beliefs [59], food environment [60], food marketing [61], air pollution [62], built environment [63], early child care environment [64], and utilization of perinatal services [65], have all been associated with BMI trajectories and the development of obesity in childhood in other work but were not (yet) addressed in the Generation R Study. For genetic variance, missing heritability suggests that additional genetic loci (including low/rare frequency alleles) or other genetic variants such as copy number variations predisposing to BMI and overweight or obesity remain to be discovered. Epigenetic mechanisms may be another source of missing variance, as it has shown additive explanatory capacity to health outcomes beyond genetic predictors [66] and have partially captured environmental effect [67]. Moreover, integrated multi‐omics approaches, which include downstream physiological response to the interaction between genes and environment, also hold the potential to fill in a fraction of the missing proportions of the variability in mid‐childhood BMI. Considering that understanding how the environment interacts with genetic predisposition to obesity is challenging to achieve soon, future studies should also focus on investigating modifiable causal factors. The key predictors identified in the current study have potential for substantial effects; however, randomized controlled trials are needed to establish causality and modifiability in practice.

### 4.3 Strengths and Limitations

Major strengths of this study are the utilization of domain‐related knowledge generated from a large pediatric population‐based prospective study and the inclusion of a wide range of predictors of childhood obesity, covering factors across preconception to mid‐childhood. We preselected predictors in an evidence‐based manner, taking prior knowledge into account before building prediction models. This approach would potentially improve the interpretability of prediction models. Furthermore, we used a model search algorithm with cross‐validation to select the “best bet” list of important predictors. We identified novel predictors, such as eating behaviors, as key predictors that often not included in previous prediction modeling studies. Moreover, we identified the proportion of variability in mid‐childhood BMI that can and cannot be explained.

Our results should be interpreted in the context of several limitations. First, the study was conducted in one cohort study in the Netherlands; thus, the results are population‐specific and prone to overfitting. The generalizability of the findings needs to be established. Predictors selected by meta‐analysis but not by DSA could still be important to predict child BMI in other settings. Therefore, replication is needed in other cohorts with data collected from a life course perspective. Second, limitations associated with using zBMI scores should be acknowledged. The reference population for the Dutch growth chart included only children with at least one parent of Dutch origin. Consequently, applying this chart to standardize BMI in children from non‐Dutch or mixed backgrounds could introduce bias. However, sensitivity analyses stratified by ethnic background showed similar estimates for children with Dutch and non‐Dutch backgrounds, supporting the robustness of the key predictors identified in this study. Additionally, zBMI shows a reduced ability to distinguish among children with severe obesity due to the “ceiling effect.” This effect arises from restricted zBMI variance above the 97th percentile due to sparse reference data, which was observed in other growth references using the LMS method, such as the 2000 CDC growth chart [68]. This limitation may influence the generalizability of our findings to children at the more extreme end of the BMI spectrum. Third, interactions between two or more variables may account for additional explained variance of BMI or weight status, which were not examined in this study, as potential synergistic effects among predictors may have a stronger influence on BMI or weight status than their combined additive effect. Indeed, a few previous studies included in our evidence synthesis reported interactions between some predictors and sex or child ethnic background [69, 70, 71, 72]. However, due to constrains of statistical power, we did not formally examine these interactions in the main models and instead presented stratified results as sensitivity analysis. Fourth, we cannot infer causality from the results because of the exploratory and correlational nature of our prediction model. Fifth, we cannot rule out the potential contribution of predictors that were not selected by the DSA algorithm in the variability of mid‐childhood BMI, despite that they may have relatively small effect sizes and may not be independently associated with BMI in mid‐childhood in the current study. Those predictors might still be of interest in terms of prevention and intervention because they may be on the pathway of selected predictors to child BMI, for example, as precursors, mediators, or effect modifiers.

Some conceptionally important predictors were not selected, for example, diet in childhood. Nevertheless, our results do not downplay those predictors because they are associated with the identified key predictors in the current study to a varying degree. Instead, future studies may take the key predictors we identified as a starting point to investigate the underlying causal mechanism of childhood obesity in experimental studies. Last, data on some biological factors and environmental factor (i.e., immune biomarkers, taste preference, sleep, 24‐h activity rhythms, air pollution, and endocrine‐disrupting chemicals) was only available in sub‐cohorts [73, 74, 75, 76, 77, 78], therefore not included in this study. Data on macro‐system‐level factors (e.g., policy‐related predictors) were not reported in the published studies of Generation R. Those potential predictors need attention in large‐scale studies across countries in the future.

## 5 Conclusion

We found that mid‐childhood BMI and weight status were associated with multiple predictors in multifaceted domains, particularly those related to preconception and prenatal parental health, early‐life weight, and child behaviors. We observed key predictors spanning various aspects of children's development explain a larger proportion of the variance in BMI and weight status. Nevertheless, the large gap of unexplained variance suggests that some uncharted predictors need to be uncovered or that “main effects are in the interactions” [79]. Our results set a stage for future research to understand how those predictors jointly mediate the influence of the obesogenic environment on child BMI. Although more experimental intervention studies are needed to establish causality, we speculate that multimodal interventions targeting the early life cycle are potentially the most effective in maintaining a healthy weight along the life course. Our sensitivity analysis, using a parsimonious model with 10 key predictors primarily clustered in the three domains mentioned above, demonstrated a comparable fit to our main model. Future predictive and interventional studies are encouraged to test and validate these 10 key predictors for utilization in practical settings. Likely, such interventions target factors that affect parental BMI in the pre‐conception phase and during pregnancy as well as managing appetite and preventing early adiposity rebound are the most impactful. Early life preventive efforts, starting as early as preconception stages, are crucial for supporting parents and children in adopting healthy behaviors and maintaining a healthy weight. Recent developed prevention programs for adults planning to have children have shown promising effects with the adoption of positive health behaviors and weight management [80]. Whether the reported effects on adults' lifestyle and weight also translate to offspring's health behaviors that carry over into later life by reducing obesity risk needs to be shown in the future.

## Author Contributions

**Yuchan Mou:** conceptualization, data screening, data extraction, data curation, statistical analysis, writing – original draft, writing – review and editing. **Susana Santos:** consultation of statistical analysis, writing – review and editing. **Macarena Lara:** data screening, data extraction, writing – review and editing. **Ivonne P. M. Derks:** data extraction, writing – review and editing. **Vincent W. V. Jaddoe:** resources, writing – review and editing. **Romy Gaillard:** resources, writing – review and editing. **Janine F. Felix:** resources, writing – review and editing. **Eric Steegers:** resources, writing – review and editing. **Trudy Voortman:** data screening, data extraction, resources, writing – review and editing. **Marinus H. van IJzendoorn:** conceptualization, data screening, data extraction, consultation of statistical analysis, writing – review and editing. **Pauline W. Jansen:** conceptualization, resources, data screening, data extraction, supervision, writing – review and editing. Yuchan Mou and Pauline W. Jansen had primary responsibility for final content. All authors read and approved the final manuscript.

## Conflicts of Interest

The authors declare no conflicts of interest.

## Supporting information


**Table S1.** Statistics conversion methods.
**Table S2.** Effect sizes computation and conversion to Fisher’s z methods.
**Table S3.** Percentages of missing values in each predictor and variables used for multiple imputation.
**Table S4.** Overview of included papers.
**Table S5.** Pooled effect sizes of predictors in sociodemographics factors domain.
**Table S6.** Pooled effect sizes of predictors in preconception and prenatal parental health domain.
**Table S7.** Pooled effect sizes of predictors in maternal nutrition during pregnancy domain.
**Table S8.** Pooled effect sizes of predictors in genetics domain.
**Table S9.** Pooled effect sizes of predictors in maternal mental health and parenting domain.
**Table S10.** Pooled effect sizes of predictors in early‐life weight and weight gain domain.
**Table S11.** Pooled effect sizes of predictors in infant and childhood nutrition domain.
**Table S12.** Pooled effect sizes of predictors in child behavior domain.
**Table S13.** Pooled effect sizes of predictors in biological factor domain.
**Table S14.** Descriptive statistics for all predictors included in multiple regression models (*n* = 5686).
**Table S15.** Associations between key predictors and zBMI or overweight and obesity in multiple regression models including child BMI polygenic risk score (*N* = 3584).
**Table S16.** Associations between key predictors and zBMI or overweight and obesity in multiple regression models stratified by child sex (*N* = 5686).
**Table S17.** Associations between key predictors and zBMI or overweight and obesity in multiple regression models stratified by child ethnic background (*N* = 5686).
**Table S18.** Associations between predictors and zBMI or overweight and obesity at age 10 years in parsimonious multiple regression models (*N* = 5686).
**Figure S1.** Forest plots for predictors of child BMI with pooled effect sizes above the threshold (|d| ≥ 0.1).
**Figure S2.** Cross‐validation plots based on DSA‐selected models for (A) BMI and (B) overweight and obesity in children aged 10 years.
**Supplementary Methods S1.** Search strategy.
**Supplementary Methods S2.** Assessment of predictors.
**Supplementary Methods S3.** Multiple imputation.

## Data Availability

Data described in the predictive modeling part cannot be made publicly available because of confidentiality, but data can be requested and shared with a formal data‐sharing agreement. Requests for data, code book, and analytic code can be directed to datamangementgenr@erasmusmc.nl.
