# Supplementary material for: Identifying Key Predictors of Mid‐Childhood Obesity in a Population‐Based Cohort Study: An Evidence Synthesis and Predictive Modeling Study
Source: Obes Rev. 2025 Jun 12;26(11):e13958. doi: 10.1111/obr.13958 (PMC12531732; doi:10.1111/obr.13958)
Supplement: Supplementary file 1 — Table S1. Statistics conversion methods. Table S2. Effect sizes computation and conversion to Fisher’s z methods. Table S3. Percentages of missing values in each predictor and variables used for multiple imputation. Table S4. Overview of included papers. Table S5. Pooled effect sizes of predictors in sociodemographics factors domain. Table S6. Pooled effect sizes of predictors in preconception and prenatal parental health domain. Table S7. Pooled effect sizes of predictors in maternal nutrition during pregnancy domain. Table S8. Pooled effect sizes of predictors in genetics domain. Table S9. Pooled effect sizes of predictors in maternal mental health and parenting domain. Table S10. Pooled effect sizes of predictors in early‐life weight and weight gain domain. Table S11. Pooled effect sizes of predictors in infant and childhood nutrition domain. Table S12. Pooled effect sizes of predictors in child behavior domain. Table S13. Pooled effect sizes of predictors in biological factor domain. Table S14. Descriptive statistics for all predictors included in multiple regression models (n = 5686). Table S15. Associations between key predictors and zBMI or overweight and obesity in multiple regression models including child BMI polygenic risk score (N = 3584). Table S16. Associations between key predictors and zBMI or overweight and obesity in multiple regression models stratified by child sex (N = 5686). Table S17. Associations between key predictors and zBMI or overweight and obesity in multiple regression models stratified by child ethnic background (N = 5686). Table S18. Associations between predictors and zBMI or overweight and obesity at age 10 years in parsimonious multiple regression models (N = 5686). Figure S1. Forest plots for predictors of child BMI with pooled effect sizes above the threshold (|d| ≥ 0.1). Figure S2. Cross‐validation plots based on DSA‐selected models for (A) BMI and (B) overweight and obesity in children aged 10 years. Supplementary Methods S1. [file OBR-26-e13958-s001.pdf]

Identifying key predictors of mid-childhood obesity in a population-based cohort study: An evidence synthesis and predictive modeling study

Yuchan Mou[1, 2], Susana Santos[2, 3, 4], Macarena Lara[1], Ivonne P.M. Derks[5, 6, 7], Vincent W.V. Jaddoe[1, 2, 3], Romy Gaillard[1, 2, 3], Janine F. Felix[2, 3], Eric Steegers[8], Trudy Voortman[1,9], Marinus H. van IJzendoorn[10, 11], Pauline W. Jansen[5, 6]

Affiliations

1. Department of Epidemiology, Erasmus MC, University Medical Center Rotterdam, The Netherlands

2. The Generation R Study Group, Erasmus MC, University Medical Center Rotterdam, The Netherlands

3. Department of Pediatrics, Erasmus MC, University, Medical Center Rotterdam, The Netherlands

4. EPIUnit ITR, Instituto de Saúde Pública da Universidade do Porto, Universidade do Porto, Rua das Taipas, n° 135, 4050-600 Porto, Portugal

5. Department of Psychology, Education and Child Studies, Erasmus University Rotterdam, The Netherlands

6. Department of Child and Adolescent Psychiatry / Psychology, Erasmus MC, University Medical Center Rotterdam, The Netherlands

7. Research Department of Behavioural Science and Health, University College London, United Kingdom

8. Department of Obstetrics and Gynaecology, Erasmus MC, University Medical Center Rotterdam, Netherlands

9. Meta-Research Innovation Center at Stanford (METRICS), Stanford University, Stanford, CA, USA

10. Department of Psychiatry, Monash University, Melbourne, Australia

11. Research Department of Clinical, Education and Health Psychology, UCL, University of London, UK

Corresponding author: Pauline W. Jansen, Dr. Molewaterplein 40, PO Box 2040, 3000 CA, Rotterdam, the Netherlands, p.w.jansen@erasmusmc.nl

Table of Content

Supplementary Methods 1. Search strategy
Supplementary Methods 2. Assessment of predictors
Supplementary Methods 3. Multiple imputation
Table S1. Statistics conversion methods
Table S2. Effect sizes computation and conversion to Fisher's z methods
Table S3. Percentages of missing values in each perdictor and variables used for multiple imputation
Table S4. Overview of included papers
Table S5. Pooled effect sizes of predictors in sociodemographics factors domain
Table S6. Pooled effect sizes of predictors in preconception and prenatal parental health domain
Table S7. Pooled effect sizes of predictors in maternal nutrition during pregnancy domain
Table S8. Pooled effect sizes of predictors in genetics domain
Table S9. Pooled effect sizes of predictors in maternal mental health and parenting domain
Table S10. Pooled effect sizes of predictors in early-life weight and weight gain domain
Table S11. Pooled effect sizes of predictors in infant and childhood nutrition domain
Table S12. Pooled effect sizes of predictors in child behaviors domain
Table S13. Pooled effect sizes of predictors in biological factors domain
Table S14. Descriptive statistics for all predictors included in multiple regression models (n = 5686)
Table S15. Associations between key predictors and zBMI or overweight and obesity in multiple regression models including child BMI polygenic risk score (N = 3584)
Table S16. Associations between key predictors and zBMI or overweight and obesity in multiple regression models stratified by child sex (N = 5686)
Table S17. Associations between key predictors and zBMI or overweight and obesity in multiple regression models stratified by child ethnic background (N = 5686)
Table S18. Associations between predictors and zBMI or overweight and obesity at age 10 years in parsimonious multiple regression models (N = 5686)
Reference list of publications included in the evidence synthesis
Figure S1. Forest plots for predictors of child BMI with pooled effect sizes above the threshold ($|d| \geq 0.1$)
Figure S2. Cross-validation plots based on DSA-selected models for (A) BMI and (B) overweight and obesity in children aged 10 years

Supplementary Methods 1. Search strategy

Pubmed
(Drooger JC[Author] AND (Moll, Ha[Full Author Name] OR Moll HA[Author] OR Moll HA[Investigator])) OR (Rours GI[Author] AND (pregnant[Title] OR (pregnan*[Title] AND cohort[Title]))) OR (Gabriele C[Author] AND (Moll, Ha[Full Author Name] OR Moll HA[Author] OR Moll HA[Investigator])) OR (Verburg BO[Author] AND Jaddoe VW[Author]) OR ((“family characteristics”[MeSH Terms] OR (“family”[All Fields] AND “characteristics”[All Fields]) OR “family characteristics”[All Fields] OR “generation”[All Fields]) AND Jaddoe V[Author]) OR (Verkaik NJ[Author] AND Jaddoe VW[Author]) OR (van den Berg MP[Author] AND Jaddoe VW[Author]) OR (Witteman JC[Author] AND Jaddoe VW[Author] AND Hypotheses[All fields] OR Jaddoe VW[Author] AND Mook-Kanamori DO[Author])

Embase
embase is not capital sensitive (‘Drooger J’:au AND ‘Moll H’:au) OR (‘Rours G’:au AND (pregnant:ti OR (pregnan AND cohort):ti)) OR (‘Gabriele C’:au AND ‘Moll H’:au) OR (‘Verburg B’:au AND ‘Jaddoe V’:au) OR (‘Jaddoe V’:au AND generation) OR (‘Verkaik N’:au AND ‘Jaddoe V’:au) OR (‘van den Berg M’:au AND ‘Jaddoe V’:au) OR (‘Witteman J’:au AND ‘Jaddoe V’:au AND ‘Hypotheses’ OR ‘Jaddoe V’:au AND ‘Mook-Kanamori D’:au)

OV Medline
(Drooger J.au AND Moll H.au) OR (Rours G.au AND (pregnant OR (pregnan AND cohort)) .ti) OR (Gabriele C.au AND Moll H.au) OR (Verburg B.au AND Jaddoe V.au) OR ((“generation r”.ti,ab OR Jaddoe V.au) AND Rotterdam.in) OR (Verkaik N.au AND Jaddoe V.au) OR (van den Berg M.au AND Jaddoe V.au) OR (Witteman J.au AND Jaddoe V.au AND Hypotheses OR Jaddoe V.au AND Mook-Kanamori D*.au)

Web of Science ((AU=Drooger J* AND AU=Moll H) OR (AU=Rours G AND (TI=“pregnant” OR TI=(“pregnan” AND ”cohort”))) OR (AU=Gabriele C AND AU=Moll H) OR (AU=Verburg B AND AU=Jaddoe V) OR ((TS=(”generation R”) OR AU=jaddoe v) AND (AD=rotterdam)) OR (AU=Verkaik N AND AU=Jaddoe V) OR (AU=van den Berg M AND AU=Jaddoe V) OR (AU=Witteman J AND AU=Jaddoe V* AND TS=”Hypotheses” OR AU=Jaddoe V* AND AU=Mook-Kanamori D*))

Supplementary methods 2

Sociodemographic factors

Child ethnic background was defined by the country of birth of the parents, as obtained by questionnaires[1] and grouped children into Dutch, Non-Dutch Western and non-Dutch Non-Western ethnic origin. Information about the parents' education was obtained by questionnaire during pregnancy and when the children were 6 years old. We categorized the educational level into three groups: (1) low (no education, primary school, lower vocational training, intermediate general school, or ≤3 years general secondary school); (2) middle (>3 years general secondary school, intermediate vocational training); and (3) high (higher vocational training, university degree).

Preconception and prenatal parental health

Maternal pre-pregnancy weight was obtained by self-report questionnaires. Maternal and paternal weight and height were measured without shoes and heavy clothing at enrolment[2] and BMI was calculated as weight/height^2 (kg/m^2). Information about maternal smoking during pregnancy was assessed by questionnaires in the first, second and third trimester of pregnancy[3]. We categorized mothers' smoking status into three groups: (1) never smoked during pregnancy; (2) smoked until their pregnancy was known; (3) continued to smoke during pregnancy. Paternal smoking was reported by mothers on whether father smoked cigarettes, cigars or shag in early pregnancy (yes, no or do not know).

Maternal and paternal information on cannabis use was collected by a mother-reported questionnaire in early pregnancy. We grouped maternal cannabis use into four categories: (1) never used cannabis; (2) used cannabis before pregnancy; (3) used cannabis until their pregnancy was known; (4) continued cannabis use during pregnancy. Paternal cannabis use was dichotomized (never vs ever used).

Maternal nutrition during pregnancy

Non-fasting venous blood sample were collected in the second trimester of pregnancy at a median gestational age of 20.5 weeks (95% range 16.5 – 24.9). Total and individual n-6 Polysaturated fatty acids (PUFA) concentrations were expressed as the proportion of total fatty acids that were present in the chromatogram (weight percentage), including linoleic acid (LA; 18:2n–6), gamma-linolenic acid (GLA; 18:3n–6), eicosadienoic acid (EDA; 20:2n–6), dihomo-gamma-linolenic acid (DGLA; 20:3n–6), arachidonic acid (AA; 20:4n–6), and docosatetraenoic acid (DTA; 22:4n–6)[4]. Plasma 25-hydroxyvitamin D_2 (25(OH)D_2) and 25(OH)D_3 were quantified using isotope dilution liquid chromatography-tandem mass spectrometry and summed to Total 25(OH)D[5]. Periconceptional folic acid supplement use was assessed in the first trimester by questionnaire. We categorized mothers into not using supplementation, started in the first trimester (before 10 weeks), and started preconceptionally[6]. Information about maternal vomiting and nausea was obtained by questionnaires at enrollment in early pregnancy and was categorized into: (1) no daily vomiting versus (2) daily vomiting during early pregnancy.

Maternal milk intake during early pregnancy (median: 13.5 weeks of gestation, 95% range 10.8, 21.1) was assessed by a semi-quantitative food-frequency questionnaire (FFQ) consisting of 293 food items. We summed the consumption of milk and milk drinks (e.g., chocolate milk or other flavored, sweetened milk drinks) to obtain frequency measures of milk consumption. According to the

Dutch household measures, 1 glass of milk contains 150 milliliter milk on average[7]. We categorized maternal milk intake into 4 groups: <1, 1-<2, 2-<3, and ≥ 3 glasses per day.

Genetic variants

A child BMI polygenic risk score (PGS) was constructed using identified genetic loci associated with childhood BMI from a large consortium study, in which the Generation R Study was involved[8]. Briefly, 25 loci that achieved genome-wide significance were combined into a polygenic risk score by summing up the weighted number of risk alleles that are associated with an increase in BMI standardized deviation score (SDS). The weights accounted for genetic variant-specific effect sizes and were derived from the meta-analysis in the consortium study. The PGS ranges from 0 to 50. In this study population, genetic data was available for 3584 children whose height and weight were measured at the age of 10 years.

Maternal mental health and parenting

Information on maternal psychopathology symptoms was collected by the validated, self-reported Brief Symptom Inventory (BSI) [9], assessing symptoms of anxiety, depression, hostility, interpersonal sensitivity, obsessive-compulsive disorder, psychoticism, paranoia, phobia, and somatization. Items are reported on 5-point Likert scale. At 20 weeks of gestation, the full 53-item BSI was administered, from which we calculated a Global Severity Index (GSI) by summing all items, indicating the overall psychopathology symptoms.

Postpartum depression symptom was calculated by summing the items of the depression scale measured at 20 weeks, 2 months and 6 months.

Information on the age at introduction of the first solid foods and duration of exclusive breastfeeding was obtained from postnatal questionnaires at 2, 6, and 12 months after birth. Feeding practices were assessed with the Child Feeding Questionnaire (CFQ) when children were 4 and 10 years of age, including pressure to eat (attempting to improve the quantity or quality of children's food intakes;) and restriction (regulating the type and amount of food eaten by children) as subscales[10]. The items were answered on a 5-point Likert Scale and summed per subscale.

Early-life weight and weight gain

Infant weight and length were measured in community health centers at ages 6, 12, and 24 months. Weight was measured with a mechanical personal scale. Length was measured in a supine position to the nearest millimeter with a neonatometer. BMI was calculated as kg/m^2. Age- and sex- adjusted SDS of weight, length and BMI were obtained using Dutch reference growth charts (Growth Analyzer 3.5) [11]. Weight gain and BMI-SDS gain were quantified as the difference between assessment time points. BMI at estimated adiposity peak in infancy was obtained using a cubic mixed effect model fitted on log(BMI) from 14 days to 1.5 years, using sex as a covariate. BMI was derived for each child at the point where the curve reached its maximum[12].

Infant and childhood nutrition

Dietary intake was assessed at the age of 1 year with a semi-quantitative FFQ consisting of 211 food items. Food frequencies were converted into energy and macronutrient intake based on the standardized portion sizes and the Dutch Food Composition Table 2006[13]. Protein intake was divided into protein from animal food sources (e.g., dairy) and from plant-based food sources (e.g., grains).

The *a posteriori* dietary patterns used in the study were extracted by reduced rank regression[14], which explained the maximal variance in fat mass index and fat-free mass index. The dietary pattern derived from fat-mass and fat-free mass index was characterized by high intake of refined grains, meat, potatoes, fish, soups and sauces, and sugar-containing beverages.

Child behavior

Child eating behaviors were assessed using the Children's Eating Behavior Questionnaire (CEBQ) at ages 4 and 10 years, including the subscales emotional undereating, emotional overeating, enjoyment of food, food responsiveness, and satiety responsiveness[15]. Parents rated their children's eating behavior tendencies on a 5-point Likert Scale. Children's consumption of breakfast, lunch, and dinner were assessed by single-item questions reported on by parents when children were 4 and 6 years old[16]. At 4 years, the Brief Rating Inventory of Executive Function-Preschool Version[17] was administered to measure functions including set-shifting ability (i.e. cognitive flexibility, which is the ability to move back and forth between tasks, operations or mental sets) with three response options (0 = never to 2 = very often/aways). Sedentary and physical activity behaviors were assessed by parent-reported questionnaire when the child was 6 years old. In this study, sedentary behaviors included television viewing (including video/DVD) and computer/video game use; physical activity included outdoor play. For all variables, the frequency and duration were asked for weekdays and weekend days separately. We

categorized average television viewing into ≥2 h/day and <2 h/day, and computer game use into ≥1 h/day and <1 h/day. Outdoor play was dichotomized into <1 hour/day versus ≥1 hour/day[18].

Other biological factors

Hair glucocorticoid was measured using hair samples of around 100-200 strands that were cut from the posterior vertex close to the scalp. Cortisol and cortisone were measured simultaneously with a liquid chromatography tandem mass spectrometry (LC-MS/MS) assay. Briefly, the proximal 3 cm of hair samples were weighed using an electrical scale and minced. Hair samples were then washed in LC-grade isopropanol for 2 min at room temperature, and left to dry for at least 2 days. Deuterium-labeled cortisol and cortisone were added before extraction. Extraction was performed using LC-grade methanol (MeOH), for 18 h at 25 °C in the presence of deuterated steroids. Subsequently, the extract was cleaned using solid phase extraction and steroids were quantified on a Xevo TQS LC–MS/MS (Waters Corporation, Milford, MA, USA) [19].

Supplementary Methods 3

Multiple imputation

Missing values of candidate predictors, except child BMI PRS, were imputed using multiple imputation under the assumption of missingness at random. Potential variables used to impute missing values of another variable were restricted to those that had: 1) an absolute correlation greater than 0.1 with the variable or with the binary missing variable indicating if the variable is missing; and 2) a proportion of missing observations less than 50%. These restrictions ensured that the number of variables used to impute was within the range of 5-25, as including no more than 25 variables is recommended in missing imputation model specification[20]. Continuous variables with a skewed distribution were entered in the imputation process with original scales, as Box-Cox power transformations to achieve normality were not sufficient. We forced child ethnicity, maternal education, and family income into the imputation model as these variables are related to the probability of missingness of variables. Twenty imputed data sets were generated and used in subsequent analyses.

After imputation, numeric and graphic diagnostics proposed for cohort studies were conducted to ensure the imputation was done properly[21]. Continuous variables were flagged if they had 1) an absolute difference between means of the observed and imputed values greater than 2 standard deviations; or 2) a ratio of variances of the observed and imputed values that is less than 0.5 or greater than 2. Categorical variables were flagged if the chi-squared test between observed and imputed values was significant. In the current study, no variables were flagged.

References

1 Gishti O, Kruithof CJ, Felix JF, et al. Ethnic disparities in general and abdominal adiposity at school age: a multiethnic population-based cohort study in the Netherlands. *Ann Nutr Metab*. 2014; 64: 208-17.

2 Jharap VV, Santos S, Steegers EAP, Jaddoe VWV, Gaillard R. Associations of maternal obesity and excessive weight gain during pregnancy with subcutaneous fat mass in infancy. *Early Hum Dev*. 2017; 108: 23-28.

3 Durmus B, Heppe DH, Taal HR, et al. Parental smoking during pregnancy and total and abdominal fat distribution in school-age children: the Generation R Study. *Int J Obes (Lond)*. 2014; 38: 966-72.

4 Vidakovic AJ, Gishti O, Voortman T, et al. Maternal plasma PUFA concentrations during pregnancy and childhood adiposity: the Generation R Study. *Am J Clin Nutr*. 2016; 103: 1017-25.

5 Miliku K, Felix JF, Voortman T, et al. Associations of maternal and fetal vitamin D status with childhood body composition and cardiovascular risk factors. *Matern Child Nutr*. 2018; 15: e12672.

6 Monasso GS, Santos S, Geurtsen ML, et al. Associations of Early Pregnancy and Neonatal Circulating Folate, Vitamin B-12, and Homocysteine Concentrations with Cardiometabolic Risk Factors in Children at 10 y of Age. *J Nutr*. 2021; 151: 1628-36.

7 Donders-Engels MR, van der Heijden L, Maten HK. Gewichten en Codenummers [Weights, measures and code numbers]. Wageningen (Netherlands): Vakgroep Humane Voeding, Landbouwuniversiteit Wageningen en TNO Voeding Zeist 2003.

8 Vogelezang S, Bradfield JP, Ahluwalia TS, et al. Novel loci for childhood body mass index and shared heritability with adult cardiometabolic traits. *PLOS Genetics*. 2020; 16: e1008718.

9 Derogatis LR, Melisaratos N. The Brief Symptom Inventory: an introductory report. *Psychological Medicine*. 1983; 13: 595-605.

10 Birch LL, Fisher JO, Grimm-Thomas K, et al. Confirmatory factor analysis of the Child Feeding Questionnaire: a measure of parental attitudes, beliefs and practices about child feeding and obesity proneness. *Appetite*. 2001; 36: 201-10.

11 Fredriks AM, van Buuren S, Wit JM, Verloove-Vanhorick SP. Body index measurements in 1996-7 compared with 1980. *Arch Dis Child*. 2000; 82: 107-12.

12 Mook-Kanamori DO, Durmuş B, Sovio U, et al. Fetal and infant growth and the risk of obesity during early childhood: the Generation R Study. *Eur J Endocrinol*. 2011; 165: 623-30.

13 Dutch food composition database 2006 (NEVO 2006). The Hague, The Netherlands: Netherlands Nutrition Center.

14 Voortman T, Leermakers ET, Franco OH, et al. A priori and a posteriori dietary patterns at the age of 1 year and body composition at the age of 6 years: the Generation R Study. *Eur J Epidemiol*. 2016; 31: 775-83.

15 Wardle J, Guthrie CA, Sanderson S, Rapoport L. Development of the Children's Eating Behaviour Questionnaire. *Journal of Child Psychology and Psychiatry*. 2001; 42: 963-70.

16 Mou Y, Jansen PW, Raat H, Nguyen AN, Voortman T. Associations of family feeding and mealtime practices with children's overall diet quality: Results from a prospective population-based cohort. *Appetite*. 2021; 160: 105083.

17 Gioia GA, Isquith PK, Retzlaff PD, Espy KA. Confirmatory factor analysis of the Behavior Rating Inventory of Executive Function (BRIEF) in a clinical sample. *Child Neuropsychol*. 2002; 8: 249-57.
18 Wijtzes AI, Bouthoorn SH, Jansen W, et al. Sedentary behaviors, physical activity behaviors, and body fat in 6-year-old children: the generation R study. *Int J Behav Nutr Phys Act*. 2014; 11: 96.
19 Noppe G, van den Akker EL, de Rijke YB, et al. Long-term glucocorticoid concentrations as a risk factor for childhood obesity and adverse body-fat distribution. *Int J Obes (Lond)*. 2016; 40: 1503-09.
20 van Buuren S, Groothuis-Oudshoorn K. mice: Multivariate Imputation by Chained Equations in R. *Journal of Statistical Software*. 2011; 45: 1 - 67.
21 Stuart EA, Azur M, Frangakis C, Leaf P. Multiple Imputation With Large Data Sets: A Case Study of the Children's Mental Health Initiative. *American Journal of Epidemiology*. 2009; 169: 1133-39.

Supplementary Tables

In this supplementary tables file, we presented the methods for converting different original statistics (median, mean, beta/B) into statistics (mean(sd) and β) used in the effect sizes computation in **Table S1**. **Table S2** presents the methods and formula of effect sizes computation and conversion into Fisher's z statistics which used in the meta-analysis. **Table S3** shows percentages of missing values in each predictor and variables used for imputation. **Table S4** presents the overview of characteristics of included papers. **Table S5-S13** show the pooled effect sizes of predictors within each domain. We used Cohen's d = 0.1 as the cut-off value. Values of effect sizes which were colored as red denote that effect size values larger than the cut-off, and values of effect sizes which were colored as blue denote that effect size values smaller than the cut-off. **Table S14-18** report the results from sensitivity analyses.

Table S1. Statistics conversion methods

Predictors Type	Original effect size statistics	After conversion	Method
categorical	Mean, 95% CI	Mean, SD	$SD = \frac{(Upperlimit-Lower)}{2*1.96*\sqrt{\frac{(n+1)}{n}}}$
categorical	Median, range or IQR	Mean, SD	Used *method 3 'the first quartile, median, the third quartile, sample size'* proposed by *X. Wan. et al, 2014*
categorical	Median, 90% or 95% range	Mean, SD	90% or 95% range proxy to range. Then used *method 1 'minimum, median, maximum, sample size'* proposed by *X. Wan et al, 2014*
Continuous	B	β	$\beta = B\frac{sd_x}{sd_y}$

[1] CI: confidence interval; SD: standard deviation; IQR: interquartile range

Table S2. Effect sizes computation and conversion to Fisher's z methods

Predictors Type	Information needed[4]	Methods[1, 3, 4]
Categorical	$\overline{x}_1, \overline{x}_2, sd_1, sd_2, n_1, n_2$	(1) We calculated SMD: $ES_{smd} = \frac{\overline{x}_1 - \overline{x}_2}{sd_{pooled}}$, $v_{smd} = \frac{n_1+n_2}{n_1*n_2} + \frac{ES^2_{smd}}{2*(n_1+n_2)}$, where $sd_{pooled} = \sqrt{\frac{sd_1^2*(n_1-1)+sd_2^2*(n_2-1)}{n_1+n_2-2}}$. (2) SMD was converted to r. $r = \frac{ES_{smd}}{\sqrt{ES_{smd}+\frac{1}{p*(1-p)}}}$, $v_r = \frac{v_{smd}}{v_{smd}+\frac{1}{p*(1-p)}}$. (3), r was converted to Fisher's z: $z_r = 0.5 * log(\frac{1+r}{1-r})$, $v_z = \frac{1}{n-3}$. Functions in R *'esc'* package[2]: *esc_mean_sd()* and *convert_d2r()*.
Categorical	$B/\beta, sd_y, n_1, n_2$	For B: (1) We calculated SMD: $ES_{smd} = \frac{B}{sd_{pooled}}$, $v_{smd} = \frac{n_1+n_2}{n_1*n_2} + \frac{ES^2_{smd}}{2*(n_1+n_2)}$, where $sd_{pooled} = \sqrt{\lvert\frac{sd_y^2*(n_1+n_2-1)-B^2*\frac{(n_1*n_2)}{n_1+n_2}}{n_1+n_2-2}}$. (2) SMD was converted to r: same as above. (3) r was converted to Fisher's z using the same formula as above. For β: $B = \beta * \frac{sd_y}{sd_x}$. Then the same formula as B was used. Functions in R *'esc'* package: *esc_B()* for B, *esc_beta()* for $beta$.

Predictors Type	Information needed[4]	Methods[1, 3, 4]
Categorical	B/β, exact p value, n_1, n_2	(1) We calculated the point-biserial correlation coefficient r_{pb}: $t = (p \text{ value }, k)$, $r_{pb} = \sqrt{\frac{t^2}{n_1+n_2-2+t^2}}$, where degree of freedom $k = n_1 + n_2 - 2$. (2) the SMD can be computed by: $ES_{smd} = \frac{r_{pb}}{\sqrt{p(1-p)(1-r_{pb}^2)}}$, $v_{smd} = \frac{n_1+n_2}{n_1*n_2} + \frac{ES_{smd}^2}{2*(n_1+n_2)}$, where $p = \frac{n_1}{n_1+n_2}$. (3) SMD converted to r: $r = \frac{ES_{smd}}{\sqrt{ES_{smd}+\frac{1}{p*(1-p)}}}$, $v_r = \frac{v_{smd}}{v_{smd}+\frac{1}{p*(1-p)}}$. (4) r converted to z use the same formula as above. Functions in R *'esc'* package: *esc_rpb()* and *convert_d2r()*.
Continuous	r, n_{total}	r was converted to Fisher's z using the same formula as above.
Continuous	β, n_{total}	We extracted β from unadjusted models (or considered as unadjusted effect sizes if there were no unadjusted effect sizes presented in the study), therefore β equals to r. r was converted to Fisher's z using the same formula as above.

[1] Lipsey MW, Wilson DB. Practical meta-analysis: SAGE publications, Inc 2001.

[2] Lüdecke D, Lüdecke MD, Calculator'from David BW. Package 'esc'. R Package Version 05. 2019; 1: 2019.

[3] $\overline{x}_1$, sd_1, n_1 is mean, standard deviation, number of comparison group, $\overline{x}_2$, sd_2, n_2 is mean, standard deviation, number of reference group. sd_y is standard deviation of BMI.

[4] sd: standard deviation; smd: standardized mean difference.

Table S3. Percentages of missing values in each perdictor and variables used for multiple imputation

Incomplete variables	Percentages of missing values (%)	Method used for imputation
Child sex	0.02	Logistic regression
Maternal age	0.02	Predictive mean matching
Child ethnic background	2.34	Polytomous logistic regression
Parity	3.18	Polytomous logistic regression
Maternal education - baseline	8.02	Polytomous logistic regression
Maternal smoking during pregnancy	12.59	Polytomous logistic regression
Maternal cannabis use	12.94	Logistic regression
Maternal education - 5 years	14.60	Polytomous logistic regression
Breakfast skipping - 6 years	14.93	Logistic regression
Satiety responsiveness - 10 years	16.16	Predictive mean matching
Enjoyment of food - 10 years	16.30	Predictive mean matching
Food responsiveness - 10 years	16.37	Predictive mean matching
Lunch skipping - 6 years	16.58	Logistic regression
Restriction - 10 years	16.74	Predictive mean matching
Dinner skipping - 6 years	17.69	Logistic regression
Emotional overeating - 10 years	18.75	Predictive mean matching
Household income - 5 years	18.92	Logistic regression
Paternal smoking during pregnancy	19.70	Logistic regression
Paternal cannabis use	20.93	Logistic regression
Television viewing	21.35	Logistic regression
Maternal vomiting and nausea	21.61	Logistic regression
Paternal education - 5 years	21.81	Polytomous logistic regression
Household income - baseline	22.35	Logistic regression
25(OH)D concentration	23.72	Predictive mean matching
Computer game use	23.92	Logistic regression
Maternal weight before pregnancy	24.74	Predictive mean matching
Maternal BMI before pregnancy	24.85	Predictive mean matching
Weight gain during infancy - 0 to 6 months	24.90	Predictive mean matching
Psychopathology symptoms (GSI)	25.34	Predictive mean matching
Postpartum depression - 20 weeks	25.36	Predictive mean matching
BMI at estimated adiposity peak	25.36	Predictive mean matching

Table S3. Percentages of missing values in each perdictor and variables used for multiple imputation *(continued)*

Incomplete variables	Percentages of missing values (%)	Method used for imputation
Total n-6 polyunsaturated fatty acids (PUFA) percentage by weight of total sum of fatty acids	25.75	Predictive mean matching
Maternal milk intake during pregnancy	28.39	Polytomous logistic regression
Outdoor play	28.46	Logistic regression
Paternal BMI - baseline	29.26	Predictive mean matching
Pressure to eat - 2 years	29.32	Predictive mean matching
Folic acid supplement use	30.51	Polytomous logistic regression
Breakfast skipping - 4 years	32.11	Logistic regression
Food responsiveness - 4 years	32.25	Predictive mean matching
Satiety responsiveness - 4 years	32.31	Predictive mean matching
Lunch skipping - 4 years	32.43	Logistic regression
Pressure to eat - 4 years	32.52	Predictive mean matching
Emotional undereating - 4 years	32.64	Predictive mean matching
Set-shifting	32.64	Predictive mean matching
Restriction - 4 years	32.73	Predictive mean matching
Enjoyment of food - 4 years	32.73	Predictive mean matching
Dinner skipping - 4 years	32.87	Logistic regression
Emotional overeating - 4 years	33.05	Predictive mean matching
Weight gain during infancy - 6 to 12 months	34.79	Predictive mean matching
zBMI - 2 years	34.93	Predictive mean matching
Paternal education - baseline	37.34	Polytomous logistic regression
Postpartum depression - 2 months	38.11	Predictive mean matching
Breastfeeding duration	42.21	Proportional odds model
BMI gain during infancy - 6 to 12 months	42.46	Predictive mean matching
Weight gain during infancy - 12 to 24 months	43.95	Predictive mean matching
Postpartum depression - 6 months	44.44	Predictive mean matching
zBMI - 1.5 months	44.44	Predictive mean matching
BMI gain during infancy - 12 to 24 months	45.25	Predictive mean matching
Timing of introduction of solid foods	45.87	Polytomous logistic regression
Animal and plant protein intake	51.79	Predictive mean matching
Hair cortisol concentration	59.22	Predictive mean matching

Table S3. Percentages of missing values in each perdictor and variables used for multiple imputation *(continued)*

Incomplete variables	Percentages of missing values (%)	Method used for imputation
Hair cortisone concentration	59.90	Predictive mean matching
Timing of introduction of solid foods	66.44	Predictive mean matching

Table S3 Percentages of missing values in each perdictor and variables used for multiple imputation *(continued)*

Incomplete variables	Variables used for imputing each incomplete variable
**Child ethnic background**	Maternal education-baseline, Total n-6 polyunsaturated fatty acids (PUFA) percentage by weight of total sum of fatty acids, 25(OH)D concentration, zBMI at age 10, Household income-5 years, Child age at BMI assessment at age 10, Parity, Maternal age
**Maternal education-baseline**	Child ethnic background, Maternal education-5 years, Paternal education-5 years, Weight gain during infancy-0 to 6 months, Enjoyment of food-10 years, Breakfast skipping-6 years, Computer game use, Television viewing, zBMI at age 10, Household income-5 years, Parity, Maternal age
**Maternal education-5 years**	Child ethnic background, Maternal education-baseline, Maternal BMI before pregnancy, Paternal BMI before pregnancy, Maternal smoking during pregnancy, Paternal smoking during pregnancy, Total n-6 polyunsaturated fatty acids (PUFA) percentage by weight of total sum of fatty acids, 25(OH)D concentration, Folic acid supplement use, Maternal vomiting and nausea, Psychopathology symptoms (GSI), Postpartum depression-20 weeks, Weight gain during infancy-0 to 6 months, Enjoyment of food-10 years, zBMI at age 10, Household income-5 years, Child age at BMI assessment at age 10, Parity, Maternal age
**Paternal education-baseline**	Child ethnic background, Maternal education-baseline, Maternal education-5 years, Paternal education-5 years, Maternal BMI before pregnancy, Maternal smoking during pregnancy, Paternal smoking during pregnancy, Maternal cannabis use, Paternal cannabis use, Total n-6 polyunsaturated fatty acids (PUFA) percentage by weight of total sum of fatty acids, 25(OH)D concentration, Folic acid supplement use, Maternal vomiting and nausea, Breakfast skipping-6 years, Computer game use, Television viewing, zBMI at age 10, Household income-5 years, Parity, Maternal age
**Paternal education-5 years**	Child ethnic background, Maternal education-baseline, Maternal BMI before pregnancy, Paternal BMI before pregnancy, Maternal smoking during pregnancy, Paternal smoking during pregnancy, Paternal cannabis use, Total n-6 polyunsaturated fatty acids (PUFA) percentage by weight of total sum of fatty acids, 25(OH)D concentration, Folic acid supplement use, Maternal vomiting and nausea, Psychopathology symptoms (GSI), Postpartum depression-20 weeks, Weight gain during infancy-0 to 6 months, zBMI at age 10, Household income-5 years, Child age at BMI assessment at age 10, Maternal age
**Maternal BMI before pregnancy**	Child ethnic background, Maternal education-baseline, Maternal education-5 years, Paternal education-5 years, 25(OH)D concentration, zBMI-1.5 months, zBMI-2 years, BMI at estimated adiposity peak, Food responsiveness-10 years, Television viewing, zBMI at age 10, Household income-5 years, Parity

Table S3 Percentages of missing values in each perdictor and variables used for multiple imputation *(continued)*

Incomplete variables	Variables used for imputing each incomplete variable
**Paternal BMI before pregnancy**	Child ethnic background, Maternal education-baseline, Maternal education-5 years, Paternal education-5 years, Maternal BMI before pregnancy, Maternal weight before pregnancy, Total n-6 polyunsaturated fatty acids (PUFA) percentage by weight of total sum of fatty acids, 25(OH)D concentration, zBMI-1.5 months, zBMI-2 years, BMI at estimated adiposity peak, Food responsiveness-10 years, zBMI at age 10, Household income-5 years, Parity
**Maternal weight before pregnancy**	Child ethnic background, Maternal education-baseline, Weight gain during infancy-6 to 12 months, Weight gain during infancy-12 to 24 months, zBMI-2 years, Food responsiveness-10 years, zBMI at age 10, Household income-5 years
**Maternal smoking during pregnancy**	Child ethnic background, Maternal education-baseline, Maternal education-5 years, Paternal education-5 years, zBMI at age 10, Household income-5 years, Maternal age
**Paternal smoking during pregnancy**	Child ethnic background, Maternal education-baseline, Maternal education-5 years, Paternal education-5 years, zBMI at age 10, Household income-5 years, Maternal age
**Maternal cannabis use**	Child ethnic background, Maternal education-baseline, 25(OH)D concentration, zBMI at age 10, Household income-5 years
**Paternal cannabis use**	Child ethnic background, Maternal education-baseline, Paternal education-5 years, zBMI at age 10, Household income-5 years, Parity, Maternal age
**Total n-6 polyunsaturated fatty acids (PUFA) percentage by weight of total sum of fatty acids**	Child ethnic background, Maternal education-baseline, Maternal education-5 years, Paternal education-5 years, Maternal BMI before pregnancy, Maternal vomiting and nausea, Postpartum depression-2 months, Television viewing, zBMI at age 10, Household income-5 years, Parity, Maternal age
**25(OH)D concentration**	Child ethnic background, Maternal education-baseline, Maternal education-5 years, Paternal education-5 years, Maternal vomiting and nausea, Breakfast skipping-6 years, zBMI at age 10, Household income-5 years, Parity, Maternal age
**Folic acid supplement use**	Child ethnic background, Maternal education-baseline, Maternal education-5 years, Paternal education-5 years, Maternal smoking during pregnancy, Total n-6 polyunsaturated fatty acids (PUFA) percentage by weight of total sum of fatty acids, 25(OH)D concentration, Psychopathology symptoms (GSI), Postpartum depression-20 weeks, Weight gain during infancy-0 to 6 months, Emotional overeating-4 years, Breakfast skipping-4 years, Lunch skipping-4 years, Breakfast skipping-6 years, Computer game use, Television viewing, zBMI at age 10, Household income-5 years, Child age at BMI assessment at age 10, Parity, Maternal age

Table S3 Percentages of missing values in each perdictor and variables used for multiple imputation *(continued)*

Incomplete variables	Variables used for imputing each incomplete variable
**Maternal vomiting and nausea**	Child ethnic background, Maternal education-baseline, Maternal education-5 years, Paternal education-5 years, zBMI at age 10, Household income-5 years, Maternal age
**Maternal milk intake during pregnancy**	Child ethnic background, Maternal education-baseline, Maternal education-5 years, Paternal education-5 years, Total n-6 polyunsaturated fatty acids (PUFA) percentage by weight of total sum of fatty acids, 25(OH)D concentration, zBMI at age 10, Household income-5 years
**Psychopathology symptoms (GSI)**	Child ethnic background, Maternal education-baseline, Maternal education-5 years, Paternal education-5 years, Maternal smoking during pregnancy, Pressure to eat-4 years, Restriction-10 years, Satiety responsiveness-4 years, Breakfast skipping-4 years, Lunch skipping-4 years, Breakfast skipping-6 years, Computer game use, Set-shifting, zBMI at age 10, Household income-5 years, Parity, Maternal age
**Postpartum depression-20 weeks**	Child ethnic background, Maternal education-baseline, Maternal education-5 years, Paternal education-5 years, Maternal smoking during pregnancy, Set-shifting, zBMI at age 10, Household income-5 years, Parity, Maternal age
**Postpartum depression-2 months**	Child ethnic background, Maternal education-baseline, Maternal education-5 years, Paternal education-5 years, Total n-6 polyunsaturated fatty acids (PUFA) percentage by weight of total sum of fatty acids, 25(OH)D concentration, Folic acid supplement use, Maternal vomiting and nausea, Psychopathology symptoms (GSI), Postpartum depression-20 weeks, Restriction-10 years, zBMI at age 10, Household income-5 years, Maternal age
**Postpartum depression-6 months**	Child ethnic background, Maternal education-baseline, Maternal education-5 years, Paternal education-baseline, Paternal education-5 years, Maternal smoking during pregnancy, Paternal smoking during pregnancy, Total n-6 polyunsaturated fatty acids (PUFA) percentage by weight of total sum of fatty acids, 25(OH)D concentration, Folic acid supplement use, Maternal vomiting and nausea, Psychopathology symptoms (GSI), Postpartum depression-20 weeks, Computer game use, zBMI at age 10, Household income-5 years, Child age at BMI assessment at age 10, Maternal age
**Pressure to eat-2 years**	Child ethnic background, Maternal education-baseline, Maternal education-5 years, Paternal education-5 years, Maternal smoking during pregnancy, Total n-6 polyunsaturated fatty acids (PUFA) percentage by weight of total sum of fatty acids, 25(OH)D concentration, Folic acid supplement use, Maternal vomiting and nausea, Psychopathology symptoms (GSI), Postpartum depression-20 weeks, Computer game use, Television viewing, zBMI at age 10, Household income-5 years, Maternal age

Table S3 Percentages of missing values in each perdictor and variables used for multiple imputation *(continued)*

**Incomplete variables**	**Variables used for imputing each incomplete variable**
**Pressure to eat-4 years**	Child ethnic background, Maternal education-baseline, Maternal education-5 years, Paternal education-5 years, Maternal BMI before pregnancy, Maternal smoking during pregnancy, Total n-6 polyunsaturated fatty acids (PUFA) percentage by weight of total sum of fatty acids, 25(OH)D concentration, Folic acid supplement use, Maternal vomiting and nausea, Psychopathology symptoms (GSI), Postpartum depression-20 weeks, Enjoyment of food-10 years, Food responsiveness-10 years, Satiety responsiveness-10 years, Breakfast skipping-6 years, Television viewing, zBMI at age 10, Household income-5 years, Child age at BMI assessment at age 10, Maternal age
**Restriction-4 years**	Child ethnic background, Maternal education-baseline, Maternal education-5 years, Paternal education-5 years, Maternal BMI before pregnancy, Maternal smoking during pregnancy, Total n-6 polyunsaturated fatty acids (PUFA) percentage by weight of total sum of fatty acids, 25(OH)D concentration, Folic acid supplement use, Maternal vomiting and nausea, Psychopathology symptoms (GSI), Postpartum depression-20 weeks, Restriction-10 years, Emotional overeating-10 years, Food responsiveness-10 years, Breakfast skipping-6 years, Television viewing, zBMI at age 10, Household income-5 years, Child age at BMI assessment at age 10, Maternal age
**Restriction-10 years**	Child ethnic background, Maternal education-baseline, Maternal education-5 years, Maternal smoking during pregnancy, Total n-6 polyunsaturated fatty acids (PUFA) percentage by weight of total sum of fatty acids, 25(OH)D concentration, Folic acid supplement use, Psychopathology symptoms (GSI), Postpartum depression-20 weeks, zBMI-2 years, zBMI at age 10, Household income-5 years, Child age at BMI assessment at age 10, Maternal age
**Timing of introduction of solid foods**	Child ethnic background, Maternal education-baseline, Maternal education-5 years, Paternal education-baseline, Paternal education-5 years, Maternal BMI before pregnancy, Maternal smoking during pregnancy, Paternal smoking during pregnancy, Total n-6 polyunsaturated fatty acids (PUFA) percentage by weight of total sum of fatty acids, 25(OH)D concentration, Folic acid supplement use, Maternal vomiting and nausea, Psychopathology symptoms (GSI), Weight gain during infancy-0 to 6 months, Television viewing, zBMI at age 10, Household income-5 years, Child age at BMI assessment at age 10, Maternal age

Table S3 Percentages of missing values in each perdictor and variables used for multiple imputation *(continued)*

Incomplete variables	Variables used for imputing each incomplete variable
**Breastfeeding duration**	Child ethnic background, Maternal education-baseline, Maternal education-5 years, Paternal education-baseline, Paternal education-5 years, Maternal BMI before pregnancy, Maternal smoking during pregnancy, Total n-6 polyunsaturated fatty acids (PUFA) percentage by weight of total sum of fatty acids, 25(OH)D concentration, Folic acid supplement use, Psychopathology symptoms (GSI), Weight gain during infancy-0 to 6 months, Television viewing, zBMI at age 10, Household income-5 years, Parity, Maternal age
**Weight gain during infancy-0 to 6 months**	Child ethnic background, Maternal education-baseline, Maternal education-5 years, Paternal education-5 years, Total n-6 polyunsaturated fatty acids (PUFA) percentage by weight of total sum of fatty acids, 25(OH)D concentration, Folic acid supplement use, Computer game use, zBMI at age 10, Household income-5 years, Child sex, Maternal age
**Weight gain during infancy-6 to 12 months**	Child ethnic background, Maternal education-baseline, Maternal education-5 years, Paternal education-baseline, Paternal education-5 years, Maternal weight before pregnancy, Total n-6 polyunsaturated fatty acids (PUFA) percentage by weight of total sum of fatty acids, 25(OH)D concentration, Folic acid supplement use, Satiety responsiveness-10 years, zBMI at age 10, Household income-5 years, Maternal age
**Weight gain during infancy-12 to 24 months**	Child ethnic background, Maternal education-baseline, Maternal education-5 years, Paternal education-baseline, Paternal education-5 years, Maternal weight before pregnancy, Total n-6 polyunsaturated fatty acids (PUFA) percentage by weight of total sum of fatty acids, 25(OH)D concentration, Folic acid supplement use, Enjoyment of food-10 years, Food responsiveness-10 years, Satiety responsiveness-10 years, zBMI at age 10, Household income-5 years, Maternal age
**zBMI-1.5 months**	Child ethnic background, Maternal education-baseline, Maternal education-5 years, Paternal education-baseline, Paternal education-5 years, Maternal BMI before pregnancy, Paternal BMI before pregnancy, Folic acid supplement use, Weight gain during infancy-0 to 6 months, Computer game use, zBMI at age 10, Household income-5 years, Parity
**zBMI-2 years**	Child ethnic background, Maternal education-baseline, Maternal education-5 years, Paternal education-baseline, Paternal education-5 years, Maternal BMI before pregnancy, Paternal BMI before pregnancy, Maternal weight before pregnancy, Folic acid supplement use, Restriction-10 years, Enjoyment of food-10 years, Food responsiveness-10 years, Satiety responsiveness-10 years, zBMI at age 10, Household income-5 years, Maternal age

Table S3 Percentages of missing values in each perdictor and variables used for multiple imputation *(continued)*

Incomplete variables	Variables used for imputing each incomplete variable
**BMI gain-6 to 12 months**	Child ethnic background, Maternal education-baseline, Maternal education-5 years, Paternal education-baseline, Paternal education-5 years, Total n-6 polyunsaturated fatty acids (PUFA) percentage by weight of total sum of fatty acids, 25(OH)D concentration, Folic acid supplement use, zBMI at age 10, Household income-5 years, Maternal age
**BMI gain-12 to 24 months**	Child ethnic background, Maternal education-baseline, Maternal education-5 years, Paternal education-baseline, Paternal education-5 years, Total n-6 polyunsaturated fatty acids (PUFA) percentage by weight of total sum of fatty acids, 25(OH)D concentration, Folic acid supplement use, Weight gain during infancy-0 to 6 months, zBMI at age 10, Household income-5 years, Maternal age
**BMI at estimated adiposity peak**	Child ethnic background, Maternal education-baseline, Maternal education-5 years, Paternal education-baseline, Paternal education-5 years, Maternal BMI before pregnancy, Paternal BMI before pregnancy, Total n-6 polyunsaturated fatty acids (PUFA) percentage by weight of total sum of fatty acids, 25(OH)D concentration, Folic acid supplement use, Food responsiveness-10 years, Satiety responsiveness-10 years, Computer game use, zBMI at age 10, Household income-5 years, Child sex, Parity, Maternal age
**High fat-mass dietary pattern**	Child ethnic background, Maternal education-baseline, Maternal education-5 years, Paternal education-baseline, Paternal education-5 years, Maternal BMI before pregnancy, Maternal smoking during pregnancy, Paternal smoking during pregnancy, Total n-6 polyunsaturated fatty acids (PUFA) percentage by weight of total sum of fatty acids, 25(OH)D concentration, Folic acid supplement use, Maternal vomiting and nausea, Psychopathology symptoms (GSI), Postpartum depression-20 weeks, Postpartum depression-2 months, Pressure to eat-4 years, Weight gain during infancy-0 to 6 months, Breakfast skipping-6 years, Computer game use, Television viewing, zBMI at age 10, Household income-5 years, Maternal age
**Animal and plant protein intake**	Child ethnic background, Maternal education-baseline, Maternal education-5 years, Paternal education-baseline, Paternal education-5 years, Paternal smoking during pregnancy, Total n-6 polyunsaturated fatty acids (PUFA) percentage by weight of total sum of fatty acids, 25(OH)D concentration, Folic acid supplement use, Psychopathology symptoms (GSI), Postpartum depression-20 weeks, Weight gain during infancy-0 to 6 months, Computer game use, Television viewing, zBMI at age 10, Household income-5 years, Maternal age

Table S3 Percentages of missing values in each perdictor and variables used for multiple imputation *(continued)*

Incomplete variables	Variables used for imputing each incomplete variable
**Emotional undereating-4 years**	Child ethnic background, Maternal education-baseline, Maternal education-5 years, Paternal education-5 years, Maternal BMI before pregnancy, Maternal smoking during pregnancy, Total n-6 polyunsaturated fatty acids (PUFA) percentage by weight of total sum of fatty acids, 25(OH)D concentration, Folic acid supplement use, Maternal vomiting and nausea, Psychopathology symptoms (GSI), Postpartum depression-20 weeks, Emotional overeating-10 years, Satiety responsiveness-10 years, Breakfast skipping-6 years, Television viewing, zBMI at age 10, Household income-5 years, Child age at BMI assessment at age 10, Maternal age
**Emotional overeating-4 years**	Child ethnic background, Maternal education-baseline, Maternal education-5 years, Paternal education-5 years, Maternal BMI before pregnancy, Maternal smoking during pregnancy, Total n-6 polyunsaturated fatty acids (PUFA) percentage by weight of total sum of fatty acids, 25(OH)D concentration, Folic acid supplement use, Maternal vomiting and nausea, Psychopathology symptoms (GSI), Postpartum depression-20 weeks, Restriction-10 years, Emotional overeating-10 years, Breakfast skipping-6 years, Television viewing, zBMI at age 10, Household income-5 years, Child age at BMI assessment at age 10, Maternal age
**Enjoyment of food-4 years**	Child ethnic background, Maternal education-baseline, Maternal education-5 years, Paternal education-5 years, Maternal BMI before pregnancy, Maternal smoking during pregnancy, Total n-6 polyunsaturated fatty acids (PUFA) percentage by weight of total sum of fatty acids, 25(OH)D concentration, Folic acid supplement use, Maternal vomiting and nausea, Psychopathology symptoms (GSI), Postpartum depression-20 weeks, Enjoyment of food-10 years, Food responsiveness-10 years, Satiety responsiveness-10 years, Breakfast skipping-6 years, Television viewing, zBMI at age 10, Household income-5 years, Child age at BMI assessment at age 10, Maternal age
**Food responsiveness-4 years**	Child ethnic background, Maternal education-baseline, Maternal education-5 years, Paternal education-5 years, Maternal BMI before pregnancy, Maternal smoking during pregnancy, Total n-6 polyunsaturated fatty acids (PUFA) percentage by weight of total sum of fatty acids, 25(OH)D concentration, Folic acid supplement use, Maternal vomiting and nausea, Psychopathology symptoms (GSI), Postpartum depression-20 weeks, Restriction-10 years, Emotional overeating-10 years, Enjoyment of food-10 years, Food responsiveness-10 years, Satiety responsiveness-10 years, Breakfast skipping-6 years, Television viewing, zBMI at age 10, Household income-5 years, Child age at BMI assessment at age 10, Maternal age

Table S3 Percentages of missing values in each perdictor and variables used for multiple imputation *(continued)*

Incomplete variables	Variables used for imputing each incomplete variable
**Satiety responsiveness-4 years**	Child ethnic background, Maternal education-baseline, Maternal education-5 years, Paternal education-5 years, Maternal BMI before pregnancy, Maternal smoking during pregnancy, Total n-6 polyunsaturated fatty acids (PUFA) percentage by weight of total sum of fatty acids, 25(OH)D concentration, Folic acid supplement use, Maternal vomiting and nausea, Psychopathology symptoms (GSI), Postpartum depression-20 weeks, Enjoyment of food-10 years, Food responsiveness-10 years, Satiety responsiveness-10 years, Breakfast skipping-6 years, Television viewing, zBMI at age 10, Household income-5 years, Child age at BMI assessment at age 10, Maternal age
**Emotional overeating-10 years**	Child ethnic background, Maternal education-baseline, Maternal education-5 years, Paternal education-5 years, Total n-6 polyunsaturated fatty acids (PUFA) percentage by weight of total sum of fatty acids, 25(OH)D concentration, Folic acid supplement use, Psychopathology symptoms (GSI), Postpartum depression-20 weeks, zBMI at age 10, Household income-5 years, Child age at BMI assessment at age 10, Parity, Maternal age
**Enjoyment of food-10 years**	Child ethnic background, Maternal education-baseline, Maternal education-5 years, Total n-6 polyunsaturated fatty acids (PUFA) percentage by weight of total sum of fatty acids, 25(OH)D concentration, Folic acid supplement use, Psychopathology symptoms (GSI), Postpartum depression-20 weeks, zBMI-2 years, zBMI at age 10, Household income-5 years, Child age at BMI assessment at age 10, Maternal age
**Food responsiveness-10 years**	Child ethnic background, Maternal education-baseline, Maternal education-5 years, Maternal BMI before pregnancy, Paternal BMI before pregnancy, Maternal weight before pregnancy, Total n-6 polyunsaturated fatty acids (PUFA) percentage by weight of total sum of fatty acids, 25(OH)D concentration, Folic acid supplement use, Psychopathology symptoms (GSI), Postpartum depression-20 weeks, zBMI-2 years, BMI at estimated adiposity peak, zBMI at age 10, Household income-5 years, Child age at BMI assessment at age 10, Maternal age
**Satiety responsiveness-10 years**	Child ethnic background, Maternal education-baseline, Maternal education-5 years, Total n-6 polyunsaturated fatty acids (PUFA) percentage by weight of total sum of fatty acids, 25(OH)D concentration, Folic acid supplement use, Psychopathology symptoms (GSI), Postpartum depression-20 weeks, zBMI-2 years, BMI at estimated adiposity peak, zBMI at age 10, Household income-5 years, Child age at BMI assessment at age 10, Maternal age

Table S3 Percentages of missing values in each perdictor and variables used for multiple imputation *(continued)*

Incomplete variables	Variables used for imputing each incomplete variable
**Breakfast skipping-4 years**	Child ethnic background, Maternal education-baseline, Maternal education-5 years, Paternal education-5 years, Maternal BMI before pregnancy, Maternal smoking during pregnancy, Total n-6 polyunsaturated fatty acids (PUFA) percentage by weight of total sum of fatty acids, 25(OH)D concentration, Folic acid supplement use, Maternal vomiting and nausea, Psychopathology symptoms (GSI), Postpartum depression-20 weeks, Breakfast skipping-6 years, Lunch skipping-6 years, Computer game use, Television viewing, zBMI at age 10, Household income-5 years, Child age at BMI assessment at age 10, Maternal age
**Lunch skipping-4 years**	Child ethnic background, Maternal education-baseline, Maternal education-5 years, Paternal education-5 years, Maternal BMI before pregnancy, Maternal smoking during pregnancy, Total n-6 polyunsaturated fatty acids (PUFA) percentage by weight of total sum of fatty acids, 25(OH)D concentration, Folic acid supplement use, Maternal vomiting and nausea, Psychopathology symptoms (GSI), Postpartum depression-20 weeks, Breakfast skipping-6 years, Lunch skipping-6 years, Computer game use, Television viewing, zBMI at age 10, Household income-5 years, Child age at BMI assessment at age 10, Maternal age
**Dinner skipping-4 years**	Child ethnic background, Maternal education-baseline, Maternal education-5 years, Paternal education-5 years, Maternal BMI before pregnancy, Maternal smoking during pregnancy, Total n-6 polyunsaturated fatty acids (PUFA) percentage by weight of total sum of fatty acids, 25(OH)D concentration, Folic acid supplement use, Maternal vomiting and nausea, Psychopathology symptoms (GSI), Postpartum depression-20 weeks, Breakfast skipping-6 years, Lunch skipping-6 years, Dinner skipping-6 years, Television viewing, zBMI at age 10, Household income-5 years, Child age at BMI assessment at age 10, Maternal age
**Breakfast skipping-6 years**	Child ethnic background, Maternal education-baseline, Total n-6 polyunsaturated fatty acids (PUFA) percentage by weight of total sum of fatty acids, 25(OH)D concentration, Folic acid supplement use, Psychopathology symptoms (GSI), Postpartum depression-20 weeks, zBMI at age 10, Household income-5 years, Child age at BMI assessment at age 10, Maternal age
**Lunch skipping-6 years**	Child ethnic background, Maternal education-baseline, Total n-6 polyunsaturated fatty acids (PUFA) percentage by weight of total sum of fatty acids, 25(OH)D concentration, Folic acid supplement use, Psychopathology symptoms (GSI), Postpartum depression-20 weeks, zBMI at age 10, Household income-5 years, Child age at BMI assessment at age 10, Maternal age

Table S3 Percentages of missing values in each perdictor and variables used for multiple imputation *(continued)*

Incomplete variables	Variables used for imputing each incomplete variable
**Dinner skipping-6 years**	Child ethnic background, Maternal education-baseline, Total n-6 polyunsaturated fatty acids (PUFA) percentage by weight of total sum of fatty acids, 25(OH)D concentration, Folic acid supplement use, Psychopathology symptoms (GSI), Postpartum depression-20 weeks, zBMI at age 10, Household income-5 years, Child age at BMI assessment at age 10, Maternal age
**Computer game use**	Child ethnic background, Maternal education-baseline, Total n-6 polyunsaturated fatty acids (PUFA) percentage by weight of total sum of fatty acids, 25(OH)D concentration, Folic acid supplement use, Psychopathology symptoms (GSI), Postpartum depression-20 weeks, Weight gain during infancy-0 to 6 months, zBMI at age 10, Household income-5 years, Child sex, Child age at BMI assessment at age 10, Maternal age
**Television viewing**	Child ethnic background, Maternal education-baseline, Maternal BMI before pregnancy, Total n-6 polyunsaturated fatty acids (PUFA) percentage by weight of total sum of fatty acids, 25(OH)D concentration, Folic acid supplement use, Psychopathology symptoms (GSI), Postpartum depression-20 weeks, zBMI at age 10, Household income-5 years, Child age at BMI assessment at age 10, Maternal age
**Outdoor play**	Child ethnic background, Maternal education-baseline, Maternal education-5 years, Total n-6 polyunsaturated fatty acids (PUFA) percentage by weight of total sum of fatty acids, 25(OH)D concentration, Folic acid supplement use, Maternal vomiting and nausea, Psychopathology symptoms (GSI), Postpartum depression-20 weeks, zBMI at age 10, Household income-5 years, Child age at BMI assessment at age 10, Maternal age
**Set-shifting**	Child ethnic background, Maternal education-baseline, Maternal education-5 years, Paternal education-5 years, Maternal BMI before pregnancy, Maternal smoking during pregnancy, Total n-6 polyunsaturated fatty acids (PUFA) percentage by weight of total sum of fatty acids, 25(OH)D concentration, Folic acid supplement use, Maternal vomiting and nausea, Psychopathology symptoms (GSI), Postpartum depression-20 weeks, Breakfast skipping-6 years, Television viewing, zBMI at age 10, Household income-5 years, Child sex, Child age at BMI assessment at age 10, Maternal age
**Hair cortisol**	Child ethnic background, Maternal education-baseline, Maternal education-5 years, 25(OH)D concentration, Folic acid supplement use, Psychopathology symptoms (GSI), Postpartum depression-20 weeks, zBMI at age 10, Household income-5 years
**Hair cortisone**	Child ethnic background, Maternal education-baseline, Hair cortisol, zBMI at age 10, Household income-5 years, Child sex

Table S3 Percentages of missing values in each perdictor and variables used for multiple imputation *(continued)*

**Household income-5 years**	Child ethnic background, Maternal education-baseline, Maternal BMI before pregnancy, Maternal smoking during pregnancy, Paternal smoking during pregnancy, Paternal cannabis use, Total n-6 polyunsaturated fatty acids (PUFA) percentage by weight of total sum of fatty acids, 25(OH)D concentration, Folic acid supplement use, Maternal vomiting and nausea, Psychopathology symptoms (GSI), Postpartum depression-20 weeks, Weight gain during infancy-0 to 6 months, zBMI at age 10, Child age at BMI assessment at age 10, Maternal age
**Household income-baseline**	Child ethnic background, Maternal education-baseline, Maternal education-5 years, Paternal education-5 years, Maternal smoking during pregnancy, Weight gain during infancy-0 to 6 months, Breakfast skipping-6 years, Computer game use, Television viewing, zBMI at age 10, Household income-5 years, Parity, Maternal age
**Child sex**	Child ethnic background, Maternal education-baseline, Computer game use, Hair cortisone, zBMI at age 10, Household income-5 years
**Parity**	Child ethnic background, Maternal education-baseline, Maternal education-5 years, BMI at estimated adiposity peak, zBMI at age 10, Household income-5 years, Maternal age
**Maternal age**	Child ethnic background, Maternal education-baseline, Maternal smoking during pregnancy, Paternal smoking during pregnancy, Paternal cannabis use, Folic acid supplement use, Maternal vomiting and nausea, Psychopathology symptoms (GSI), Postpartum depression-20 weeks, zBMI at age 10, Household income-5 years, Child age at BMI assessment at age 10

Table S4. Overview of included papers[1]

Authors	Years	Domains	Predictors	Age at predictors assessment	Age at BMI assessment	Sample size
O. Gishti, C. J. Kruithof et al.	2014	Sociodemographic factors	Ethnicity	At enrollment	6 years	5244
O. Gishti, R. Gaillard, J. F. Felix et al.	2015	Sociodemographic factors	Ethnicity	At enrollment	6 years	5054
R. Gaillard, A. A. Rurangirwa et al.	2014	Sociodemographic factors	Parity	First trimester	6 years	6295
L. van Rossem et al.	2010	Sociodemographic factors	Maternal education	At enrollment	3 years	2954
S. H. Bouthoorn, A. I. Wijtzes et al.	2014	Sociodemographic factors	Maternal education	At enrollment	6 years	3010
L. Lin et al.	2021	Sociodemographic factors	Maternal education	At enrollment	10 years	4030
L. Ay el al.	2008	Sociodemographic factors	Sex	At enrollment	2 years	1012
E. T. Leermakers, J. F. Felix, N. S. Erler et al.	2015	Sociodemographic factors	Sex	At enrollment	6 years	2045
S. Vogelezang et al.	2016	Sociodemographic factors	Sex	At enrollment	6 years	393
T. Marinkovic et al.	2017	Sociodemographic factors	Sex	At enrollment	6 years	4649
A. N. Nguyen et al.	2020	Sociodemographic factors	Sex	At enrollment	10 years	3991
A. Elhakeem et al.	2022	Preconception and prenatal parental health	Assisted reproductive technology use	At enrollment	10 years	3796
M. N. Kooijman et al.	2016	Preconception and prenatal parental health	Umbilical artery pulsatility index	Third trimester	6 years	1195
R. Gaillard et al.	2013	Preconception and prenatal parental health	Umbilical artery pulsatility index	Third trimester	6 years	4722
J. L. Vinther et al.	2023	Preconception and prenatal parental health	Gestational age	At enrollment	10 years	3870
M. Lecorguille el al.	2023	Preconception and prenatal parental health	High parental smoking, and low maternal diet quality	First, second, and third trimesters	10 years	4944
B. Durmus, L. Ay et al.	2011	Preconception and prenatal parental health	Maternal smoking	First, second, and third trimesters	2 years	671
B. Durmus, C. J. Kruithof et al.	2011	Preconception and prenatal parental health	Maternal smoking	First, second, and third trimesters	4 years	5342
K. N. Cajachagua-Torres et al.	2022	Preconception and prenatal parental health	Maternal tabacco use	First, second, and third trimesters	10 years	4252
B. Durmus, D. H. Heppe, H. R. Taal et al.	2014	Preconception and prenatal parental health	Paternal smoking	First, second, and third trimesters	6 years	3832

Table S4. Overview of included papers[1] *(continued)*

Authors	Years	Domains	Predictors	Age at predictors assessment	Age at BMI assessment	Sample size
M. Welten et al.	2015	Preconception and prenatal parental health	Haemoglobin	Second trimester	6 years	4995
R. J. Wahab et al.	2021	Preconception and prenatal parental health	Maternal dietary glycemic index	First trimester	10 years	2483
R. J. Wahab et al.	2020	Preconception and prenatal parental health	Maternal glucose concentrations	First trimester	10 years	3726
G. A. Godoy et al.	2014	Preconception and prenatal parental health	Thyroid stimulating hormone levels	First trimester	6 years	3985
R. Gaillard et al.	2015	Preconception and prenatal parental health	Gestational weight gain	First trimester	6 years	4062
V. V. Jharap et al.	2017	Preconception and prenatal parental health	Maternal BMI	Preconception	2 years	659
R. C. Richmond et al.	2017	Preconception and prenatal parental health	Maternal BMI	Preconception	6 years	2337
R. Gaillard, E. A. Steegers el al.	2014	Preconception and prenatal parental health	Maternal BMI	Preconception	6 years	4871
L. Benschop et al.	2018	Preconception and prenatal parental health	Maternal BMI	Preconception	10 years	4479
S. Santos et al.	2019	Preconception and prenatal parental health	Maternal BMI	Preconception	10 years	2354
B. Durmus, L. R. Arends et al.	2013	Preconception and prenatal parental health	Paternal BMI	Preconception	4 years	3055
D. V. Gootjes et al.	2021	Preconception and prenatal parental health	Hypertensive disorders of pregnancy	At enrollment	6 years	5312
A. Jelena Vidakovic, S. Santos et al.	2016	Maternal nutrition during pregnancy	Total n-3 PUFA	Second trimester	2 years	904
A. J. Vidakovic, O. Gishti et al.	2016	Maternal nutrition during pregnancy	Total n-3 PUFA	Second trimester	6 years	4830
T. Voortman et al.	2018	Maternal nutrition during pregnancy	n-6 PUFA pattern	Second trimester	6 years	4890
E. Voerman et al.	2016	Maternal nutrition during pregnancy	Cafeine intake	First, second, and third trimesters	6 years	5562

Table S4. Overview of included papers[1] *(continued)*

Authors	Years	Domains	Predictors	Age at predictors assessment	Age at BMI assessment	Sample size
E. Voerman et al.	2020	Maternal nutrition during pregnancy	Caffeine intake	First, second, and third trimesters	10 years	4754
E. Voerman et al.	2021	Maternal nutrition during pregnancy	Milk intake	First trimester	10 years	2461
V. Jen et al.	2017	Maternal nutrition during pregnancy	Total sugar-containing beverage intake	First trimester	6 years	3312
M. van den Broek et al.	2015	Maternal nutrition during pregnancy	Vegetable, fish, and oil dietary pattern	First trimester	6 years	2689
H. G. Quezada-Pinedo et al.	2023	Maternal nutrition during pregnancy	Ferritin	First trimester	10 years	3711
S. Poeran-Bahadoer et al.	2020	Maternal nutrition during pregnancy	Maternal vomiting	First trimester	6 years	4760
M. J. Tielemans et al.	2017	Maternal nutrition during pregnancy	Total protein intake	First trimester	6 years	2694
G. S. Monasso et al.	2021	Maternal nutrition during pregnancy	Folate	First trimester	10 years	3701
K. Miliku et al.	2018	Maternal nutrition during pregnancy	Maternal 25 (OH) D	Second trimester	6 years	4898
C. Monnereau et al.	2016	Genetics	Adult BMI genetic risk score	At birth	6 years	3975
S. Vogelezang et al.	2015	Genetics	Adult BMI genetic risk score	At birth	6 years	4144
C. Monnereau et al.	2017	Genetics	Child BMI genetic risk score	At birth	4 years	2413
C. Monnereau et al.	2018	Genetics	Child BMI genetic risk score	At birth	10 years	1993
F. P. Velders et al.	2012	Genetics	Child FTO	At birth	4 years	1718
J. A. Maas et al.	2010	Genetics	INS VNTR	At birth	2 years	738
S. H. Bouthoorn, F. J. van Lenthe et al.	2014	Genetics	PROP taster status	6 year(s)	6 years	5894
B. Durmus, D. H. Heppe, O. Gisht et al.	2014	Maternal mental health and parenting	Breastfeeding	2, 6 and 12 months	6 years	5063

Table S4. Overview of included papers[1] *(continued)*

Authors	Years	Domains	Predictors	Age at predictors assessment	Age at BMI assessment	Sample size
S. Vogelezang et al.	2018	Maternal mental health and parenting	Breastfeeding	2, 6 and 12 months	10 years	4444
K. A. Ertel et al.	2012	Maternal mental health and parenting	Depression	Second trimester	3 years	6782
M. Guxens et al.	2013	Maternal mental health and parenting	Depression	Second trimester	4 years	5273
F. O. L. Vehmeijer et al.	2019	Maternal mental health and parenting	Distress	Second trimester	10 years	4147
P. W. Jansen et al.	2014	Maternal mental health and parenting	Pressure to eat	2 year(s)	4 years	2361
I. P. Derks, H. Tiemeier et al.	2017	Maternal mental health and parenting	Restriction	10 year(s)	10 years	4689
P. W. Jansen et al.	2020	Maternal mental health and parenting	Using food as reward	4 year(s)	10 years	3642
S. Santos et al.	2016	Early-life weight and weight gain	BMI-1.5mo	1.5 months	6 years	742
B. Patro Golab et al.	2019	Early-life weight and weight gain	Total subcutaneous fat mass	2 months	10 years	593
V. W. Jaddoe et al.	2014	Early-life weight and weight gain	Fetal growth restriction	First trimester	6 years	1184
O. Gishti, R. Gaillard, R. Manniesing et al.	2014	Early-life weight and weight gain	Length gain fetal	Second trimester	6 years	5447
C. J. Kruithof et al.	2016	Early-life weight and weight gain	Peak weight velocity	36 months	6 years	5118
S. Vogelezang et al.	2019	Early-life weight and weight gain	Weight status for gestational age	At birth	10 years	3205
H. R. Taal et al.	2013	Early-life weight and weight gain	Weight strata for gestational age combined with catch up/down growth	0-2 year(s)	4 years	3531
A. N. Nguyen et al.	2020	Infant and childhood nutrition	Total carbohydrate intake (10g/d)	1 year(s)	10 years	3573
W. Stroobant et al.	2017	Infant and childhood nutrition	Total fat intake (5E%)	1 year(s)	6 years	2967
T. Voortman, E. T. Leermakers et al.	2016	Infant and childhood nutrition	Diet quality score	1 year(s)	6 years	2026
E. T. Leermakers, J. F. Felix, V. W. Jaddoe et al.	2015	Infant and childhood nutrition	Sugar cotaining beverage intake	1 year(s)	6 years	2371

Table S4. Overview of included papers[1] *(continued)*

Authors	Years	Domains	Predictors	Age at predictors assessment	Age at BMI assessment	Sample size
T. Voortman, K. V. Braun et.al	2016	Infant and childhood nutrition	Total protein intake	1 year(s)	6 years	2911
K. V. Braun et al.	2016	Infant and childhood nutrition	Total protein intake	1 year(s)	10 years	3564
V. Jen et al.	2019	Infant and childhood nutrition	Total protein intake (5E%)	1 year(s)	10 years	3573
E. T. Leermakers, J. C. Kiefte-de Jong	2015	Infant and childhood nutrition	Lutein intake	1 year(s)	6 years	2440
K. V. Braun et al.	2015	Infant and childhood nutrition	Vitamin B6	1 year(s)	6 years	2922
A. I. Wijtzes et al.	2016	Child behaviors	Breakfast skipping	4 year(s)	6 years	5913
I. P. M. Derks et al.	2018	Child behaviors	Emotional overeating	10 year(s)	10 years	3331
P. W. Jansen et al.	2019	Child behaviors	Emotional overeating	4 year(s)	10 years	3960
P. W. Jansen et al.	2012	Child behaviors	Emotional undereating	4 year(s)	4 years	3157
L. M. de Barse et al.	2015	Child behaviors	Fussy eating profile	4 year(s)	6 years	4191
A. B. Bowling et al.	2018	Child behaviors	ADHD	9 year(s)	10 years	3901
I. P. M. Derks et al.	2019	Child behaviors	Aggressive behavior	6 year(s)	10 years	3974
R. Camfferman et al.	2016	Child behaviors	Externalizing	2 year(s)	6 years	4961
J. D. Mackenbach et al.	2012	Child behaviors	Internalizing	3 year(s)	4 years	3137
C. Steegers et al.	2021	Child behaviors	Set-shifting	4 year(s)	10 years	3829
I. P. M. Derks, D. Kocevska et al.	2017	Child behaviors	Sleep duration	2 months	6 years	3909
H. A. Harris et al.	2022	Child behaviors	ADHD traits	6 year(s)	14 years	3438
A. I. Wijtzes et al.	2013	Child behaviors	Sendentary behavior	2 year(s)	2 years	347
A. I. Wijtzes et al.	2014	Child behaviors	TV vieweing	6 year(s)	6 years	5913
M. A. Jansen et al.	2015	Biological factors	Anti-tissue Transglutaminase concentrations	6 year(s)	6 years	4306
G. Noppe et al.	2016	Biological factors	Cortisol	6 year(s)	6 years	2953
O. L. Florianne	2021	Biological factors	Cortisol	6 year(s)	10 years	2042

[1] Gray shading is used to visually distinguish predictors from different domains.

Table S5. Pooled effect sizes of predictors in sociodemographics factors domain[1]

Predictors	Reference	Number of effect sizes	BMI time point	Cohen's d	CI.low	CI.hi
Maternal education	High	2	10	**0.57**	**0.53**	**0.61**
Ethnicity	Dutch	12	6	**0.39**	**0.26**	**0.52**
Paternal education	High	2	10	**0.37**	**0.25**	**0.48**
Maternal education	High	3	6	**0.03**	**-0.01**	**0.06**
Sex	Boys	3	6	**0.03**	**-0.02**	**0.07**
Sex	Boys	1	10	**0.01**	**-0.02**	**0.04**
Parity	Parity=0	4	6	**-0.07**	**-0.12**	**-0.02**
Maternal education	High	3	3	**-0.04**	**-0.07**	**-0.01**
Sex	Boys	1	2	**-0.04**	**-0.10**	**0.02**

[1] In the Cohen's d column, red highlights represent summary effect sizes with $|d|\geq 0.1$, and blue highlights represent summary effect sizes with $|d|<0.1$.

Table S6. Pooled effect sizes of predictors in preconception and prenatal parental health domain[1]

Predictors	Reference	Number of effect sizes	BMI time point	Cohen's d	ci.low	ci.hi
Maternal pre-pregnancy weight	NA	1	10	0.31	**0.25**	**0.37**
High parental BMI and low smoking	NA	1	10	0.28	**0.25**	**0.30**
Maternal BMI	NA	2	10	0.28	**0.11**	**0.45**
Maternal BMI	NA	3	6	0.25	**0.19**	**0.31**
High maternal BMI and low smoking	NA	1	10	0.24	**0.22**	**0.27**
Paternal BMI	NA	1	6	0.22	**0.20**	**0.25**
Maternal smoking	No	2	6	0.1	**-0.11**	**0.30**
Paternal BMI	NA	1	2	0.1	**0.02**	**0.18**
Maternal BMI	NA	1	2	0.19	**0.12**	**0.27**
Maternal Pre-pregnant weight	NA	1	2	0.19	**0.12**	**0.26**
Paternal BMI	NA	1	4	0.18	**0.15**	**0.22**
Maternal cannabis use	No	3	10	0.17	**0.07**	**0.28**
Maternal BMI	NA	1	4	0.17	**0.14**	**0.21**
High parental smoking, high paternal BMI, and dhigh maternal diet quality	NA	1	10	0.16	**0.13**	**0.19**
Paternal cannabis use	No	1	10	0.13	**0.10**	**0.16**
Paternal smoking	No	1	6	0.13	**0.10**	**0.16**
Paternal tabacco use	No	1	10	0.13	**0.10**	**0.16**
Umbilical artery resistance index	NA	1	6	0	**-0.03**	**0.03**
Gestational age	NA	1	10	0	**-0.03**	**0.03**
Maternal tabacco use	No	2	10	0.09	**-0.05**	**0.23**
Maternal insulin concentrations	NA	1	10	0.08	**0.05**	**0.11**
Gestational weight gain	NA	1	4	0.08	**0.04**	**0.12**
Gestational weight gain	NA	1	10	0.08	**0.03**	**0.13**
High parental smoking, and low maternal diet quality	NA	1	10	0.05	**0.02**	**0.08**
Maternal smoking	No	4	2	0.05	**-0.01**	**0.12**
Maternal smoking	No	4	4	0.05	**0.00**	**0.10**

Table S6. Pooled effect sizes of predictors in preconception and prenatal parental health domain[1] *(continued)*

Predictors	Reference	Number of effect sizes	BMI time point	Cohen's d	ci.low	ci.hi
Maternal dietary glycemic index	NA	1	10	**0.05**	**0.01**	**0.09**
Maternal weight during pregnancy	NA	3	10	**0.05**	**-0.05**	**0.14**
High maternal smoking and dietary inflammatory potential and low DASH	NA	1	10	**0.04**	**0.01**	**0.07**
Maternal glucose concentrations	NA	1	10	**0.04**	**0.01**	**0.07**
Middel cerebral artery pulsatility index	NA	1	6	**0.03**	**-0.03**	**0.09**
Gestational weight gain	NA	6	6	**0.02**	**0.00**	**0.04**
Assisted reproductive technology use	Natural conception	1	10	**0.01**	**-0.02**	**0.04**
Hypertensive disorders of pregnancy	No HDP	1	6	**0.01**	**-0.02**	**0.03**
Free thyroxine levels	NA	1	6	**-0.07**	**-0.10**	**-0.04**
Umbilical/Middle cerebral artery ratio	NA	1	6	**-0.06**	**-0.12**	**0.00**
Maternal weight during pregnancy	NA	3	2	**-0.04**	**-0.08**	**0.01**
Umbilical artery pulsatility index	NA	2	6	**-0.01**	**-0.03**	**0.02**
Haemoglobin	NA	1	6	**-0.01**	**-0.03**	**0.02**
Thyroid stimulating hormone levels	NA	1	6	**-0.01**	**-0.04**	**0.02**
Gestational weight gain	NA	1	2	**-0.01**	**-0.09**	**0.07**

[1] In the Cohen's d column, red highlights represent summary effect sizes with $|d|\geq 0.1$, and blue highlights represent summary effect sizes with $|d|<0.1$.

Table S7. Pooled effect sizes of predictors in maternal nutrition during pregnancy domain[1]

Predictors	Reference	Number of effect sizes	BMI time point	Cohen's d	ci.low	ci.hi
Maternal vomiting	Without daily vomiting	1	6	**0.26**	**0.23**	**0.28**
Vegetable, fish, and oil dietary pattern	Qartile 1	3	6	**0.1**	**0.06**	**0.14**
Milk intake	0-0.9 glass	5	10	**0.15**	**0.10**	**0.20**
Total n-6 PUFA	NA	1	6	**0.11**	**0.08**	**0.14**
DTA (22:4 n-6)	NA	1	2	**-0.1**	**-0.17**	**-0.04**
AA (20:4 n-6)	NA	1	2	**-0.11**	**-0.18**	**-0.05**
Folic acid supplement use	No	2	10	**-0.11**	**-0.23**	**0.01**
Maternal 25 (OH) D	NA	1	6	**-0.11**	**-0.13**	**-0.08**
Homocysteine	NA	2	10	**0**	**-0.03**	**0.02**
EDA (20:2 n-6)	NA	1	6	**0**	**-0.03**	**0.03**
Lemonade from concentrate intake	NA	1	6	**0**	**-0.03**	**0.03**
Soda intake	NA	1	6	**0**	**-0.04**	**0.03**
Total protein intake	Quantile 1	3	6	**0**	**-0.04**	**0.03**
AA (20:4 n-6)	NA	1	6	**0.09**	**0.06**	**0.12**
DTA (22:4 n-6)	NA	1	6	**0.08**	**0.05**	**0.11**
n-6 PUFA pattern	NA	1	6	**0.07**	**0.04**	**0.10**
Transferrin	NA	1	10	**0.07**	**0.04**	**0.10**
DGLA (20:3 n-6)	NA	1	6	**0.06**	**0.03**	**0.09**
Caffeine intake	< 2 units	3	10	**0.05**	**-0.15**	**0.25**
Animal protein intake	Quantile 1	3	6	**0.05**	**0.01**	**0.08**
DHA (22:6 n-3)	NA	1	2	**0.04**	**-0.03**	**0.11**
Nuts, soy, and high-fiber cereals dietary pattern	Qartile 1	3	6	**0.04**	**0.01**	**0.08**
EDA (20:2 n-6)	NA	1	2	**0.03**	**-0.04**	**0.10**
GLA (18:3 n-6)	NA	1	6	**0.03**	**0.00**	**0.06**
LA (18:2 n-6)	NA	1	2	**0.03**	**-0.04**	**0.10**
LA (18:2 n-6)	NA	1	6	**0.03**	**0.00**	**0.06**
Total n-6 PUFA	NA	1	2	**0.03**	**-0.04**	**0.10**
Cafeine intake	<2 units	6	6	**0.03**	**-0.03**	**0.10**

Table S7. Pooled effect sizes of predictors in maternal nutrition during pregnancy domain[1] *(continued)*

Predictors	Reference	Number of effect sizes	BMI time point	Cohen's d	ci.low	ci.hi
Active B12	NA	2	10	**0.03**	**0.00**	**0.05**
EPA (20:5 n-3)	NA	1	2	**0.02**	**-0.05**	**0.09**
Total n-3 PUFA	NA	1	2	**0.02**	**-0.05**	**0.09**
Margarine, snacks, and dietary pattern	Qartile 1	3	6	**0.02**	**-0.02**	**0.06**
Total B12	NA	2	10	**0.02**	**-0.01**	**0.04**
DGLA (20:3 n-6)	NA	1	2	**0.01**	**-0.06**	**0.08**
Fruit juice intake	NA	1	6	**0.01**	**-0.03**	**0.04**
Total sugar-containing beverage intake	NA	1	6	**0.01**	**-0.02**	**0.04**
Total n-3 PUFA	NA	1	6	**-0.09**	**-0.12**	**-0.06**
ALA (18:3 n-3)	NA	1	6	**-0.08**	**-0.11**	**-0.05**
DHA (22:6 n-3)	NA	1	6	**-0.08**	**-0.11**	**-0.05**
DPA (22:5 n-3)	NA	1	2	**-0.08**	**-0.15**	**-0.01**
EPA (20:5 n-3)	NA	1	6	**-0.08**	**-0.11**	**-0.05**
Trasferrin saturation	NA	1	10	**-0.08**	**-0.11**	**-0.05**
GLA (18:3 n-6)	NA	1	2	**-0.07**	**-0.14**	**0.00**
n-3 PUFA pattern	NA	1	6	**-0.06**	**-0.09**	**-0.03**
DPA (22:5 n-3)	NA	1	6	**-0.05**	**-0.08**	**-0.02**
Cord blood 25 (OH) D	NA	1	6	**-0.05**	**-0.08**	**-0.02**
Ferritin	NA	1	10	**-0.04**	**-0.07**	**-0.01**
Vegetable protein intake	Quantile 1	3	6	**-0.04**	**-0.08**	**-0.01**
ALA (18:3 n-3)	NA	1	2	**-0.03**	**-0.10**	**0.04**
Folate	NA	2	10	**-0.03**	**-0.06**	**0.00**
MUFA and SFA pattern	NA	1	6	**-0.02**	**-0.05**	**0.01**

[1] In the Cohen's d column, red highlights represent summary effect sizes with $|d|\geq 0.1$, and blue highlights represent summary effect sizes with $|d|<0.1$.

Table S8. Pooled effect sizes of predictors in genetics domain[1]

Predictors	Reference	Number of effect sizes	BMI time point	Cohen's d	ci.low	ci.hi
Hypothalamic expression and regulation genetic risk score	NA	1	6	**0.1**	**0.07**	**0.13**
rs7185735 (SAT)	NA	1	10	**0.12**	**0.08**	**0.16**
Adult WHR genetic risk score	NA	2	6	**0**	**-0.03**	**0.02**
Child BMI genetic risk score	NA	1	6	**0.09**	**0.06**	**0.12**
rs2123685 (SAT female)	NA	1	10	**0.09**	**0.05**	**0.13**
Adult BMI genetic risk score	NA	2	6	**0.08**	**0.01**	**0.14**
Membrane proteins genetic risk socre	NA	1	6	**0.08**	**0.05**	**0.11**
rs10182181 (ADCY3/POMC)	NA	1	4	**0.08**	**0.04**	**0.12**
Monogenic obesity/energy homeostasis genetic risk socre	NA	1	6	**0.07**	**0.04**	**0.10**
WNTSignaling genetic risk socre	NA	1	6	**0.06**	**0.03**	**0.09**
GR-gene (GR-9β)	NA	1	6	**0.06**	**0.03**	**0.09**
Muscle biology genetic risk socre	NA	1	6	**0.05**	**0.02**	**0.08**
Glucose homeostasis/diabetes genetic risk socre	NA	1	6	**0.05**	**0.02**	**0.08**
Immune system genetic risk socre	NA	1	6	**0.05**	**0.02**	**0.08**
Retinoic acid receptors genetic risk socre	NA	1	6	**0.05**	**0.02**	**0.08**
CyclicAMP genetic risk socre	NA	1	6	**0.05**	**0.02**	**0.08**
Child BMI genetic risk score	NA	1	4	**0.04**	**0.00**	**0.08**
Cell cycle genetic risk socre	NA	1	6	**0.04**	**0.01**	**0.07**
Limb development genetic risk socre	NA	1	6	**0.04**	**0.01**	**0.07**
Mitochondrail genetic risk socre	NA	1	6	**0.04**	**0.01**	**0.07**
Tumorigenesis genetic risk socre	NA	1	6	**0.04**	**0.01**	**0.07**
GR-gene (BclI)	NA	1	6	**0.04**	**0.01**	**0.07**
rs11030104 (BDNF)	NA	1	4	**0.04**	**0.00**	**0.08**
Adult BMI genetic risk score	NA	1	10	**0.03**	**-0.01**	**0.07**
Child BMI genetic risk score	NA	1	10	**0.03**	**-0.01**	**0.07**

Table S8. Pooled effect sizes of predictors in genetics domain[1] *(continued)*

Predictors	Reference	Number of effect sizes	BMI time point	Cohen's d	ci.low	ci.hi
Apoptosis genetic risk socre	NA	1	6	**0.03**	**0.00**	**0.06**
Child FTO	AT	2	4	**0.03**	**-0.02**	**0.08**
JAK genetic risk socre	NA	1	6	**0.03**	**0.00**	**0.06**
MAPK1/extracellular signal-regulated kinases genetic risk socre	NA	1	6	**0.03**	**0.00**	**0.06**
Maternal BMI alleles score	NA	1	6	**0.02**	**-0.02**	**0.06**
Hormone metabolism/regulation genetic risk socre	NA	1	6	**0.02**	**-0.01**	**0.05**
PROP taster status	Non-taster	1	6	**0.02**	**-0.01**	**0.04**
Lipid biosynthesis and metabolism genetic risk score	NA	1	6	**0.02**	**-0.01**	**0.05**
Neuronal developmental processes genetic risk score	NA	1	6	**0.02**	**-0.01**	**0.05**
Neuronal expression genetic risk score	NA	1	6	**0.02**	**-0.01**	**0.05**
rs2842895 (VAT and VATadjBMI)	NA	1	10	**0.02**	**-0.02**	**0.06**
Bone development genetic risk score	NA	1	6	**0.02**	**-0.01**	**0.05**
Adult BMI genetic risk score	NA	1	4	**0.01**	**-0.03**	**0.05**
Eye-related genetic risk socre	NA	1	6	**0.01**	**-0.02**	**0.04**
Purine/pyrimidine cycle genetic risk socre	NA	1	6	**0.01**	**-0.02**	**0.04**
INS VNTR	I/I	2	2	**0.01**	**-0.07**	**0.10**
Neurotransmission genetic risk score	NA	1	6	**0.01**	**-0.02**	**0.04**
rs10733682 (LMX1B)	NA	1	4	**0.01**	**-0.03**	**0.05**
rs4256980 (TUB)	NA	1	4	**-0.07**	**-0.11**	**-0.03**
rs10060123 (VAT and VATadjBMI female)	NA	1	10	**-0.06**	**-0.10**	**-0.02**
GR-gene (ER22/23EK)	NA	1	6	**-0.05**	**-0.08**	**-0.02**
rs10938397 (GNPDA2)	NA	1	4	**-0.05**	**-0.09**	**-0.01**

Table S8. Pooled effect sizes of predictors in genetics domain[1] *(continued)*

Predictors	Reference	Number of effect sizes	BMI time point	Cohen's d	ci.low	ci.hi
rs6567160 (MC4R)	NA	1	4	**-0.05**	**-0.09**	**-0.01**
IGF1	Homozygous	2	2	**-0.04**	**-0.12**	**0.04**
GR-gene (N363S)	NA	1	6	**-0.04**	**-0.07**	**-0.01**
Adult pericardial fat genetic risk score	NA	1	10	**-0.03**	**-0.07**	**0.01**
Notch signaling genetic risk socre	NA	1	6	**-0.03**	**-0.06**	**0.00**
rs1558902 (FTO)	NA	1	4	**-0.03**	**-0.07**	**0.01**
rs3736485 (SCG3)	NA	1	4	**-0.02**	**-0.06**	**0.02**
rs3888190 (SH2B1)	NA	1	4	**-0.02**	**-0.06**	**0.02**
rs7164727 (BBS4)	NA	1	4	**-0.02**	**-0.06**	**0.02**
Adult WHR genetic risk score	NA	1	10	**-0.01**	**-0.05**	**0.03**
Adult fatty liver genetic risk score	NA	1	10	**-0.01**	**-0.05**	**0.03**
VAT/SAT ratio genetic risk score	NA	1	10	**-0.01**	**-0.05**	**0.03**
Nuclear trafficking genetic risk socre	NA	1	6	**-0.01**	**-0.04**	**0.02**
Endocytosis/exocytosis genetic risk socre	NA	1	6	**-0.01**	**-0.04**	**0.02**
Ubiquitin pathways genetic risk socre	NA	1	6	**-0.01**	**-0.04**	**0.02**

[1] In the Cohen's d column, red highlights represent summary effect sizes with $|d| \geq 0.1$, and blue highlights represent summary effect sizes with $|d| < 0.1$.

Table S9. Pooled effect sizes of predictors in maternal mental health and parenting domain[1]

Predictors	Reference	Number of effect sizes	BMI time point	Cohen's d	ci.low	ci.hi
Distress	No stress	1	10	0.42	**0.39**	**0.46**
Timing of first solid foods	>= 5 months	2	10	0.22	**0.08**	**0.37**
Timing of first solid foods	>= 5 months	4	6	0.16	**0.10**	**0.21**
Breastfeeding	Ever breastfed	1	10	0.14	**0.11**	**0.17**
Depression	No	3	3	0.13	**0.08**	**0.19**
Restriction	NA	2	10	0.12	**-0.09**	**0.32**
Pressure to eat	NA	1	6	-0.13	**-0.17**	**-0.09**
Pressure to eat	NA	2	4	-0.12	**-0.27**	**0.03**
Family stress	NA	1	4	0	**-0.03**	**0.03**
Using food as reward	NA	1	10	0	**-0.03**	**0.03**
Use of food to soothe	Never	2	10	0.09	**-0.09**	**0.27**
Restriction	NA	1	4	0.09	**0.06**	**0.13**
Restriction	NA	1	6	0.08	**0.04**	**0.12**
Breastfeeding	Ever breastfed	2	6	0.07	**-0.01**	**0.15**
Hostility	NA	1	4	0.06	**0.03**	**0.09**
Breastfeeding exclusivity	Exclusive until 4 months of age	1	10	0.05	**0.02**	**0.08**
Duration of breastfeeding	>= 6 months	3	10	0.05	**-0.02**	**0.11**
Breastfeeding exclusivity	Exclusive until 4 months of age	2	6	0.04	**0.00**	**0.07**
Depression	NA	1	4	0.04	**0.01**	**0.06**
Anxiety	NA	1	4	0.03	**0.01**	**0.06**
Duration of breastfeeding	>= 6 months	6	6	0.02	**-0.02**	**0.06**
Monitoring	NA	1	6	-0.07	**-0.10**	**-0.04**
Monitoring	NA	1	4	-0.01	**-0.04**	**0.02**

[1] In the Cohen's d column, red highlights represent summary effect sizes with $|d| \geq 0.1$, and blue highlights represent summary effect sizes with $|d| < 0.1$.

Table S10. Pooled effect sizes of predictors in early-life weight and weight gain domain[1]

Predictors	Reference	Number of effect sizes	BMI time point	Cohen's d	ci.low	ci.hi
BMI-2y	NA	1	6	**0.58**	**0.50**	**0.65**
BMI at adiposity peak	NA	1	6	**0.52**	**0.50**	**0.55**
Peak weight velocity	NA	1	6	**0.41**	**0.38**	**0.44**
Weight strata for gestational age combined with catch up/down growth	Appropriate GA, no catch up/catch down	6	4	**0.3**	**0.20**	**0.39**
BMI-1.5mo	NA	1	6	**0.22**	**0.15**	**0.30**
Weight gain infancy	NA	3	6	**0.22**	**0.14**	**0.29**
Total subcutaneous fat mass	NA	2	6	**0.21**	**-0.04**	**0.45**
BMI gain-infancy	NA	2	6	**0.16**	**0.11**	**0.21**
Central-to-total fat mass ratio	NA	3	6	**0.12**	**0.04**	**0.20**
Fetal growth restriction	NA	1	6	**-0.79**	**-0.85**	**-0.74**
Total subcutaneous fat mass	NA	3	10	**0.09**	**0.00**	**0.19**
Weight gain fetal	NA	2	6	**0.07**	**0.04**	**0.09**
Central-to-total fat mass ratio	NA	3	10	**0.06**	**-0.01**	**0.13**
Fetal and infant growth	Fetal normal - infant normal	8	6	**0.05**	**-0.29**	**0.38**
Abdominal circumference	NA	1	6	**0.04**	**0.01**	**0.07**
Length gain fetal	NA	2	6	**0.04**	**-0.02**	**0.11**
Age at adiposity peak	NA	1	6	**0.03**	**0.00**	**0.06**
Length gain infancy	NA	3	6	**0.03**	**0.00**	**0.07**
Weight status for gestational age	Appropriate GA	2	10	**0.03**	**-0.09**	**0.16**

[1] In the Cohen's d column, red highlights represent summary effect sizes with $|d|\geq0.1$, and blue highlights represent summary effect sizes with $|d|<0.1$.

Table S11. Pooled effect sizes of predictors in infant and childhood nutrition domain[1]

Predictors	Reference	Number of effect sizes	BMI time point	Cohen's d	ci.low	ci.hi
High FMI dietary pattern	NA	1	6	**0.14**	**0.10**	**0.18**
Plant protein intake	NA	1	10	**0.14**	**0.11**	**0.17**
Animal protein intake	NA	1	10	**0.13**	**0.10**	**0.16**
Total fat intake	NA	1	6	**0**	**-0.04**	**0.04**
High FFMI dietary pattern	NA	1	6	**0**	**-0.04**	**0.04**
Total monosaccharide and disaccharide intake (10g/d)	NA	1	10	**0**	**-0.03**	**0.03**
Total protein intake	NA	1	6	**0.08**	**0.04**	**0.11**
Total protein intake	NA	1	10	**0.08**	**0.05**	**0.11**
Animal protein intake	NA	1	10	**0.06**	**0.03**	**0.10**
Total protein intake	NA	1	10	**0.06**	**0.03**	**0.10**
Diet quality score	NA	1	6	**0.05**	**0.01**	**0.09**
Animal protein intake	NA	1	6	**0.05**	**0.01**	**0.09**
Health-conscious dietary patterns	NA	1	6	**0.04**	**0.00**	**0.08**
Western dietary pattern	NA	1	6	**0.04**	**0.00**	**0.08**
Folate	Tertile 1 (<80.9)	2	6	**0.04**	**0.00**	**0.07**
Sugar cotaining beverage intake	Low intake (<3 servings/w)	4	6	**0.03**	**-0.02**	**0.08**
Vegetable protein intake	NA	1	6	**0.03**	**0.00**	**0.07**
SFAs	NA	1	6	**0.02**	**-0.01**	**0.06**
Diet quality score	NA	2	10	**0.01**	**-0.06**	**0.09**
Vegetable protein intake	NA	1	10	**0.01**	**-0.02**	**0.04**
Lutein intake	NA	1	6	**0.01**	**-0.03**	**0.05**
Methionine	Tertile 1 (<681.5)	2	6	**0.01**	**-0.02**	**0.05**
Vitamin B12	Tertile 1 (<2.2)	2	6	**0.01**	**-0.02**	**0.05**
Vitamin B6	Tertile 1 (<1.1)	2	6	**0.01**	**-0.02**	**0.05**
Folic acid	Tertile 1 (<304.3)	2	6	**-0.04**	**-0.07**	**0.00**
Folate equivalent	Tertile 1 (<167.8)	2	6	**-0.03**	**-0.06**	**0.01**
PUFAs	NA	1	6	**-0.02**	**-0.05**	**0.02**
Total carbohydrate intake (10g/d)	NA	1	10	**-0.01**	**-0.04**	**0.02**
MUFAs	NA	1	6	**-0.01**	**-0.04**	**0.03**
Total polysacharide intake (10g/d)	NA	1	10	**-0.01**	**-0.04**	**0.02**

[1] In the Cohen's d column, red highlights represent summary effect sizes with $|d|\geq 0.1$, and blue highlights represent summary effect sizes with $|d|<0.1$.

Table S12. Pooled effect sizes of predictors in child behaviors domain[1]

Predictors	Reference	Number of effect sizes	BMI time point	Cohen's d	ci.low	ci.hi
Breakfast skipping	No	2	6	0.32	**0.20**	**0.44**
Food responsiveness	NA	4	10	0.26	**0.13**	**0.39**
Food responsiveness	NA	2	4	0.22	**0.20**	**0.25**
Emotional overeating	NA	4	10	0.1	**0.04**	**0.16**
Lunch skipping	No	2	6	0.19	**0.07**	**0.32**
TV vieweing	< 2 hrs/day	1	6	0.19	**0.17**	**0.22**
Enjoyment of food	NA	2	4	0.16	**0.14**	**0.19**
Enjoyment of food	NA	2	10	0.15	**0.03**	**0.27**
Dinner skipping	No	2	6	0.13	**-0.01**	**0.27**
Computer game	< 1 hr/day	1	6	0.12	**0.09**	**0.15**
Outdoor play	>= 1 hr/day	1	6	0.12	**0.09**	**0.15**
Set-shifting	NA	2	10	-0.35	**-0.91**	**0.21**
Satiety responsiveness	NA	2	4	-0.24	**-0.27**	**-0.22**
Satiety responsiveness	NA	2	10	-0.22	**-0.35**	**-0.09**
Emotional undereating	NA	2	4	-0.1	**-0.13**	**-0.08**
ADHD traits	<80th percentile	1	14	0.05	**0.02**	**0.09**
Externalizing	NA	3	6	0.04	**0.02**	**0.06**
Sports participation	Yes	1	6	0.04	**0.01**	**0.07**
Emotional overeating	NA	2	4	0.03	**0.01**	**0.05**
ADHD	No ADHD diagnosis	2	10	0.03	**-0.06**	**0.11**
Aggressive behavior	NA	2	10	0.02	**-0.01**	**0.04**
Internalizing	NA	3	6	0.02	**-0.03**	**0.07**
Sendentary behavior	NA	1	2	0.02	**-0.09**	**0.13**
Desire to drink	NA	2	4	0.01	**-0.01**	**0.03**
Active transport	>= 5 days/week	1	6	-0.09	**-0.12**	**-0.06**
Fussiness	NA	2	4	-0.08	**-0.10**	**-0.05**
Fussy eating profile	Non-fussy eater	1	6	-0.08	**-0.11**	**-0.05**
Sleep duration	NA	4	6	-0.05	**-0.07**	**-0.04**
Internalizing	NA	2	4	-0.04	**-0.06**	**-0.01**
Autistic traits	<80th percentile	1	14	-0.04	**-0.07**	**0.00**
Externalizing	NA	2	4	-0.03	**-0.05**	**-0.01**
Moderate-to-vigorous physical activity	NA	1	2	-0.03	**-0.14**	**0.08**
Movement counts/minute	NA	1	2	-0.03	**-0.14**	**0.08**

[1] In the Cohen's d column, red highlights represent summary effect sizes with $|d| \geq 0.1$, and blue highlights represent summary effect sizes with $|d| < 0.1$.

Table S13. Pooled effect sizes of predictors in biological factors domain[1]

Predictors	Reference	Number of effect sizes	BMI time point	Cohen's d	ci.low	ci.hi
Cortisol	NA	1	6	**0.6**	**0.57**	**0.64**
Cortisol	0.14-0.73 pg/mg	4	10	**0.27**	**0.14**	**0.40**
Cortisone	NA	1	6	**0.23**	**0.20**	**0.27**
Anti-tissue Transglutaminase concentrations	Negative anti-tTG (<7 U/mL)	2	6	**0.04**	**-0.01**	**0.08**

[1] In the Cohen's d column, red highlights represent summary effect sizes with $|d| \geq 0.1$, and blue highlights represent summary effect sizes with $|d| < 0.1$.

Table S14. Descriptive statistics for all predictors included in multiple regression models (n = 5686)

Predictors	Time of assessment	Median [IQR] or %
Sociodemographic factors		
Child ethnic background	Baseline	
Dutch		60.2
Non-Dutch Western		9.1
Non-Dutch Non-Western		30.7
Maternal education	Baseline	
Low		20.3
Middle		30.7
High		49.0
Paternal education	Baseline	
Low		25.7
Middle		27.2
High		47.1
Maternal education	5 years	
Low		12.7
Middle		31.2
High		56.1
Paternal education	5 years	
Low		18.0
Middle		27.9
High		54.0
Preconception and prenatal parental health		
Maternal BMI, kg/m^2	Before pregnancy	22.7 [20.8, 25.2]
Paternal BMI, kg/m^2	Baseline	25.0 [23.0, 27.2]
Maternal weight	Before pregnancy	64.0 [58.0, 72.0]
Maternal smoking	Baseline	
No		76.3
Until pregnancy		8.7
Continued		15.0

Maternal nutrition during pregnancy		
Total n-6 polyunsaturated fatty acids percentage by weight of total sum of fatty acids, %	During pregnancy	36.5 [34.9, 38.2]
Vitamin D concentration, 25(OH)D, nmol/L	During pregnancy	53.0 [30.4, 79.0]
Folic acid use	Baseline	
No		23.4
Start first trimester		31.9
Start periconceptional		44.7
Milk intake	During pregnancy	
<1 glasses		34.5
1 – <2 glasses		26.3
2 – <3 glasses		23.5
≥3 glasses		15.7
Maternal mental health and parenting		
Pressure to eat	4 years	13.0 [10.0, 16.0]
Restriction	10 years	15.0 [10.0, 19.0]
The timing of the introduction of solid foods	During infancy	
<4 months		21.8
4-<5 months		56.9
≥5 months		21.3
Breastfeeding duration	During infancy	
<2 months		29.5
2-<4 months		22.1
4-<6 months		12.9
≥6 months		35.5
Early-life weight and weight gain		

Weight gain during infancy, grams	0 to 6 months	4385.0 [3850.0, 4960.0]
Weight gain during infancy, grams	6 to 12 months	1730.0 [1370.0, 2130.0]
Weight gain during infancy, grams	12 to 24 months	3190.0 [2670.0, 3770.0]
zBMI, SD	1.5 months	0.5 [-0.2, 1.1]
zBMI, SD	2 years	0.2 [-0.4, 0.9]
BMI at estimated adiposity peak, kg/m^2	-	17.6 [17.0, 18.1]
Infant and childhood nutrition		
High fat-mass dietary pattern, units of component score	1 year	0.0 [-0.5, 0.7]
Animal and plant protein intake, grams	1 year	40.4 [32.8, 48.4]
Child behaviors		
Enjoyment of food, units of sum score	4 years	14.0 [12.0, 16.0]
Emotional overeating, units of sum score	10 years	4.0 [4.0, 8.0]
Food responsiveness, units of sum score	10 years	8.0 [6.0, 11.0]
Satiety responsiveness, units of sum score	10 years	23.0 [19.0, 27.0]
Breakfast skipping	6 years	
Yes		6.3
No		93.7
Television viewing	6 years	
<2 hours/day		33.3
≥2 hours/day		66.7
Outdoor play	6 years	
<1 hours/day		13.9

≥1 hours/day		86.1
Set-shifting	4 years	46.0 [42.0, 54.0]
Genetic variants		
BMI polygenic risk score (based on 25 SNPs)	6 years	25.0 [23.3, 26.7]

Table S15. Associations between key predictors and zBMI or overweight and obesity in multiple regression models including child BMI polygenic risk score (N = 3584)

Predictors (IQR or reference category)	Time of assessment	zBMI	Overweight or obesity
		β (95% CI)	*OR* (95% CI)
Sociodemographic factors			
Child ethnic background (ref: Dutch)	Baseline		
Non-Dutch Western		0.12 (0.01, 0.22)	1.75 (1.14, 2.68)
Non-Dutch Non-Western		0.05 (-0.06, 0.16)	1.04 (0.68, 1.58)
Maternal education (ref: high)	Baseline		
Middle		-	1.61 (1.17, 2.22)
Low		-	1.77 (1.19, 2.62)
Paternal education (ref: high)	Baseline		
Middle		-	1.45 (1.05, 2.02)
Low		-	1.26 (0.85, 1.85)
Maternal education (ref: high)	5 years		
Middle		0.12 (0.05, 0.19)	-
Low		0.19 (0.08, 0.3)	-
Paternal education (ref: high)	5 years		
Middle		0.09 (0.01, 0.16)	-
Low		0.08 (-0.02, 0.18)	-
Preconception and prenatal parental health			
Maternal BMI (4.4 kg/m^2)	Before pregnancy	0.18 (0.14, 0.21)	1.53 (1.34, 1.75)
Paternal BMI (4.2 kg/m^2)	Baseline	0.17 (0.14, 0.21)	1.51 (1.27, 1.79)
Maternal smoking (ref: no)	Baseline		
Until pregnancy		0.02 (-0.09, 0.12)	0.97 (0.63, 1.49)
Continued		0.12 (0.04, 0.2)	1.31 (0.95, 1.8)
Maternal nutrition during pregnancy			
Total n-6 polyunsaturated fatty acids percentage by weight of total sum of fatty acids (3.3%)	During pregnancy	-	0.99 (0.82, 1.19)
Vitamin D status, 25(OH)D (48.4 nmol/L)	During pregnancy	-0.05 (-0.1, 0)	0.88 (0.7, 1.11)
Folic acid supplement use (ref: start periconceptional)	During pregnancy		
Start first 10 weeks		0.06 (0, 0.13)	1.26 (0.94, 1.7)
Never		0.04 (-0.06, 0.14)	1.3 (0.89, 1.89)

Milk intake (ref: <1 glasses)	During pregnancy		
1 – <2 glasses		-0.01 (-0.09, 0.06)	0.85 (0.6, 1.19)
2 – <3 glasses		0.01 (-0.07, 0.09)	0.79 (0.54, 1.15)
≥3 glasses		0.06 (-0.03, 0.15)	1.13 (0.75, 1.69)
Maternal mental health and parenting			
The timing of the introduction of solid foods (ref: ≥5 months)	During infancy		
4-<5 months		-	1.21 (0.83, 1.77)
<4 months		-	1.19 (0.73, 1.92)
Breastfeeding duration (ref: ≥6 months)	During infancy		
4-<6 months		-	1.22 (0.77, 1.95)
2-<4 months		-	1.02 (0.7, 1.49)
<2 months		-	0.91 (0.63, 1.31)
Pressure to eat (6 unit of sum score)	4 years	-0.03 (-0.08, 0.01)	-
Restriction (9 units of sum score)	10 years	0.13 (0.08, 0.18)	1.48 (1.18, 1.86)
Early-life weight and weight gain			
Weight gain during infancy (1110 g)	0 to 6 months	0.05 (0, 0.1)	-
Weight gain during infancy (760 g)	6 to 12 months	0.07 (0.03, 0.11)	1.18 (1.01, 1.37)
Weight gain during infancy (1100 g)	12 to 24 months	0.09 (0.05, 0.13)	1.28 (1.06, 1.53)
zBMI (1.3 SD)	1.5 months	0.09 (0.04, 0.15)	1.34 (1.08, 1.67)
zBMI (1.4 SD)	2 years	0.28 (0.22, 0.34)	1.99 (1.62, 2.44)
BMI at estimated adiposity peak (1.1 kg/m^2)	-	0.17 (0.11, 0.23)	-
Infant and childhood nutrition			
High fat-mass dietary pattern (1.2 unit of component scores)	1 year	0.05 (0, 0.1)	1.12 (0.94, 1.34)
Animal and plant protein intake (15.8 g)	1 year	0.03 (-0.02, 0.07)	1.11 (0.95, 1.31)
Child behaviors			
Enjoyment of food (4 units of sum score)	4 years	-0.03 (-0.08, 0.01)	-
Emotional overeating (4 units of sum score)	10 years	-	1.1 (0.91, 1.34)
Food responsiveness (5 units of sum score)	10 years	0.24 (0.2, 0.28)	2.25 (1.9, 2.68)
Satiety responsiveness (8 units of sum score)	10 years	-0.17 (-0.21, -0.13)	0.73 (0.61, 0.88)
Breakfast skipping (ref: no)	6 years	0.22 (0.1, 0.35)	1.81 (1.13, 2.9)
Television viewing (ref: <2 hours/day)	6 years	0.06 (0, 0.12)	-
Outdoor play (ref: >1 hour/day)	6 years	-	0.87 (0.59, 1.28)
Set-shifting (10 units of sum score)	4 years	-0.05 (-0.09, -0.01)	0.88 (0.73, 1.07)

Genetic variants			
Child BMI polygenic risk scores (3.4 additional average risk allele)		0.14 (0.1, 0.18)	1.45 (1.23, 1.7)
**Explained variance (R^2)**		48.7% (46.1%, 51.2%)	35.2%[a]

Predictors selected in at least 10% DSA models were identified as key predictors. Since the DSA algorithm was performed separately for zBMI and weight status, a set of key predictors was identified for each outcome. These key predictors were then included simultaneously in the multiple linear regression model for zBMI and the logistic regression model for weight status, respectively. Predictors not modeled in a given outcome were marked with a '-' in the table.

For continuous variables, β represent differences in zBMI per interquartile range increase in predictors; for categorical variables, β represent differences of zBMI in comparison category vs. reference categories. For continuous variables, *OR* represent differences in odds of overweight and obesity per interquartile range increase in predictors; for categorical variables, *OR* represent differences of odds of overweight and obesity in comparison category vs. reference categories. IQR and reference categories are indicated in the bracket for predictors. Models include all key predictors selected by the DSA algorithm and child BMI polygenic risk scores.

[a] McFadden's pseudo R^2 was calculated for explained variance in the odds of being overweight and obesity at the age of 10 years.

Table S16. Associations between key predictors and zBMI or overweight and obesity in multiple regression models stratified by child sex (N = 5686)

Predictors (IQR or reference category)	Time of assessment	zBMI		Overweight or obesity	
		β (95% CI)		*OR* (95% CI)	
		Girls (n = 2866)	Boys (n = 2820)	Girls (n = 2866)	Boys (n = 2820)
Sociodemographic factors					
Child ethnic background (ref: Dutch)	Baseline				
Non-Dutch Western		0.16 (0.05, 0.26)	0.11 (-0.01, 0.22)	1.74 (1.1, 2.76)	1.82 (1.11, 2.99)
Non-Dutch Non-Western		0.16 (0.07, 0.25)	0.06 (-0.04, 0.16)	1.92 (1.33, 2.78)	1.52 (1.04, 2.23)
Maternal education (ref: high)	Baseline				
Middle		-	-	1.93 (1.34, 2.78)	1.1 (0.77, 1.58)
Low		-	-	1.86 (1.19, 2.91)	1.5 (0.94, 2.37)
Paternal education (ref: high)	Baseline				
Middle		-	-	1.53 (1.04, 2.24)	1.48 (1.02, 2.16)
Low		-	-	1.54 (0.98, 2.43)	1.43 (0.94, 2.17)
Maternal education (ref: high)	5 years				
Middle		0.12 (0.04, 0.2)	0.11 (0.03, 0.19)	-	-
Low		0.18 (0.06, 0.31)	0.21 (0.09, 0.34)	-	-
Paternal education (ref: high)	5 years				
Middle		0.08 (-0.01, 0.16)	0.1 (0.01, 0.18)	-	-
Low		0.12 (0.02, 0.23)	0.06 (-0.06, 0.18)	-	-
Preconception and prenatal parental health					
Maternal BMI (4.4 kg/m^2)	Before pregnancy	0.19 (0.16, 0.23)	0.15 (0.1, 0.19)	1.55 (1.34, 1.8)	1.39 (1.2, 1.61)
Paternal BMI (4.2 kg/m^2)	Baseline	0.16 (0.12, 0.2)	0.18 (0.13, 0.23)	1.5 (1.25, 1.8)	1.47 (1.22, 1.77)
Maternal smoking (ref: no)	Baseline				
Until pregnancy		0.04 (-0.07, 0.15)	0.01 (-0.11, 0.12)	1.5 (0.96, 2.33)	0.74 (0.43, 1.27)
Continued		0.19 (0.09, 0.28)	0.04 (-0.05, 0.13)	1.47 (1.01, 2.13)	1.15 (0.80, 1.64)
Maternal nutrition during pregnancy					
Total n-6 polyunsaturated fatty acids percentage by weight of total sum of fatty acids (3.3%)	During pregnancy	-	-	1.15 (0.94, 1.42)	1.21 (0.97, 1.5)
Vitamin D status, 25(OH)D (48.4 nmol/L)	During pregnancy	-0.05 (-0.11, 0)	-0.11 (-0.18, -0.04)	0.7 (0.53, 0.93)	0.84 (0.63, 1.11)
Folic acid supplement use (ref: start periconceptional)	During pregnancy				
Start first 10 weeks		0.07 (0, 0.14)	0.08 (-0.01, 0.16)	1.13 (0.8, 1.59)	1.46 (1, 2.13)

Never		0.06 (-0.05, 0.18)	0.04 (-0.08, 0.16)	1.2 (0.76, 1.9)	1.34 (0.84, 2.15)
Milk intake (ref: <1 glasses)	During pregnancy				
1 – <2 glasses		0.02 (-0.06, 0.1)	0 (-0.08, 0.08)	1.07 (0.74, 1.57)	0.78 (0.55, 1.11)
2 – <3 glasses		0 (-0.09, 0.08)	0.03 (-0.07, 0.12)	0.89 (0.6, 1.34)	0.79 (0.51, 1.22)
≥3 glasses		0.06 (-0.05, 0.16)	0.05 (-0.06, 0.15)	1.23 (0.76, 1.99)	1.03 (0.67, 1.56)
Maternal mental health and parenting					
The timing of the introduction of solid foods (ref: ≥5 months)	During infancy				
4-<5 months		-	-	1.18 (0.77, 1.8)	1.2 (0.75, 1.93)
<4 months		-	-	1.3 (0.78, 2.18)	1.18 (0.67, 2.07)
Breastfeeding duration (ref: ≥6 months)	During infancy				
4-<6 months		-	-	1.39 (0.85, 2.27)	0.87 (0.52, 1.47)
2-<4 months		-	-	1.24 (0.79, 1.92)	0.78 (0.5, 1.22)
<2 months		-	-	0.97 (0.64, 1.45)	0.86 (0.54, 1.36)
Pressure to eat (6 unit of sum score)	4 years	-0.04 (-0.09, 0)	-0.05 (-0.1, 0)	-	-
Restriction (9 units of sum score)	10 years	0.18 (0.13, 0.24)	0.17 (0.11, 0.23)	1.53 (1.21, 1.93)	1.61 (1.24, 2.09)
Early-life weight and weight gain					
Weight gain during infancy (1110 g)	0 to 6 months	0.08 (0.03, 0.14)	0.06 (0, 0.11)	-	-
Weight gain during infancy (760 g)	6 to 12 months	0.08 (0.03, 0.12)	0.06 (0.02, 0.1)	1.27 (1.04, 1.54)	1.14 (0.96, 1.35)
Weight gain during infancy (1100 g)	12 to 24 months	0.11 (0.06, 0.15)	0.1 (0.05, 0.15)	1.36 (1.13, 1.62)	1.21 (0.99, 1.48)
zBMI (1.3 SD)	1.5 months	0.08 (0.03, 0.14)	0.07 (0.01, 0.13)	1.4 (1.11, 1.75)	1.23 (0.98, 1.54)
zBMI (1.4 SD)	2 years	0.27 (0.19, 0.34)	0.29 (0.23, 0.36)	1.92 (1.5, 2.46)	1.83 (1.46, 2.29)
BMI at estimated adiposity peak (1.1 kg/m^2)	-	0.16 (0.09, 0.24)	0.19 (0.12, 0.26)	-	-
Infant and childhood nutrition					
High fat-mass dietary pattern (1.2 unit of component scores)	1 year	0.05 (0.01, 0.1)	0.05 (-0.01, 0.11)	1.15 (0.91, 1.45)	1.13 (0.94, 1.37)
Animal and plant protein intake (15.8 g)	1 year	0.03 (-0.02, 0.08)	0.02 (-0.03, 0.07)	1.09 (0.91, 1.31)	1.09 (0.9, 1.33)
Child behaviors					
Enjoyment of food (4 units of sum score)	4 years	-0.03 (-0.09, 0.02)	-0.06 (-0.12, 0.00)	-	-
Emotional overeating (4 units of sum score)	10 years	-	-	1.11 (0.91, 1.35)	1.15 (0.93, 1.42)
Food responsiveness (5 units of sum score)	10 years	0.22 (0.18, 0.26)	0.27 (0.22, 0.32)	2.09 (1.77, 2.48)	2.24 (1.88, 2.68)
Satiety responsiveness (8 units of sum score)	10 years	-0.19 (-0.24, -0.15)	-0.17 (-0.22, -0.12)	0.57 (0.46, 0.71)	0.71 (0.58, 0.87)
Breakfast skipping (ref: no)	6 years	0.19 (0.05, 0.32)	0.25 (0.11, 0.39)	1.86 (1.13, 3.07)	1.69 (1.04, 2.72)
Television viewing (ref: <2 hours/day)	6 years	0.05 (-0.02, 0.12)	0.1 (0.02, 0.18)	-	-
Outdoor play (ref: >1 hour/day)	6 years	-	-	0.81 (0.53, 1.25)	1.02 (0.68, 1.53)

Set-shifting (10 units of sum score)	4 years	-0.1 (-0.14, -0.05)	-0.03 (-0.08, 0.01)	0.72 (0.57, 0.91)	1.02 (0.83, 1.26)
**Explained variance (R^2)**		52.5% (49.6%, 55.3%)	46.0% (42.8%, 48.9%)	38.4%[a]	30.7 %[a]

Predictors selected in at least 10% DSA models were identified as key predictors. Since the DSA algorithm was performed separately for zBMI and weight status, a set of key predictors was identified for each outcome. These key predictors were then included simultaneously in the multiple linear regression model for zBMI and the logistic regression model for weight status, respectively. Predictors not modeled in a given outcome were marked with a '-' in the table.

For continuous variables, β represent differences in zBMI per interquartile range increase in predictors; for categorical variables, β represents differences of zBMI in comparison category vs. reference categories. For continuous variables, *OR* represent differences in odds of overweight and obesity per interquartile range increase in predictors; for categorical variables, *OR* represent differences of odds of overweight and obesity in comparison category vs. reference categories. IQR and reference categories are indicated in the bracket for predictors. Models include all key predictors selected by the DSA algorithm.

[a] McFadden's pseudo R^2 was calculated for explained variance in the odds of being overweight and obesity at the age of 10 years.

Table S17. Associations between key predictors and zBMI or overweight and obesity in multiple regression models stratified by child ethnic background (N = 5686)

Predictors (IQR or reference category)	Time of assessment	zBMI		Overweight or obesity	
		β (95% CI)		*OR* (95% CI)	
		Dutch (n = 3417)	Non-Dutch (n = 2269)	Dutch (n = 3417)	Non-Dutch (n = 2269)
Sociodemographic factors					
Maternal education (ref: high)	Baseline	-	-		
Middle		-	-	1.41 (0.99, 2.01)	1.40 (0.98, 2.01)
Low				2.01 (1.22, 3.29)	1.33 (0.87, 2.03)
Paternal education (ref: high)	Baseline	-	-		
Middle		-	-	2.03 (1.4, 2.92)	1.10 (0.75, 1.62)
Low				1.99 (1.22, 3.25)	1.18 (0.79, 1.77)
Maternal education (ref: high)	5 years				
Middle		0.11 (0.04, 0.18)	0.10 (0.00, 0.20)	-	-
Low		0.32 (0.18, 0.45)	0.10 (-0.03, 0.24)	-	-
Paternal education (ref: high)	5 years				
Middle		0.12 (0.05, 0.19)	0.03 (-0.08, 0.14)	-	-
Low		0.08 (-0.03, 0.18)	0.09 (-0.05, 0.23)	-	-
Preconception and prenatal parental health					
Maternal BMI (4.4 kg/m^2)	Before pregnancy	0.18 (0.15, 0.21)	0.15 (0.11, 0.20)	1.57 (1.36, 1.81)	1.38 (1.19, 1.59)
Paternal BMI (4.2 kg/m^2)	Baseline	0.19 (0.15, 0.23)	0.15 (0.09, 0.21)	1.54 (1.29, 1.84)	1.43 (1.17, 1.74)
Maternal smoking (ref: no)	Baseline				
Until pregnancy		0.03 (-0.06, 0.13)	0.02 (-0.13, 0.17)	1.25 (0.79, 1.98)	1.05 (0.62, 1.78)
Continued		0.16 (0.08, 0.24)	0.04 (-0.07, 0.15)	1.53 (1.04, 2.26)	1.10 (0.78, 1.55)
Maternal nutrition during pregnancy					
Total n-6 polyunsaturated fatty acids percentage by weight of total sum of fatty acids (3.3%)	During pregnancy	-	-	1.18 (0.92, 1.50)	1.21 (0.99, 1.49)
Vitamin D status, 25(OH)D (48.4 nmol/L)	During pregnancy	-0.07 (-0.12, -0.02)	-0.11 (-0.19, -0.03)	0.75 (0.57, 0.97)	0.72 (0.55, 0.95)
Folic acid supplement use (ref: start periconceptional)	During pregnancy				
Start first 10 weeks		0.06 (0.00, 0.12)	0.11 (-0.01, 0.23)	1.33 (0.96, 1.83)	1.19 (0.78, 1.81)
Never		0.00 (-0.10, 0.11)	0.11 (-0.03, 0.24)	1.13 (0.68, 1.88)	1.36 (0.90, 2.07)
Milk intake (ref: <1 glasses)	During pregnancy				

1 – <2 glasses		0.03 (-0.05, 0.10)	-0.03 (-0.12, 0.07)	0.98 (0.65, 1.49)	0.83 (0.58, 1.18)
2 – <3 glasses		0.02 (-0.05, 0.09)	-0.01 (-0.14, 0.11)	0.91 (0.61, 1.37)	0.71 (0.46, 1.11)
≥3 glasses		0.05 (-0.03, 0.13)	0.04 (-0.10, 0.19)	1.27 (0.83, 1.92)	0.98 (0.60, 1.61)
Maternal mental health and parenting					
The timing of the introduction of solid foods (ref: ≥5 months)	During infancy				
4-<5 months		-	-	1.12 (0.73, 1.74)	1.21 (0.75, 1.95)
<4 months		-	-	1.08 (0.61, 1.9)	1.29 (0.78, 2.15)
Breastfeeding duration (ref: ≥6 months)	During infancy				
4-<6 months		-	-	1.33 (0.82, 2.14)	0.90 (0.52, 1.57)
2-<4 months		-	-	0.93 (0.59, 1.46)	1.03 (0.67, 1.57)
<2 months		-	-	0.84 (0.54, 1.32)	0.96 (0.63, 1.45)
Pressure to eat (6 unit of sum score)	4 years	-0.04 (-0.07, 0.00)	-0.07 (-0.13, 0.00)	-	-
Restriction (9 units of sum score)	10 years	0.16 (0.11, 0.21)	0.19 (0.12, 0.27)	1.75 (1.32, 2.32)	1.43 (1.11, 1.84)
Early-life weight and weight gain					
Weight gain during infancy (1110 g)	0 to 6 months	0.05 (0.00, 0.09)	0.08 (0.01, 0.14)	-	-
Weight gain during infancy (760 g)	6 to 12 months	0.05 (0.01, 0.09)	0.08 (0.03, 0.14)	1.1 (0.92, 1.31)	1.26 (1.03, 1.55)
Weight gain during infancy (1100 g)	12 to 24 months	0.08 (0.04, 0.12)	0.13 (0.07, 0.19)	1.21 (1.01, 1.46)	1.35 (1.1, 1.64)
zBMI (1.3 SD)	1.5 months	0.06 (0.01, 0.11)	0.1 (0.03, 0.17)	1.35 (1.09, 1.67)	1.26 (1.01, 1.56)
zBMI (1.4 SD)	2 years	0.24 (0.19, 0.29)	0.33 (0.25, 0.42)	1.79 (1.41, 2.26)	1.93 (1.54, 2.42)
BMI at estimated adiposity peak (1.1 kg/m^2)	-	0.21 (0.15, 0.27)	0.13 (0.05, 0.21)	-	-
Infant and childhood nutrition					
High fat-mass dietary pattern (1.2 unit of component scores)	1 year	0.06 (0.01, 0.11)	0.05 (-0.02, 0.13)	1.19 (0.92, 1.54)	1.12 (0.91, 1.37)
Animal and plant protein intake (15.8 g)	1 year	0.03 (-0.01, 0.07)	0.02 (-0.04, 0.07)	1.12 (0.89, 1.42)	1.05 (0.9, 1.23)
Child behaviors					
Enjoyment of food (4 units of sum score)	4 years	-0.06 (-0.1, -0.01)	-0.04 (-0.1, 0.02)	-	-
Emotional overeating (4 units of sum score)	10 years	-	-	1.13 (0.92, 1.38)	1.14 (0.93, 1.4)
Food responsiveness (5 units of sum score)	10 years	0.24 (0.20, 0.27)	0.26 (0.20, 0.32)	2.27 (1.86, 2.76)	2.09 (1.72, 2.53)
Satiety responsiveness (8 units of sum score)	10 years	-0.18 (-0.22, -0.14)	-0.19 (-0.25, -0.13)	0.64 (0.52, 0.80)	0.64 (0.52, 0.78)
Breakfast skipping (ref: no)	6 years	0.12 (-0.03, 0.28)	0.27 (0.12, 0.41)	1.24 (0.59, 2.62)	2.02 (1.32, 3.09)
Television viewing (ref: <2 hours/day)	6 years	0.07 (0.01, 0.12)	0.08 (-0.02, 0.19)	-	-
Outdoor play (ref: >1 hour/day)	6 years	-	-	1.14 (0.71, 1.81)	0.81 (0.56, 1.16)
Set-shifting (10 units of sum score)	4 years	-0.05 (-0.09, -0.02)	-0.07 (-0.13, -0.01)	0.86 (0.7, 1.06)	0.81 (0.65, 1.02)

**Explained variance (R^2)**		46.1% (43.2%, 48.9%)	48.9% (45.5%, 52.2%)	33.5%[a]	30.2%[a]

Predictors selected in at least 10% DSA models were identified as key predictors. Since the DSA algorithm was performed separately for zBMI and weight status, a set of key predictors was identified for each outcome. These key predictors were then included simultaneously in the multiple linear regression model for zBMI and the logistic regression model for weight status, respectively. Predictors not modeled in a given outcome were marked with a '-' in the table.

For continuous variables, β represents differences in zBMI per interquartile range increase in predictors; for categorical variables, β represents differences of zBMI in comparison category vs. reference categories. For continuous variables, *OR* represent differences in odds of overweight and obesity per interquartile range increase in predictors; for categorical variables, *OR* represent differences of odds of overweight and obesity in comparison category vs. reference categories. IQR and reference categories are indicated in the bracket for predictors. Models include all key predictors selected by the DSA algorithm.

[a] McFadden's pseudo R^2 was calculated for explained variance in the odds of being overweight and obesity at the age of 10 years.

Table S18. Associations between predictors and zBMI or overweight and obesity at age 10 years in parsimonious multiple regression models (N = 5686)

Predictors (IQR or reference category)	Time of assessment	zBMI	Overweight or obesity
		β (95% CI)	*OR* (95% CI)
Sociodemographic factors			
Child ethnic background (ref: Dutch)	Baseline		
Non-Dutch Western		-	2.16 (1.60, 2.92)
Non-Dutch Non-Western		-	2.37 (1.94, 2.88)
Maternal education (ref: high)	Baseline		
Middle		-	1.68 (1.33, 2.12)
Low		-	2.34 (1.78, 3.07)
Paternal education (ref: high)	Baseline		
Middle		0.18 (0.13, 0.24)	1.54 (1.18, 2.00)
Low		0.26 (0.20, 0.32)	1.58 (1.18, 2.13)
Preconception and prenatal parental health			
Maternal BMI (4.4 kg/m^2)	Before pregnancy	0.19 (0.15, 0.23)	1.49 (1.35, 1.65)
Paternal BMI (4.2 kg/m^2)	Baseline	0.19 (0.16, 0.22)	1.50 (1.31, 1.72)
Maternal nutrition during pregnancy			
Vitamin D status, 25(OH)D (48.4 nmol/L)	During pregnancy	-0.14 (-0.18, -0.10)	-
Maternal mental health and parenting			
Restriction (9 units of sum score)	10 years	0.18 (0.14, 0.23)	1.59 (1.34, 1.88)
Early-life weight and weight gain			
Weight gain during infancy (1100 g)	12 to 24 months	-	1.28 (1.12, 1.45)
zBMI (1.4 SD)	2 years	0.34 (0.29, 0.39)	2.01 (1.71, 2.35)
BMI at estimated adiposity peak (1.1 kg/m^2)	-	0.24 (0.20, 0.29)	-
Infant and childhood nutrition			
High fat-mass dietary pattern (1.2 unit of component scores)	1 year	0.07 (0.04, 0.11)	-
Child behaviors			
Food responsiveness (5 units of sum score)	10 years	0.25 (0.22, 0.29)	2.18 (1.94, 2.44)
Satiety responsiveness (8 units of sum score)	10 years	-0.19 (-0.22, -0.16)	0.64 (0.55, 0.73)
**Explained variance (R^2)**		46.4% (44.0%, 48.6%)	31.3%[a]

DSA algorithm was performed in a parsimonious way based on cross-validation risk plots, allowing up to 10 variables in each model. Predictors selected in at least 10% DSA models were identified as key predictors. Since the DSA algorithm was performed separately for zBMI and weight status, a set of key predictors was identified for each outcome. These key predictors were then included simultaneously in the multiple linear regression model for zBMI and the logistic regression model for weight status, respectively. Predictors not modeled in a given outcome were marked with a '-' in the table.

For continuous variables, β represent differences in zBMI per interquartile range increase in predictors; for categorical variables, β represent differences of zBMI in comparison category vs. reference categories. For continuous variables, *OR* represent differences in odds of overweight and obesity per interquartile range increase in predictors; for categorical variables, *OR* represent differences of odds of overweight and obesity in comparison category vs. reference categories. IQR and reference categories are indicated in the bracket for predictors. Models include all key predictors selected by the DSA algorithm and child BMI polygenic risk scores.

[a] McFadden's pseudo R^2 was calculated for explained variance in the odds of being overweight and obesity at the age of 10 years.

Reference list of publications included in the evidence synthesis

1 Ay L, Hokken-Koelega AC, Mook-Kanamori DO, et al. Tracking and determinants of subcutaneous fat mass in early childhood: the Generation R Study. *Int J Obes (Lond)*. 2008; 32: 1050-9.

2 Benschop L, Schalekamp-Timmermans S, Roeters van Lennep JE, et al. Cardiovascular Risk Factors Track From Mother to Child. *J Am Heart Assoc*. 2018; 7: e009536.

3 Bouthoorn SH, van Lenthe FJ, Kiefte-de Jong JC, et al. Genetic taste blindness to bitter and body composition in childhood: a Mendelian randomization design. *Int J Obes (Lond)*. 2014; 38: 1005-10.

4 Bouthoorn SH, Wijtzes AI, Jaddoe VW, et al. Development of socioeconomic inequalities in obesity among Dutch pre-school and school-aged children. *Obesity (Silver Spring)*. 2014; 22: 2230-7.

5 Bowling AB, Tiemeier HW, Jaddoe VWV, Barker ED, Jansen PW. ADHD symptoms and body composition changes in childhood: a longitudinal study evaluating directionality of associations. *Pediatr Obes*. 2018; 13: 567-75.

6 Braun KV, Erler NS, Kiefte-de Jong JC, et al. Dietary Intake of Protein in Early Childhood Is Associated with Growth Trajectories between 1 and 9 Years of Age. *J Nutr*. 2016; 146: 2361-67.

7 Braun KV, Voortman T, Kiefte-de Jong JC, et al. Dietary Intakes of Folic Acid and Methionine in Early Childhood Are Associated with Body Composition at School Age. *J Nutr*. 2015; 145: 2123-9.

8 Cajachagua-Torres KN, El Marroun H, Reiss IKM, Santos S, Jaddoe VWV. Foetal tobacco and cannabis exposure, body fat and cardio-metabolic health in childhood. *Pediatr Obes*. 2022; 17: e12863.

9 Camfferman R, Jansen PW, Rippe RCA, et al. The association between overweight and internalizing and externalizing behavior in early childhood. *Social Science & Medicine*. 2016; 168: 35-42.

10 de Barse LM, Tiemeier H, Leermakers ET, et al. Longitudinal association between preschool fussy eating and body composition at 6 years of age: The Generation R Study. *Int J Behav Nutr Phys Act*. 2015; 12: 153.

11 Derks IP, Tiemeier H, Sijbrands EJ, et al. Testing the direction of effects between child body composition and restrictive feeding practices: results from a population-based cohort. *Am J Clin Nutr*. 2017; 106: 783-90.

12 Derks IPM, Bolhuis K, Yalcin Z, et al. Testing Bidirectional Associations Between Childhood Aggression and BMI: Results from Three Cohorts. 2019.

13 Derks IPM, Kocevska D, Jaddoe VWV, et al. Longitudinal Associations of Sleep Duration in Infancy and Early Childhood with Body Composition and Cardiometabolic Health at the Age of 6 Years: The Generation R Study. *Child Obes*. 2017; 13: 400-08.

14 Derks IPM, Sijbrands EJG, Wake M, et al. Eating behavior and body composition across childhood: a prospective cohort study. *Int J Behav Nutr Phys Act*. 2018; 15: 96.

15 Durmus B, Arends LR, Ay L, et al. Parental anthropometrics, early growth and the risk of overweight in pre-school children: the Generation R Study. *Pediatr Obes*. 2013; 8: 339-50.

16 Durmus B, Ay L, Hokken-Koelega AC, et al. Maternal smoking during pregnancy and subcutaneous fat mass in early childhood. The Generation R Study. *Eur J Epidemiol*. 2011; 26: 295-304.
17 Durmus B, Heppe DH, Gishti O, et al. General and abdominal fat outcomes in school-age children associated with infant breastfeeding patterns. *Am J Clin Nutr*. 2014; 99: 1351-8.
18 Durmus B, Heppe DH, Taal HR, et al. Parental smoking during pregnancy and total and abdominal fat distribution in school-age children: the Generation R Study. *Int J Obes (Lond)*. 2014; 38: 966-72.
19 Durmus B, Kruithof CJ, Gillman MH, et al. Parental smoking during pregnancy, early growth, and risk of obesity in preschool children: the Generation R Study. *Am J Clin Nutr*. 2011; 94: 164-71.
20 Elhakeem A, Taylor AE, Inskip HM, et al. Association of Assisted Reproductive Technology With Offspring Growth and Adiposity From Infancy to Early Adulthood. *JAMA Netw Open*. 2022; 5: e2222106.
21 Ertel KA, Kleinman K, van Rossem L, et al. Maternal perinatal depression is not independently associated with child body mass index in the Generation R Study: methods and missing data matter. *J Clin Epidemiol*. 2012; 65: 1300-9.
22 Gaillard R, Rurangirwa AA, Williams MA, et al. Maternal Parity, fetal and childhood growth, and cardiometabolic risk factors. *Hypertension*. 2014; 64: 266-74.
23 Gaillard R, Steegers EA, Duijts L, et al. Childhood cardiometabolic outcomes of maternal obesity during pregnancy: the Generation R Study. *Hypertension*. 2014; 63: 683-91.
24 Gaillard R, Steegers EA, Franco OH, Hofman A, Jaddoe VW. Maternal weight gain in different periods of pregnancy and childhood cardio-metabolic outcomes. The Generation R Study. *Int J Obes (Lond)*. 2015; 39: 677-85.
25 Gaillard R, Steegers EA, Tiemeier H, Hofman A, Jaddoe VW. Placental vascular dysfunction, fetal and childhood growth, and cardiovascular development: the generation R study. *Circulation*. 2013; 128: 2202-10.
26 Gishti O, Gaillard R, Felix JF, et al. Early origins of ethnic disparities in cardiovascular risk factors. *Prev Med*. 2015; 76: 84-91.
27 Gishti O, Gaillard R, Manniesing R, et al. Fetal and infant growth patterns associated with total and abdominal fat distribution in school-age children. *J Clin Endocrinol Metab*. 2014; 99: 2557-66.
28 Gishti O, Kruithof CJ, Felix JF, et al. Ethnic disparities in general and abdominal adiposity at school age: a multiethnic population-based cohort study in the Netherlands. *Ann Nutr Metab*. 2014; 64: 208-17.
29 Godoy GA, Korevaar TI, Peeters RP, et al. Maternal thyroid hormones during pregnancy, childhood adiposity and cardiovascular risk factors: the Generation R Study. *Clin Endocrinol (Oxf)*. 2014; 81: 117-25.
30 Gootjes DV, Posthumus AG, Jaddoe VWV, van Rijn BB, Steegers EAP. Maternal hypertensive disorders in pregnancy and early childhood cardiometabolic risk factors: The Generation R Study. *PLoS One*. 2021; 16: e0261351.
31 Guxens M, Tiemeier H, Jansen PW, et al. Parental psychological distress during pregnancy and early growth in preschool children: the generation R study. *Am J Epidemiol*. 2013; 177: 538-47.
32 Harris HA, Bowling A, Santos S, Greaves-Lord K, Jansen PW. Child ADHD and autistic traits, eating behaviours and weight: A population-based study. *Pediatr Obes*. 2022; 17.

33 Jaddoe VW, de Jonge LL, Hofman A, et al. First trimester fetal growth restriction and cardiovascular risk factors in school age children: population based cohort study. *Bmj*. 2014; 348: g14.
34 Jansen MA, Kiefte-de Jong JC, Gaillard R, et al. Growth trajectories and bone mineral density in anti-tissue transglutaminase antibody-positive children: the Generation R Study. *Clin Gastroenterol Hepatol*. 2015; 13: 913-20 e5.
35 Jansen PW, Derks IPM, Batenburg A, et al. Using Food to Soothe in Infancy is Prospectively Associated with Childhood BMI in a Population-Based Cohort. *J Nutr*. 2019; 149: 788-94.
36 Jansen PW, Derks IPM, Mou YC, et al. Associations of parents' use of food as reward with children's eating behaviour andBMIin a population-based cohort. *Pediatr Obes*. 2020; 15.
37 Jansen PW, Roza SJ, Jaddoe VW, et al. Children's eating behavior, feeding practices of parents and weight problems in early childhood: results from the population-based Generation R Study. *Int J Behav Nutr Phys Act*. 2012; 9: 130.
38 Jansen PW, Tharner A, van der Ende J, et al. Feeding practices and child weight: is the association bidirectional in preschool children? *Am J Clin Nutr*. 2014; 100: 1329-36.
39 Jelena Vidakovic A, Santos S, Williams MA, et al. Maternal plasma n-3 and n-6 polyunsaturated fatty acid concentrations during pregnancy and subcutaneous fat mass in infancy. *Obesity (Silver Spring)*. 2016; 24: 1759-66.
40 Jen V, Braun KVE, Karagounis LG, et al. Longitudinal association of dietary protein intake in infancy and adiposity throughout childhood. *Clin Nutr*. 2019; 38: 1296-302.
41 Jen V, Erler NS, Tielemans MJ, et al. Mothers' intake of sugar-containing beverages during pregnancy and body composition of their children during childhood: the Generation R Study. *Am J Clin Nutr*. 2017; 105: 834-41.
42 Jharap VV, Santos S, Steegers EAP, Jaddoe VWV, Gaillard R. Associations of maternal obesity and excessive weight gain during pregnancy with subcutaneous fat mass in infancy. *Early Hum Dev*. 2017; 108: 23-28.
43 Kooijman MN, Gaillard R, Reiss I, et al. Influence of fetal blood flow redistribution on fetal and childhood growth and fat distribution: the Generation R Study. *Bjog*. 2016; 123: 2104-12.
44 Kruithof CJ, Gishti O, Hofman A, Gaillard R, Jaddoe VW. Infant weight growth velocity patterns and general and abdominal adiposity in school-age children. The Generation R Study. *Eur J Clin Nutr*. 2016; 70: 1144-50.
45 Lecorguillé M, Schipper MC, O'Donnell A, et al. Impact of parental lifestyle patterns in the preconception and pregnancy periods on childhood obesity. *Frontiers in Nutrition*. 2023; 10.
46 Leermakers ET, Felix JF, Erler NS, et al. Sugar-containing beverage intake in toddlers and body composition up to age 6 years: the Generation R study. *Eur J Clin Nutr*. 2015; 69: 314-21.
47 Leermakers ET, Felix JF, Jaddoe VW, et al. Sugar-containing beverage intake at the age of 1 year and cardiometabolic health at the age of 6 years: the Generation R Study. *Int J Behav Nutr Phys Act*. 2015; 12: 114.
48 Leermakers ET, Kiefte-de Jong JC, Hofman A, Jaddoe VW, Franco OH. Lutein intake at the age of 1 year and cardiometabolic health at the age of 6 years: the Generation R Study. *Br J Nutr*. 2015; 114: 970-8.

49 Lin LZ, Yang-Huang JW, Wang HJ, et al. Social mobility by parent education and childhood overweight and obesity: a prospective cohort study. *Eur J Public Health*. 2021; 31: 764-70.
50 Maas JA, Mook-Kanamori DO, Ay L, et al. Insulin VNTR and IGF-1 promoter region polymorphisms are not associated with body composition in early childhood: the generation R study. *Horm Res Paediatr*. 2010; 73: 120-7.
51 Mackenbach JD, Tiemeier H, Ende J, et al. Relation of emotional and behavioral problems with body mass index in preschool children: the Generation R study. *J Dev Behav Pediatr*. 2012; 33: 641-8.
52 Marinkovic T, Toemen L, Kruithof CJ, et al. Early Infant Growth Velocity Patterns and Cardiovascular and Metabolic Outcomes in Childhood. *J Pediatr*. 2017; 186: 57-63 e4.
53 Miliku K, Felix JF, Voortman T, et al. Associations of maternal and fetal vitamin D status with childhood body composition and cardiovascular risk factors. *Matern Child Nutr*. 2018; 15: e12672.
54 Monasso GS, Santos S, Geurtsen ML, et al. Associations of Early Pregnancy and Neonatal Circulating Folate, Vitamin B-12, and Homocysteine Concentrations with Cardiometabolic Risk Factors in Children at 10 y of Age. *J Nutr*. 2021; 151: 1628-36.
55 Monnereau C, Jansen PW, Tiemeier H, Jaddoe VW, Felix JF. Influence of genetic variants associated with body mass index on eating behavior in childhood. *Obesity (Silver Spring)*. 2017; 25: 765-72.
56 Monnereau C, Santos S, van der Lugt A, Jaddoe VWV, Felix JF. Associations of adult genetic risk scores for adiposity with childhood abdominal, liver and pericardial fat assessed by magnetic resonance imaging. *Int J Obes (Lond)*. 2018; 42: 897-904.
57 Monnereau C, Vogelezang S, Kruithof CJ, Jaddoe VW, Felix JF. Associations of genetic risk scores based on adult adiposity pathways with childhood growth and adiposity measures. *BMC Genet*. 2016; 17: 120.
58 Nguyen AN, Jen V, Jaddoe VWV, et al. Diet quality in early and mid-childhood in relation to trajectories of growth and body composition. *Clin Nutr*. 2020; 39: 845-52.
59 Nguyen AN, Santos S, Braun KVE, Voortman T. Carbohydrate Intake in Early Childhood and Body Composition and Metabolic Health: Results from the Generation R Study. *Nutrients*. 2020; 12.
60 Noppe G, van den Akker EL, de Rijke YB, et al. Long-term glucocorticoid concentrations as a risk factor for childhood obesity and adverse body-fat distribution. *Int J Obes (Lond)*. 2016; 40: 1503-09.
61 Patro Golab B, Voerman E, van der Lugt A, Santos S, Jaddoe VWV. Subcutaneous fat mass in infancy and abdominal, pericardial and liver fat assessed by Magnetic Resonance Imaging at the age of 10 years. *Int J Obes (Lond)*. 2019; 43: 392-401.
62 Poeran-Bahadoer S, Jaddoe VWV, Gishti O, et al. Maternal vomiting during early pregnancy and cardiovascular risk factors at school age: the Generation R Study. *J Dev Orig Health Dis*. 2020; 11: 118-26.
63 Quezada-Pinedo HG, Jaddoe V, Duijts L, et al. Maternal iron status in early pregnancy and childhood body fat measures and cardiometabolic risk factors: A population-based prospective cohort. *Am J Clin Nutr*. 2023; 117: 191-98.
64 Richmond RC, Timpson NJ, Felix JF, et al. Using Genetic Variation to Explore the Causal Effect of Maternal Pregnancy Adiposity on Future Offspring Adiposity: A Mendelian Randomisation Study. *PLoS Med*. 2017; 14: e1002221.

65 Santos S, Gaillard R, Oliveira A, et al. Associations of Infant Subcutaneous Fat Mass with Total and Abdominal Fat Mass at School-Age: The Generation R Study. *Paediatr Perinat Epidemiol*. 2016; 30: 511-20.
66 Santos S, Monnereau C, Felix JF, et al. Maternal body mass index, gestational weight gain, and childhood abdominal, pericardial, and liver fat assessed by magnetic resonance imaging. *Int J Obes (Lond)*. 2019; 43: 581-93.
67 Steegers C, Dieleman G, Moskalenko V, et al. The longitudinal relationship between set-shifting at 4 years of age and eating disorder related features at 9 years of age in the general pediatric population. *Int J Eating Disord*. 2021.
68 Stroobant W, Braun KV, Kiefte-de Jong JC, et al. Intake of Different Types of Fatty Acids in Infancy Is Not Associated with Growth, Adiposity, or Cardiometabolic Health up to 6 Years of Age. *J Nutr*. 2017; 147: 413-20.
69 Taal HR, Vd Heijden AJ, Steegers EA, Hofman A, Jaddoe VW. Small and large size for gestational age at birth, infant growth, and childhood overweight. *Obesity (Silver Spring)*. 2013; 21: 1261-8.
70 Tielemans MJ, Steegers EAP, Voortman T, et al. Protein intake during pregnancy and offspring body composition at 6 years: the Generation R Study. *Eur J Nutr*. 2017; 56: 2151-60.
71 van den Broek M, Leermakers ET, Jaddoe VW, et al. Maternal dietary patterns during pregnancy and body composition of the child at age 6 y: the Generation R Study. *Am J Clin Nutr*. 2015; 102: 873-80.
72 van Rossem L, Silva LM, Hokken-Koelega A, et al. Socioeconomic status is not inversely associated with overweight in preschool children. *J Pediatr*. 2010; 157: 929-35 e1.
73 Vehmeijer FOL, C CVS, Derks IPM, et al. Associations of Maternal Psychological Distress during Pregnancy with Childhood General and Organ Fat Measures. *Child Obes*. 2019; 15: 313-22.
74 Vehmeijer FOL, Santos S, Gaillard R, et al. Associations of Hair Cortisol Concentrations with General and Organ Fat Measures in Childhood. *J Clin Endocrinol Metab*. 2021; 106: e551-e61.
75 Velders FP, De Wit JE, Jansen PW, et al. FTO at rs9939609, food responsiveness, emotional control and symptoms of ADHD in preschool children. *PLoS One*. 2012; 7: e49131.
76 Vidakovic AJ, Gishti O, Voortman T, et al. Maternal plasma PUFA concentrations during pregnancy and childhood adiposity: the Generation R Study. *Am J Clin Nutr*. 2016; 103: 1017-25.
77 Vinther JL, Cadman T, Avraam D, et al. Gestational age at birth and body size from infancy through adolescence: An individual participant data meta-analysis on 253,810 singletons in 16 birth cohort studies. *PLoS Med*. 2023; 20: e1004036.
78 Voerman E, Gaillard R, Geurtsen ML, Jaddoe VWV. Maternal First-Trimester Cow-Milk Intake Is Positively Associated with Childhood General and Abdominal Visceral Fat Mass and Lean Mass but Not with Other Cardiometabolic Risk Factors at the Age of 10 Years. *Journal of Nutrition*. 2021; 151: 1965-75.
79 Voerman E, Jaddoe VW, Gishti O, et al. Maternal caffeine intake during pregnancy, early growth, and body fat distribution at school age. *Obesity (Silver Spring)*. 2016; 24: 1170-7.
80 Voerman E, Jaddoe VW, Hulst ME, Oei EH, Gaillard R. Associations of maternal caffeine intake during pregnancy with abdominal and liver fat deposition in childhood. *Pediatr Obes*. 2020; 15: e12607.

81 Vogelezang S, Gishti O, Felix JF, et al. Tracking of abdominal subcutaneous and preperitoneal fat mass during childhood. The Generation R Study. *Int J Obes (Lond)*. 2016; 40: 595-600.
82 Vogelezang S, Monnereau C, Gaillard R, et al. Adult adiposity susceptibility loci, early growth and general and abdominal fatness in childhood: the Generation R Study. *Int J Obes (Lond)*. 2015; 39: 1001-9.
83 Vogelezang S, Santos S, Toemen L, et al. Associations of Fetal and Infant Weight Change With General, Visceral, and Organ Adiposity at School Age. *JAMA Netw Open*. 2019; 2: e192843.
84 Vogelezang S, Santos S, van der Beek EM, et al. Infant breastfeeding and childhood general, visceral, liver, and pericardial fat measures assessed by magnetic resonance imaging. *Am J Clin Nutr*. 2018; 108: 722-29.
85 Voortman T, Braun KV, Kiefte-de Jong JC, et al. Protein intake in early childhood and body composition at the age of 6 years: The Generation R Study. *Int J Obes (Lond)*. 2016; 40: 1018-25.
86 Voortman T, Leermakers ET, Franco OH, et al. A priori and a posteriori dietary patterns at the age of 1 year and body composition at the age of 6 years: the Generation R Study. *Eur J Epidemiol*. 2016; 31: 775-83.
87 Voortman T, Tielemans MJ, Stroobant W, et al. Plasma fatty acid patterns during pregnancy and child's growth, body composition, and cardiometabolic health: The Generation R Study. *Clin Nutr*. 2018; 37: 984-92.
88 Wahab RJ, Jaddoe VWV, Gaillard R. Associations of maternal early-pregnancy dietary glycemic index with childhood general, abdominal and ectopic fat accumulation. *Clin Nutr*. 2021; 40: 1628-36.
89 Wahab RJ, Voerman E, Jansen PW, et al. Maternal Glucose Concentrations in Early Pregnancy and Cardiometabolic Risk Factors in Childhood. *Obesity (Silver Spring)*. 2020; 28: 985-93.
90 Welten M, Gaillard R, Hofman A, de Jonge LL, Jaddoe VW. Maternal haemoglobin levels and cardio-metabolic risk factors in childhood: the Generation R study. *Bjog*. 2015; 122: 805-15.
91 Wijtzes AI, Bouthoorn SH, Jansen W, et al. Sedentary behaviors, physical activity behaviors, and body fat in 6-year-old children: the generation R study. *Int J Behav Nutr Phys Act*. 2014; 11: 96.
92 Wijtzes AI, Jansen W, Bouthoorn SH, et al. Meal-Skipping Behaviors and Body Fat in 6-Year-Old Children. *J Pediatr*. 2016; 168: 118-25 e2.
93 Wijtzes AI, Kooijman MN, Kiefte-de Jong JC, et al. Correlates of physical activity in 2-year-old toddlers: the generation R study. *J Pediatr*. 2013; 163: 791-9 e1-2.

A) Pooled effect sizes for BMI at 2 and 3 years

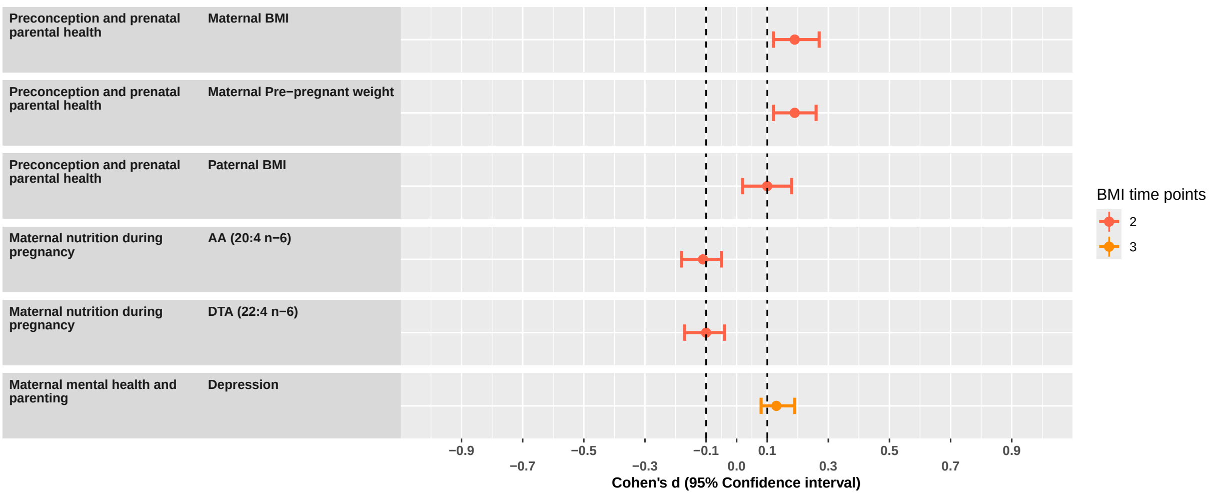

B) Pooled effect sizes for BMI at 4 years

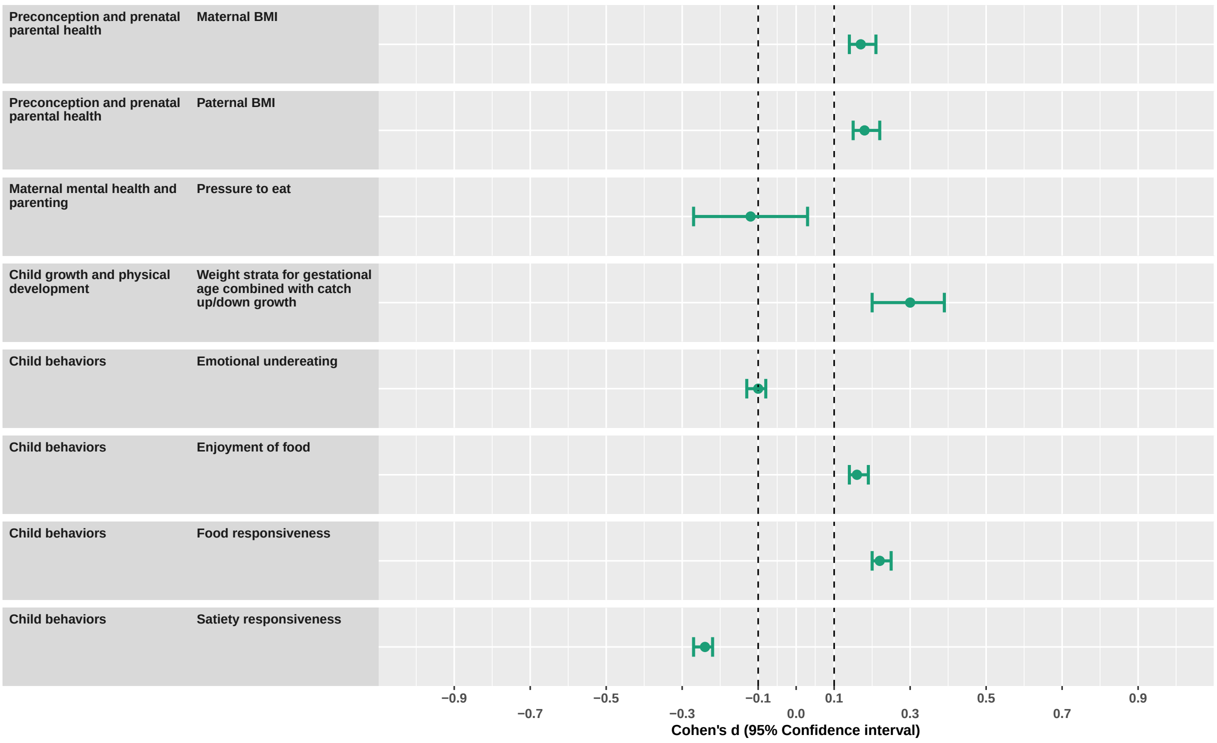

C) Pooled effect sizes for BMI at 6 years

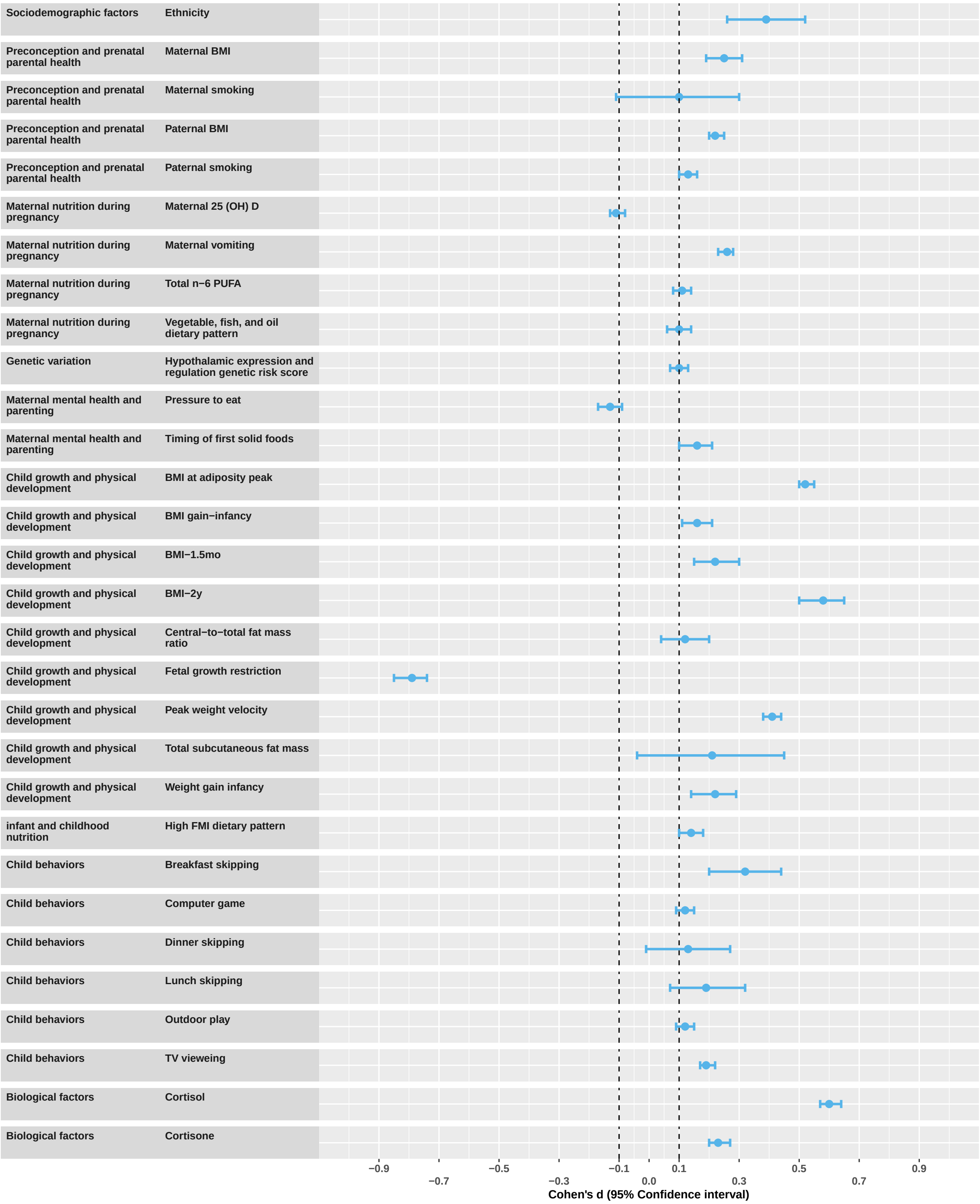

D) Pooled effect sizes for BMI at 10 years

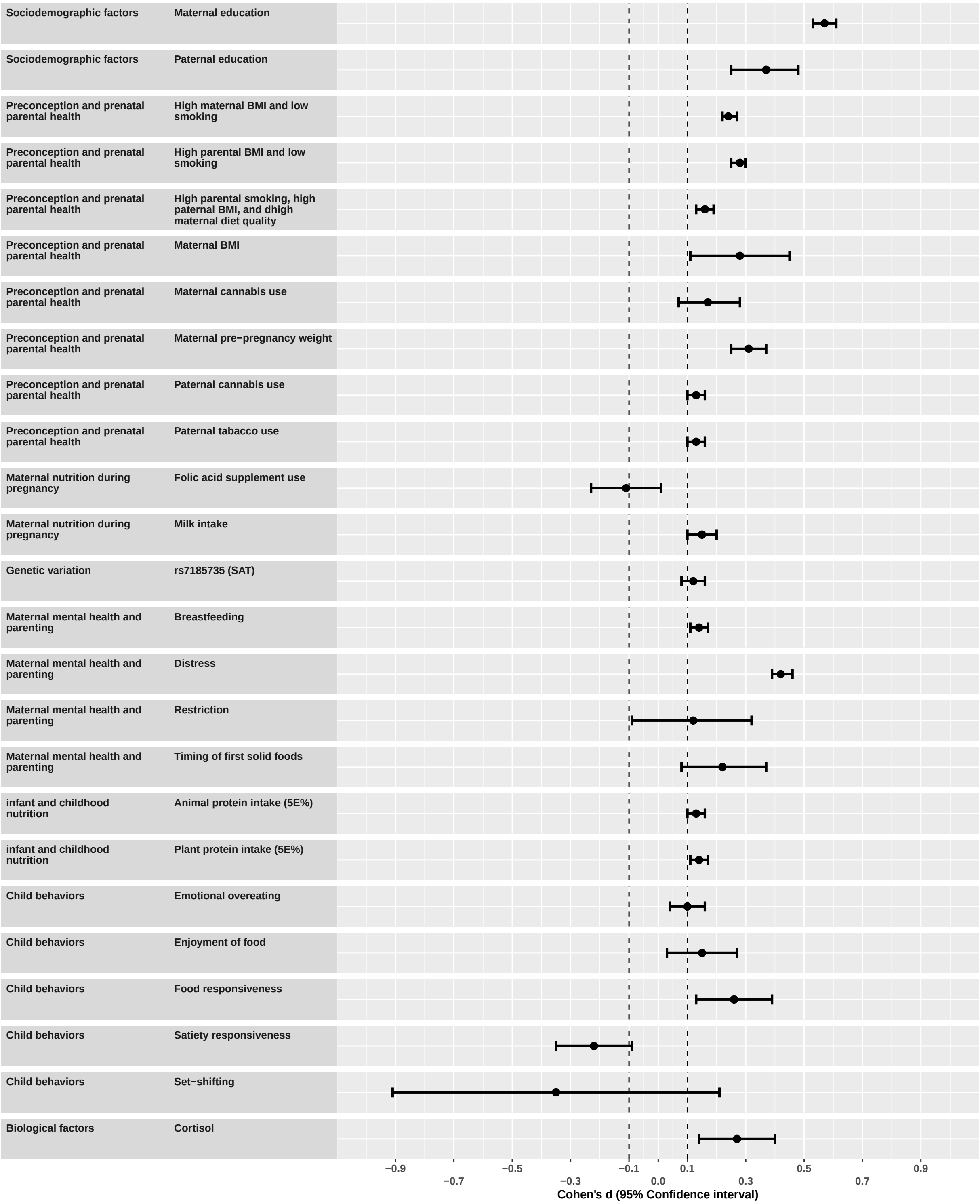

Figure S1. Forest plots for predictors of child BMI with pooled effect sizes above the threshold ($|d| \geq 0.1$)

A) BMI

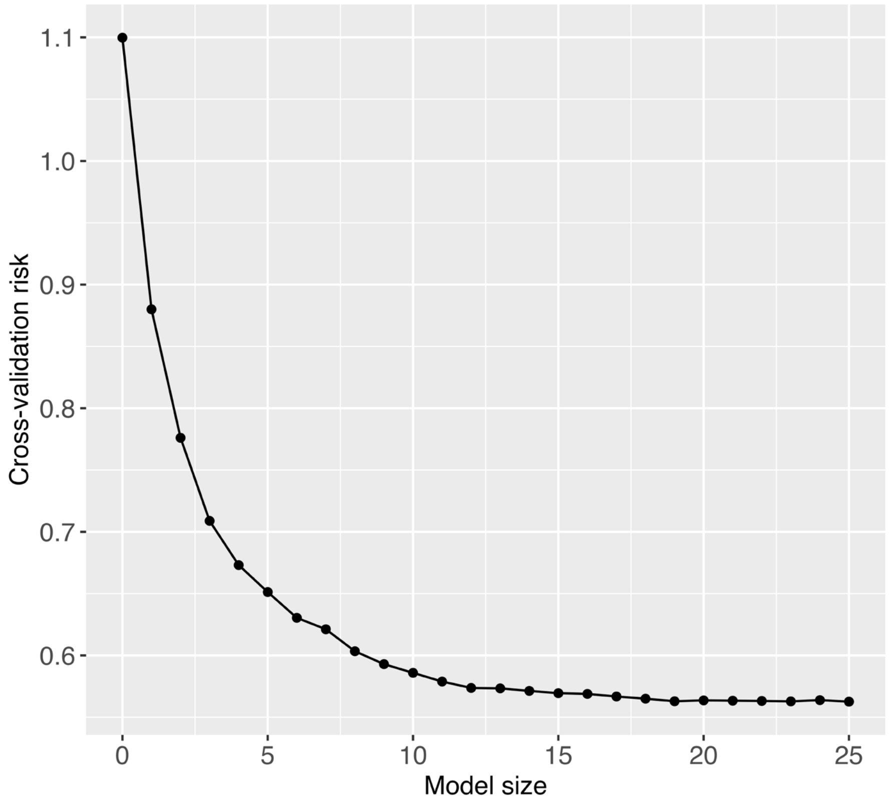

B) Overewight and obesity

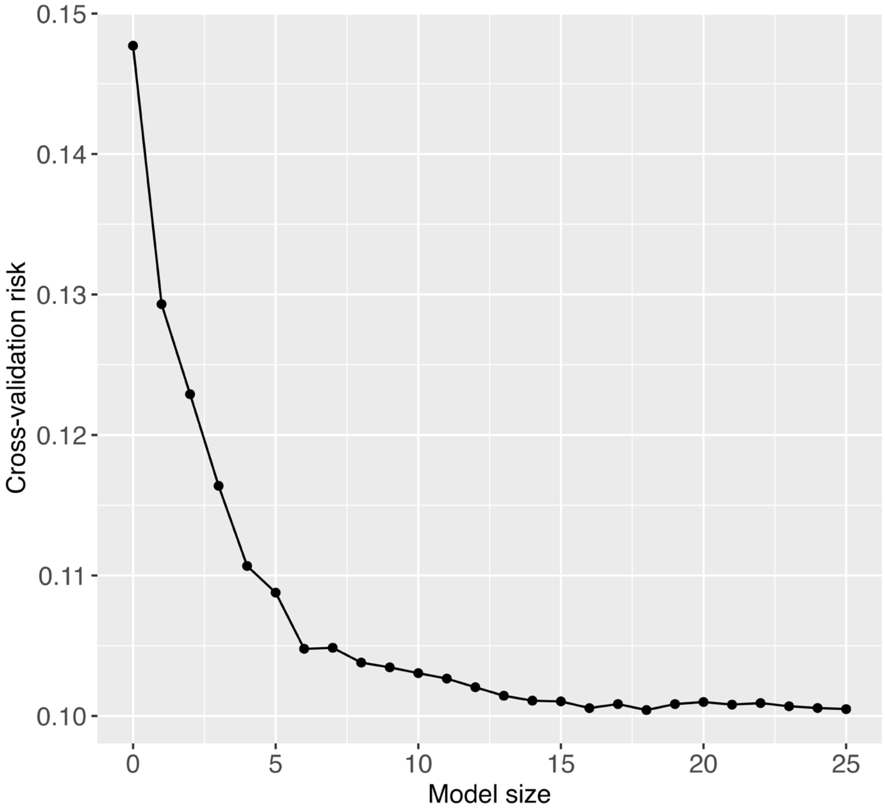

Figure S2. Cross-validation plots based on DSA-selected models for (A) BMI and (B) overweight and obesity in children aged 10 years
